# Sertoli cells are the source of stem cell factor for spermatogenesis

**DOI:** 10.1242/dev.200706

**Published:** 2023-03-20

**Authors:** Yi Jacky Peng, Xinyu Thomas Tang, Hui Sophie Shu, Wenjie Dong, Hongfang Shao, Bo O. Zhou

**Affiliations:** ^1^ State Key Laboratory of Cell Biology, Shanghai Institute of Biochemistry and Cell Biology, CAS Center for Excellence in Molecular Cell Science, Chinese Academy of Sciences; University of Chinese Academy of Sciences, 320 Yueyang Road, Shanghai, 200031, People's Republic of China; ^2^ State Key Laboratory of Experimental Hematology, Haihe Laboratory of Cell Ecosystem, Institute of Hematology & Blood Diseases Hospital, Chinese Academy of Medical Sciences & Peking Union Medical College, Tianjin, 300020, People's Republic of China; ^3^ Center of Reproductive Medicine, Department of Gynecology and Obstetrics, Shanghai Jiao Tong University School of Medicine-Affiliated Sixth People's Hospital, 600 Yishan Road, Shanghai, 200233, People's Republic of China

**Keywords:** Spermatogenesis, Stem cell factor, Sertoli cell, Testis

## Abstract

Several cell types have been proposed to create the required microenvironment for spermatogenesis. However, expression patterns of the key growth factors produced by these somatic cells have not been systematically studied and no such factor has been conditionally deleted from its primary source(s), raising the question of which cell type(s) are the physiological sources of these growth factors. Here, using single-cell RNA sequencing and a series of fluorescent reporter mice, we found that stem cell factor (*Scf*), one of the essential growth factors for spermatogenesis, was broadly expressed in testicular stromal cells, including Sertoli, endothelial, Leydig, smooth muscle and *Tcf21-*CreER^+^ stromal cells. Both undifferentiated and differentiating spermatogonia were associated with *Scf*-expressing Sertoli cells in the seminiferous tubule. Conditional deletion of *Scf* from Sertoli cells, but not any other *Scf*-expressing cells, blocked the differentiation of spermatogonia, leading to complete male infertility. Conditional overexpression of *Scf* in Sertoli cells, but not endothelial cells, significantly increased spermatogenesis. Our data reveal the importance of anatomical localization for Sertoli cells in regulating spermatogenesis and that SCF produced specifically by Sertoli cells is essential for spermatogenesis.

## Teaser

**Summary:** SCF is produced by Sertoli, endothelial, Leydig, smooth muscle cells and Tcf21-CreER^+^ stromal cells in the testis, but Sertoli cells are the only physiologically important source of SCF that contributes to spermatogenesis.

## INTRODUCTION

Mammalian spermatogenesis is a complex developmental process based on a robust spermatogonial stem cell (SSC) system. Self-renewal and differentiation of SSCs are regulated by factors secreted from testicular somatic cells (Chen et al., 2016; Kitadate et al., 2019; Oatley and Brinster, 2012; van Pelt and de Rooij, 1990). Sertoli cells have been regarded as a key component of the spermatogonial niche (Oatley et al., 2011). This is supported by findings that Sertoli cell-specific deletion of transcription factor-encoding genes, such as *Rbpj* (Garcia et al., 2014), *Sin3a* (Payne et al., 2010) and *Gata4* (Kyrönlahti et al., 2011), or hormone receptors, such as *Ar* (De Gendt et al., 2004), *Lifr* (Curley et al., 2018) and *Pgrmc1* (Broady et al., 2011), impaired spermatogenesis and male fertility. However, there was no evidence that any of the above genes directly regulate spermatogonia. It is likely that deletion of these genes disrupts the normal properties of Sertoli cells, which indirectly affects spermatogenesis.

Sertoli cells are thought to be the producer of many important growth factors that regulate spermatogenesis, including GDNF (Meng et al., 2000), FGF2 (Mullaney and Skinner, 1992), SCF (Rossi et al., 1993), neuregulin 1 (Zhang et al., 2011), WNT5A (Yeh et al., 2011), activin A (De Winter et al., 1993), BMP4 (Pellegrini et al., 2003), EGF (Radhakrishnan et al., 1992), PDGF (Li et al., 1997; Loveland et al., 1995) and CXCL12 (Yang et al., 2013). Other testicular somatic cells also express factors that regulate SSCs and/or spermatogenesis. Leydig cells express IGF1 (Huang et al., 2009), CSF1 (Oatley et al., 2009) and EGF (Zhang et al., 1997). Testicular endothelial cells express GDNF (Bhang et al., 2018) and FGF5 (Kitadate et al., 2019). Peritubular myoid cells express CSF1 (Oatley et al., 2009), GDNF (Chen et al., 2016) and Activin A (De Winter et al., 1994). Testis-resident macrophages express CSF1 (DeFalco et al., 2015). Despite the advances in identifying candidate growth factors and cell types, expression patterns of these factors in the testis have never been mapped in a systematic way. None of the factors has ever been conditionally deleted from Sertoli cells, or most of other somatic cells in the testis, raising the question of whether these factors directly regulate spermatogenesis, and, if so, which cell type(s) are the physiological source(s) of the growth factor.

Single-cell RNA sequencing (scRNA-seq) provides a high-resolution picture of the transcriptome of a single cell (Ozsolak and Milos, 2011; Tang et al., 2009). It is now widely used to comprehensively identify cell populations within specific tissues, including mouse testis (Green et al., 2018; Grive et al., 2019; Han et al., 2018; Hermann et al., 2018; Jung et al., 2019; Wang et al., 2019). In this study, we used scRNA-seq to map the expression patterns of all growth factors in testicular somatic cells. We found that stem cell factor (*Scf*, also known as *Kitl*), an essential growth factor, showed a much broader expression pattern in the testis than previously recognized, including Sertoli, endothelial, Leydig, smooth muscle and *Tcf21*^+^ stromal cells. The expression pattern of *Scf* in the testis was further confirmed using a *Scf*-GFP reporter. We took genetic approaches to conditionally delete or overexpress *Scf* from *Scf*-expressing cell types. The results showed that Sertoli cells are the physiological source of SCF for spermatogenesis. Finally, we used scRNA-seq to explore the cellular and molecular mechanisms by which SCF regulates spermatogenesis. We found that Sertoli cell-derived SCF plays dual roles in spermatogenesis. It maintains the pool of differentiating spermatogonia and promotes their differentiation.

## RESULTS

### scRNA-seq reveals broad expression of *Scf* among testicular somatic cells

**Fig. 1.**
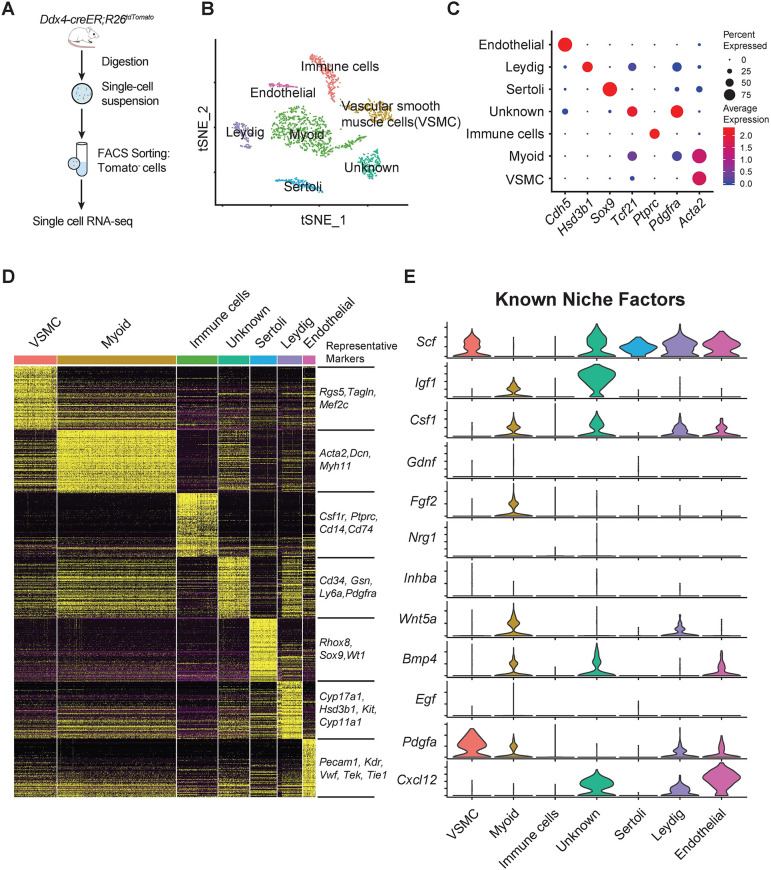
**scRNA-seq reveals the expression patterns of key growth factors in testicular somatic cells.** (A) Schematic overview of the workflow for sample preparation for scRNA-seq. Samples were from three *Ddx4-creER; R26^tdTomato^* mice in three independent experiments. FACS, fluorescence-activated cell sorting. (B) t-SNE and clustering analysis of single-cell transcriptome data from testicular somatic cells. (C) Dot plot showing the expression patterns of distinct cell-specific marker genes in seven somatic cell clusters. (D) Heatmap for the top 100 DEGs in each cell cluster. (E) Violin plots showing the expression levels of different known growth factors in each somatic cell type.

To map systematically the expression patterns of all growth factors required for spermatogenesis, we performed scRNA-seq of all testicular somatic cell types. To enrich somatic cells, we depleted Tomato^+^ cells from the testes of 2-month-old *Ddx4-creER; R26^tdTomato^* mice at 4 weeks after tamoxifen treatment (Fig. 1A); 11,209 cells from a total of 14,039 testicular cells passed standard quality control and were retained for subsequent analyses. Unsupervised clustering identified 17 cell types projected onto a uniform manifold approximation and projection (UMAP) analysis plot (Fig. S1A). Then, the normalized gene expression matrix of 2190 somatic cells was extracted based on the expression patterns of the germ cell marker gene *Ddx4* and the elongating spermatid marker gene *Tnp1* for further analyses (Fig. S1B-D).

Re-clustering and t-distributed stochastic neighbor embedding (t-SNE) analysis of testicular somatic cells identified seven somatic cell types, and cell identities were assigned according to the expression of known cell-specific markers (Fig. 1B-D). We next visualized the expression patterns of related growth factors, including *Scf*, *Igf1*, *Csf1*, *Gdnf*, *Fgf2*, *Nrg1*, *Inhba*, *Wnt5a*, *Bmp4*, *Egf*, *Pdgfa* and *Cxcl12*, in distinct somatic cell types (Fig. 1E; Fig. S1E). As a result, many factors, including *Scf*, *Igf1*, *Csf1*, *Wnt5a*, *Bmp4*, *Pdgfa* and *Cxcl12*, displayed broader expression patterns in the testicular stroma than previously recognized (Fig. 1E). In contrast, some factors, including *Gdnf*, *Nrg1*, *Inhba* and *Egf*, were not detectably expressed by any cell types (Fig. 1E). As expected, *Scf* was expressed by Sertoli cells (Fig. 1E). *Scf* expression was also observed in endothelial cells, Leydig cells, smooth muscle cells and an unknown cell population that also expressed *Tcf21* and *Pdgfra* (Fig. 1C,E). A similar expression pattern of *Scf* was also observed in cynomolgus macaque testis (Fig. S1F-H). The broad expression pattern of *Scf* in the testis raised the possibility that multiple somatic cells contribute to spermatogenesis by secreting SCF.

### *Scf*-GFP is expressed by Sertoli cells in the seminiferous tubule

**Fig. 2.**
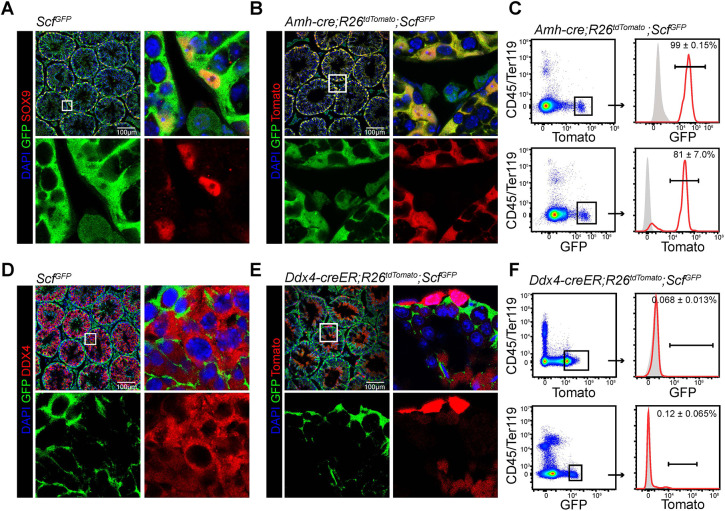
***Scf*-GFP is expressed by Sertoli cells, but not germ cells, in the seminiferous tubules.** (A) Confocal imaging of testis sections from 6-week-old *Scf^GFP^* mice that were stained with anti-GFP and anti-SOX9 antibodies. (*n*=3 mice from 3 independent experiments). (B) Confocal imaging of testis sections from 6-week-old *Amh-cre; R26^tdTomato^; Scf^GFP^* mice that were stained with anti-GFP antibody. (*n*=3 mice from 3 independent experiments). (C) Flow cytometric analysis of enzymatically dissociated testicular cells from 6-week-old *Amh-cre; R26^tdTomato^; Scf^GFP^* mice. Gray peaks represent isotype control. Black bar represents the percentage of cells that expressed the reporter. Data represent mean±s.d. (*n*=3 mice from 3 independent experiments). (D) Confocal imaging of testis sections from 6-week-old *Scf^GFP^* mice that were stained with anti-GFP and anti-DDX4 antibodies. (*n*=3 mice from 3 independent experiments). (E) Confocal imaging of testis sections from 6-week-old *Ddx4-creER; R26^tdTomato^; Scf^GFP^* mice that were stained with anti-GFP antibody. Mice were treated with tamoxifen at 4 weeks of age. (*n*=3 mice from 3 independent experiments). (F) Flow cytometric analysis of enzymatically dissociated testicular cells from 6-week-old *Ddx4-creER; R26^tdTomato^; Scf^GFP^* mice. Gray peaks represent isotype control. Black bar represents the percentage of cells that expressed the reporter. Mice were treated with tamoxifen at 4 weeks of age. Data represent mean±s.d. (*n*=3 mice from 3 independent experiments). In image panels, boxed area in the top-left image is shown at higher magnification in the surrounding panels as separate channels and merge.

We employed *Scf^GFP^* knock-in mice, in which a GFP reporter gene was inserted into the endogenous *Scf* locus (Ding et al., 2012), to validate the expression pattern of *Scf* in the testis. Confocal imaging of testis sections from 6-week-old *Scf^GFP^* mice detected GFP expression in many somatic cell types of the testis (Fig. 2A). The GFP signals showed extensive overlap with anti-Sox9 staining, a marker of Sertoli cells (Kent et al., 1996), in the seminiferous tubules, suggesting that Sertoli cells are a cellular source of SCF.

We next quantified the percentage of all SCF^+^ cells that were Sertoli cells and the percentage of all Sertoli cells that expressed SCF. *Amh-cre* mice were introduced to specifically and efficiently target Sertoli cells (Lécureuil et al., 2002). These mice were sequentially crossed with *R26^tdTomato^* mice (Madisen et al., 2010) and *Scf^GFP^* mice to generate *Amh-cre; R26^tdTomato^; Scf^GFP^* compound mutant mice. On testis sections of these mice at 6 weeks of age, Tomato and GFP signals displayed significant overlap (Fig. 2B). Flow cytometric analysis of enzymatically dissociated *Amh-cre; R26^tdTomato^; Scf^GFP^* testicular cells showed that ∼99% of all Tomato^+^ cells expressed GFP and ∼81% of all GFP^+^ cells were Tomato positive (Fig. 2C). Thus, Sertoli cells uniformly express SCF.

DDX4 is a germ cell-specific marker (Fujiwara et al., 1994). Anti-DDX4 staining on testis sections from 6-week-old *Scf^GFP^* mice showed that DDX4^+^ germ cells did not detectably express GFP (Fig. 2D). Consistent with this, Tomato and GFP signals were mutually exclusive on testis sections from 6-week-old *Ddx4-creER; R26^tdTomato^; Scf^GFP^* mice at 2 weeks after tamoxifen treatment (Fig. 2E). Flow cytometry of enzymatically dissociated testicular cells from these mice showed that few Tomato^+^ cells expressed GFP and few GFP^+^ cells were Tomato positive (Fig. 2F). Therefore, germ cells are not a source of SCF in the testis.

**Fig. 3.**
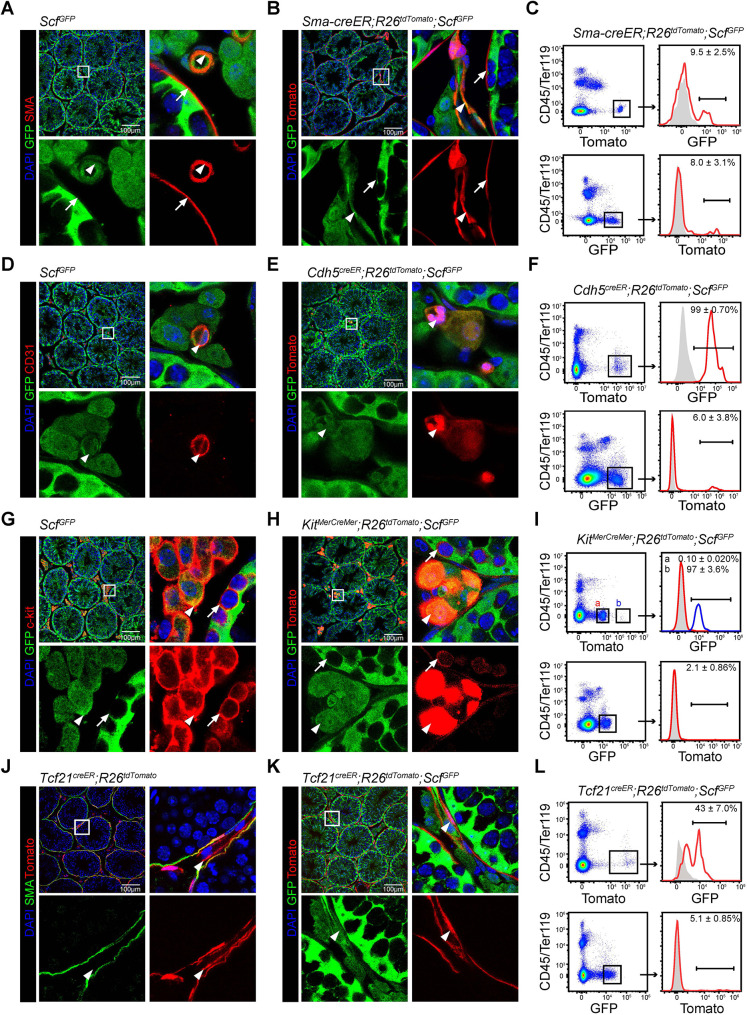
***Scf*-GFP is expressed by smooth muscle cells, endothelial cells, Leydig cells and *Tcf21-*creER^+^ stromal cells in the testicular interstitium.** (A) Confocal imaging of testis sections from 6-week-old *Scf^GFP^* mice that were stained with anti-GFP and anti-α-SMA antibodies. Arrows indicate peritubular α-SMA^+^ cells and arrowheads indicate perivascular α-SMA^+^ cells. (*n*=3 mice from 3 independent experiments). (B) Confocal imaging of testis sections from 6-week-old *Sma-creER; R26^tdTomato^; Scf^GFP^* mice that were stained with anti-GFP antibody. Mice were treated with tamoxifen at 4 weeks of age. Arrows indicated peritubular α-SMA^+^ cells and arrowheads indicated perivascular α-SMA^+^ cells. (*n*=3 mice from 3 independent experiments). (C) Flow cytometric analysis of enzymatically dissociated testicular cells from 6-week-old *Sma-creER; R26^tdTomato^; Scf^GFP^* mice. Mice were treated with tamoxifen at 4 weeks of age. (D) Confocal imaging of testis sections from 6-week-old *Scf^GFP^* mice that were stained with anti-GFP and anti-CD31 antibodies. Arrowheads indicate *Scf*-GFP^+^ endothelial cells. (*n*=3 mice from 3 independent experiments). (E) Confocal imaging of testis sections from 6-week-old *Cdh5-creER; R26^tdTomato^; Scf^GFP^* mice that were stained with anti-GFP antibody. Mice were treated with tamoxifen at 4 weeks of age. Arrowheads indicate *Scf*-GFP^+^ endothelial cells. (*n*=3 mice from 3 independent experiments). (F) Flow cytometric analysis of enzymatically dissociated testicular cells from 6-week-old *Cdh5-creER; R26^tdTomato^; Scf^GFP^* mice. Mice were treated with tamoxifen at 4 weeks of age. (G) Confocal imaging of testis sections from 6-week-old *Scf^GFP^* mice that were stained with anti-GFP and anti-c-Kit antibodies. Arrows indicate intratubular c-Kit^+^ cells and arrowheads indicate interstitial c-Kit^+^ cells. (*n*=3 mice from 3 independent experiments). (H) Confocal imaging of testis sections from 6-week-old *Kit^MerCreMer^; R26^tdTomato^; Scf^GFP^* mice that were stained with anti-GFP antibody. Mice were treated with tamoxifen at 4 weeks of age. Arrows indicate intratubular c-Kit^+^ cells and arrowheads indicate interstitial c-Kit^+^ cells. (*n*=3 mice from 3 independent experiments). (I) Flow cytometric analysis of enzymatically dissociated testicular cells from 6-week-old *Kit^MerCreMer^; R26^tdTomato^; Scf^GFP^* mice. Mice were treated with tamoxifen at 4 weeks of age. Gate ‘a’ represents Tomato^dim^ cells and gate ‘b’ represents Tomato^bright^ cells. (J) Confocal imaging of testis sections from 6-week-old *Tcf21^creER^; R26^tdTomato^* mice that were stained with anti-SMA antibody. Arrowheads indicate Tomato^+^SMA^−^ peritubular mesenchymal cells. (*n*=3 mice from 3 independent experiments). (K) Confocal imaging of testis sections from 6-week-old *Tcf21^creER^; R26^tdTomato^; Scf^GFP^* mice that were stained with anti-GFP antibody. Mice were treated with tamoxifen at 4 weeks of age. Arrowheads indicate Tomato^+^*Scf*-GFP^+^ peritubular mesenchymal cells. (*n*=3 mice from 3 independent experiments). (L) Flow cytometric analysis of enzymatically dissociated testicular cells from 6-week-old *Tcf21^creER^; R26^tdTomato^; Scf^GFP^* mice. Mice were treated with tamoxifen at 4 weeks of age. In flow cytometry graphs, gray peaks represent isotype control. Black bar represents the percentage of cells that expressed the reporter. Data represent mean±s.d. (*n*=3 mice from 3 independent experiments). In image panels, boxed area in the top-left image is shown at higher magnification in the surrounding panels as separate channels and merge.

*Scf*-GFP is expressed by smooth muscle cells, endothelial cells, Leydig cells and *Tcf21-*creER^+^ stromal cells in the testicular interstitium. α-SMA is a marker for vascular smooth muscle cells and myoid cells in the testis (Owens and Thompson, 1986; Tung and Fritz, 1989). GFP expression was detected in α-SMA^+^ perivascular cells (Fig. 3A, arrowheads), but not in α-SMA^+^ peritubular cells (Fig. 3A, arrows) on testis sections from 6-week-old *Scf^GFP^* mice. Consistent with this, GFP expression was detected in Tomato^+^ perivascular cells (Fig. 3B, arrowheads), but not in Tomato^+^ peritubular cells (Fig. 3B, arrows) on testis sections from 6-week-old *Sma-creER; R26^tdTomato^; Scf^GFP^* mice at 2 weeks after tamoxifen treatment. By flow cytometry, approximately 9.5% of all Tomato^+^ cells expressed a low level of GFP and 8.0% of all GFP^+^ cells expressed Tomato in the testis of *Sma-creER; R26^tdTomato^; Scf^GFP^* mice (Fig. 3C). Thus, consistent with the scRNA-seq data, smooth muscle cells are a source of SCF in the testis.

CD31 (Pecam1) and Cdh5 are markers for vascular endothelial cells (Lampugnani et al., 1992; Newman et al., 1990). GFP expression was routinely detected in CD31^+^ cells on testis sections from 6-week-old *Scf^GFP^* mice (Fig. 3D). Consistent with this, GFP expression was detected in Tomato^+^ cells on testis sections from 6-week-old *Cdh5-creER; R26^tdTomato^; Scf^GFP^* mice at 2 weeks after tamoxifen treatment (Fig. 3E). By flow cytometry, virtually all Tomato^+^ cells expressed GFP, but only 6.0% of all GFP^+^ cells expressed Tomato in the testis of *Cdh5-creER; R26^tdTomato^; Scf^GFP^* mice (Fig. 3F). Thus, consistent with the scRNA-seq data, endothelial cells are a source of SCF in the testis.

c-Kit is expressed by both differentiating spermatogonia and Leydig cells in the testis (Manova et al., 1990; Rossi et al., 2000). GFP expression was detected in c-Kit^+^ interstitial cells (Fig. 3G, arrowheads), but not in c-Kit^+^ intratubular cells (Fig. 3G, arrows) on testis sections from 6-week-old *Scf^GFP^* mice. Consistent with this, GFP expression was detected in Tomato^bright^ interstitial cells (Fig. 3H, arrowheads), but not in Tomato^dim^ intratubular cells (Fig. 3H, arrows) on testis sections from 6-week-old *Kit^MerCreMer^; R26^tdTomato^; Scf^GFP^* mice at 2 weeks after tamoxifen treatment. By flow cytometry, Tomato^bright^ cells uniformly expressed GFP whereas Tomato^dim^ cells rarely expressed GFP in the testis of *Kit^MerCreMer^; R26^tdTomato^; Scf^GFP^* mice (Fig. 3I, top). Only 2.1% of all GFP^+^ cells were Tomato positive in these mice (Fig. 3I, bottom). Thus, consistent with the scRNA-seq data, Leydig cells are a source of SCF in the testis.

We generated *Tcf21^creER^* mice to study the unknown cell population in scRNA-seq that expressed *Scf* (Fig. 1C,E). Tomato expression was detected in SMA^+^ myoid cells, SMA^−^ peritubular cells and perivascular cells on testis sections from 6-week-old *Tcf21^creER^; R26^tdTomato^* mice at 2 weeks after tamoxifen treatment (Fig. 3J). GFP expression was detected in Tomato^+^ peritubular cells on testis sections from 6-week-old *Tcf21^creER^; R26^tdTomato^; Scf^GFP^* mice at 2 weeks after tamoxifen treatment (Fig. 3K). By flow cytometry, approximately 43% of all Tomato^+^ cells expressed low level of GFP and 5.1% of all GFP^+^ cells expressed Tomato in these mice (Fig. 3L). Thus, consistent with the scRNA-seq data, *Tcf21-*creER^+^ stromal cells are a source of SCF in the testis.

### Spermatogenesis requires SCF from Sertoli cells

**Fig. 4.**
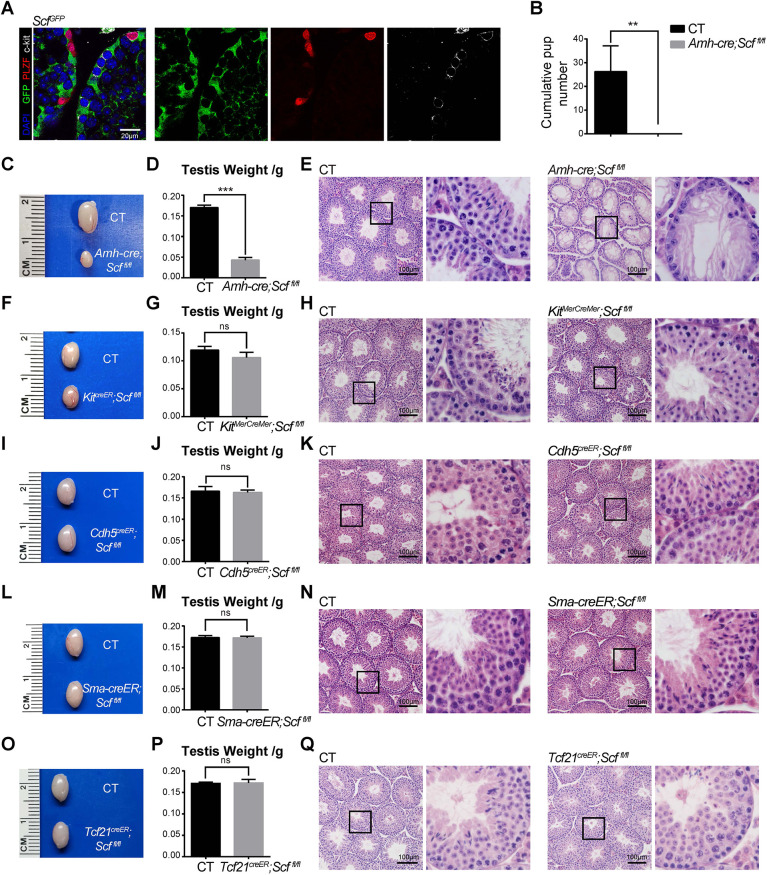
**Deletion of *Scf* from Sertoli cells, but not from Leydig, endothelial, smooth muscle cells or *Tcf21-*creER^+^ stromal cells, blocks spermatogenesis.** (A) Confocal imaging of testis sections from 6-week-old *Scf^GFP^* mice that were co-stained with anti-GFP, anti-PLZF and anti-c-Kit antibodies. (*n*=3 mice from 3 independent experiments). (B) Cumulative pup numbers of female mice co-caged with control or *Amh-cre; Scf^fl/fl^* male mice at 1:1 ratio for 6 months. **P<0.01. (C-E) Size (C), weight (D) and H&E-stained sections (E) of testes from 6-week-old *Scf^fl/fl^* and *Amh-cre; Scf^fl/fl^* mice. ***P<0.001. (F-H) Size (F), weight (G) and H&E-stained sections (H) of testes from 6-week-old *Kit^MerCreMer^; Scf^fl/fl^* and control mice at 2 weeks after tamoxifen treatment. (I-K) Size (I), weight (J) and H&E-stained sections (K) of testes from 6-week-old *Cdh5-creER; Scf^fl/fl^* and control mice at 2 weeks after tamoxifen treatment. (L-N) Size (L), weight (M) and H&E-stained sections (N) of testes from 6-week-old *Sma-creER; Scf^fl/fl^* and control mice at 2 weeks after tamoxifen treatment. (O-Q) Size (O), weight (P) and H&E-stained sections (Q) of testes from 6-week-old *Tcf21^creER^; Scf^fl/fl^* and control mice at 2 weeks after tamoxifen treatment. For graph panels, two-tailed Student's *t*-test was used to assess statistical significance (*n*=5 mice from 3 independent experiments). CT, control; ns, not significant. For image panels, boxed area in the left-hand panel is shown at higher magnification on the right.

Confocal imaging of testis sections from 6-week-old *Scf^GFP^* mice revealed that both PLZF (Zbtb16)^+^ undifferentiated spermatogonia (Buaas et al., 2004) and c-Kit^+^ differentiating spermatogonia were physically associated with the *Scf*-GFP^+^ Sertoli cells (Fig. 4A). To test directly whether Sertoli cells produce SCF to regulate spermatogenesis, we analyzed *Amh-cre; Scf^fl/fl^* male mice and their littermate controls. Western blotting confirmed that SCF protein was effectively depleted from *Amh-cre; Scf^fl/fl^* testes (Fig. S2I,J). While breeding, we found that these mice were completely sterile when mated with one wild-type female each continuously for 6 months (Fig. 4B). Testes from 6-week-old *Amh-cre; Scf^fl/fl^* mice were much smaller than controls (Fig. 4C). Their weights reduced by about three-quarters of the normal level (Fig. 4D). Hematoxylin and Eosin (H&E) staining revealed almost no spermatids or spermatocytes within the seminiferous tubules of *Amh-cre; Scf^fl/fl^* mice (Fig. 4E). Therefore, deletion of *Scf* from Sertoli cells blocks spermatogenesis.

### Spermatogenesis does not require SCF from Leydig cells, endothelial cells, smooth muscle cells or *Tcf21-*creER^+^ stromal cells

*Kit*-MerCreMer caused efficient depletion of *Scf* mRNA and SCF protein from Leydig cells (Fig. S2A,B,H-J). Four-week-old *Kit^MerCreMer^; Scf^fl/fl^* and control mice were treated with tamoxifen for three consecutive days and analyzed at 6 weeks of age. We found that the size and weight of testes from *Kit^MerCreMer^; Scf^fl/fl^* mice were not significantly different from those of control mice (Fig. 4F,G). By H&E staining, testes from *Kit^MerCreMer^; Scf^fl/fl^* mice showed normal morphology and normal production of spermatids in the seminiferous tubules (Fig. 4H). Thus, spermatogenesis does not require SCF from Leydig cells.

*Cdh5*-CreER caused efficient depletion of *Scf* mRNA and SCF protein from endothelial cells (Fig. S2C,D,H-J). Six-week-old *Cdh5-creER; Scf^fl/fl^* mice treated with tamoxifen at 4 weeks of age showed normal testis size (Fig. 4I) and weight (Fig. 4J), normal testis morphology and normal production of spermatids in the seminiferous tubules (Fig. 4K). Thus, spermatogenesis does not require SCF from endothelial cells.

*Sma*-CreER resulted in efficient depletion of *Scf* mRNA and SCF protein from smooth muscle cells (Fig. S2E,F,H-J). Six-week-old *Sma-creER; Scf^fl/fl^* mice treated with tamoxifen at 4 weeks of age showed normal testis size (Fig. 4L) and weight (Fig. 4M), normal testis morphology and normal production of spermatids in the seminiferous tubules (Fig. 4N). Thus, spermatogenesis does not require SCF from smooth muscle cells.

We used *Tcf21*-CreER to conditionally deplete *Scf* mRNA and SCF protein from peritubular mesenchymal cells (Fig. S2G,I,J). Six-week-old *Tcf21^creER^; Scf^fl/fl^* mice treated with tamoxifen at 4 weeks of age showed normal testis size (Fig. 4O) and weight (Fig. 4P), normal testis morphology and normal production of spermatids in the seminiferous tubules (Fig. 4Q). Thus, spermatogenesis does not require SCF from *Tcf21*^+^ stromal cells.

To investigate the long-term effects of *Scf* conditional deletion on spermatogenesis, we treated the conditional knockout mice and their littermates with tamoxifen once every other day from 4 to 12 weeks of age. The tamoxifen-treated testes appeared slightly smaller than untreated, but were generally normal. We did not detect obvious spermatogenic defects in *Kit^creER^; Scf^fl/fl^*, *Cdh5^creER^; Scf^fl/fl^*, *Sma-creER; Scf^fl/fl^* or *Tcf21^creER^; Scf^fl/fl^* mice, compared with their littermate controls (Fig. S3). These data are consistent with our conclusion that conditional knockout of *Scf* from Leydig, endothelial, vascular smooth muscle, or Tcf21^+^ stromal cells does not impair spermatogenesis.

### scRNA-seq defines the transcriptome of *Amh-cre; Scf^fl/fl^* testes

**Fig. 5.**
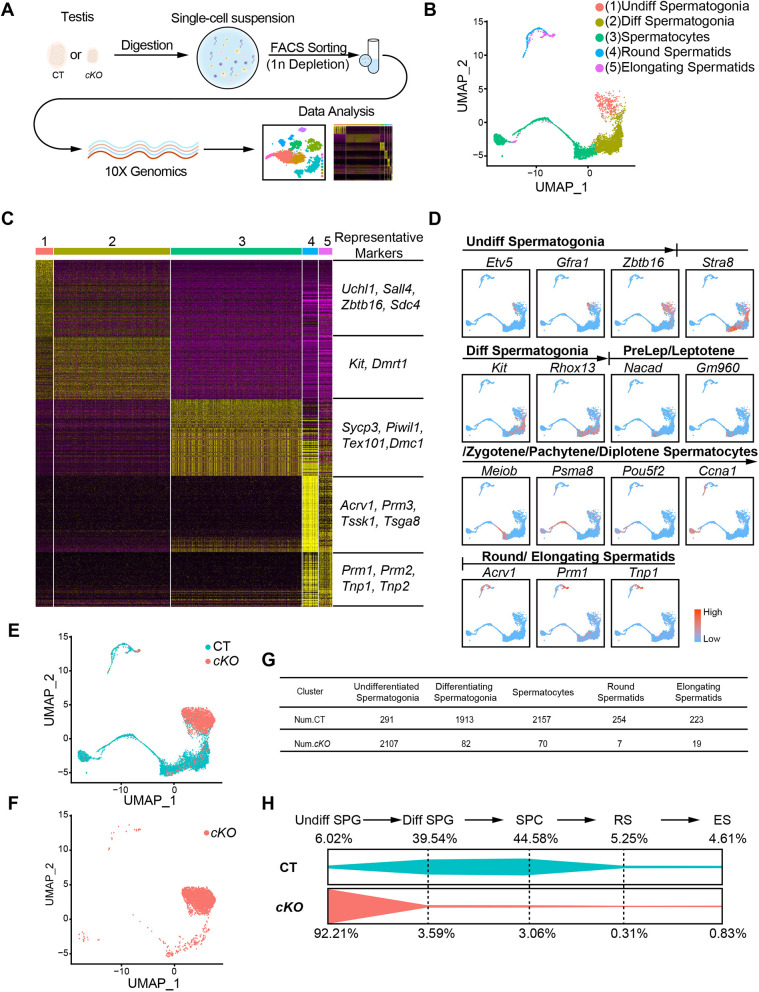
**Sertoli cell-specific deletion of *Scf* remodels the spermatogonial hierarchy.** (A) Schematic overview of the workflow for sample preparation for scRNA-seq analysis. FACS, fluorescence-activated cells sorting. (B) Clustering analysis of single-cell transcriptome data from combined testicular germ cells, in which control testicular germ cells were visualized in the UMAP space. Diff, differentiating; Undiff, undifferentiated. (C) Heat map of the top 120 differentially expressed markers among five germ cell types. (D) Gene expression patterns of distinct stage-specific markers in control testicular germ cells visualized in the UMAP space. (E) UMAP plot of combined testicular germ cells from testes of 6-week-old *Scf^fl/fl^* and *Amh-cre; Scf^fl/fl^* mice. (F) UMAP plot of testicular germ cells from testes of 6-week-old *Amh-cre; Scf^fl/fl^* mice. (G) Cell numbers of testicular cells from distinct sources in each cell cluster. (H) Summary schematic depicting percentages of undifferentiated spermatogonia (Undiff SPG), differentiating spermatogonia (Diff SPG), spermatocytes (SPC), round spermatids (RS) and elongating spermatids (ES) in testes of 6-week-old *Scf^fl/fl^* and *Amh-cre; Scf^fl/fl^* mice. cKO, conditional knockout; CT, control.

To determine the cellular mechanism by which deleting *Scf* from Sertoli cells blocks spermatogenesis, we performed scRNA-seq of whole testicular cells from three pairs of *Amh-cre; Scf^fl/fl^* and control mice. Cells were pre-sorted by flow cytometry, depleting haploid round and elongating spermatids, before being subjected to the 10x Genomics platform (Fig. 5A). Overall, 5827 cells from *Scf^fl/fl^* mice and 6837 cells from *Amh-cre; Scf^fl/fl^* mice passed standard quality control and were retained for subsequent analyses. Unsupervised clustering of all 12,664 cells projected onto UMAP analysis plot identified 20 cell types (Fig. S4A,B). Based on the expression patterns of *Ddx4* and *Tnp1*, the expression matrix of 4838 germ cells from *Scf^fl/fl^* testes and 2285 germ cells from *Amh-cre; Scf^fl/fl^* testes was extracted for further analyses (Fig. S4C-E).

Re-clustering analysis in UMAP space identified five major germ cell populations in control testicular cells (Fig. 5B). Based on the expression patterns of differentially expressed genes (DEGs; Fig. 5C) and stage-specific marker genes (Chen et al., 2018) (Fig. 5D), we defined these five cell populations as undifferentiated spermatogonia, differentiating spermatogonia, spermatocytes, round spermatids and elongating spermatids, respectively (Fig. 5B,D). Compared with controls, testes from *Amh-cre; Scf^fl/fl^* mice showed a depletion of differentiating spermatogonia (39.54% versus 3.59%) as well as downstream spermatocytes (44.58% versus 3.06%) and spermatids (5.25% versus 0.31%), but an enrichment of undifferentiated spermatogonia (6.02% versus 92.21%; Fig. 5E-H). These data suggest that deletion of *Scf* from Sertoli cells blocks spermatogenesis by depleting differentiating spermatogonia.

**Fig. 6.**
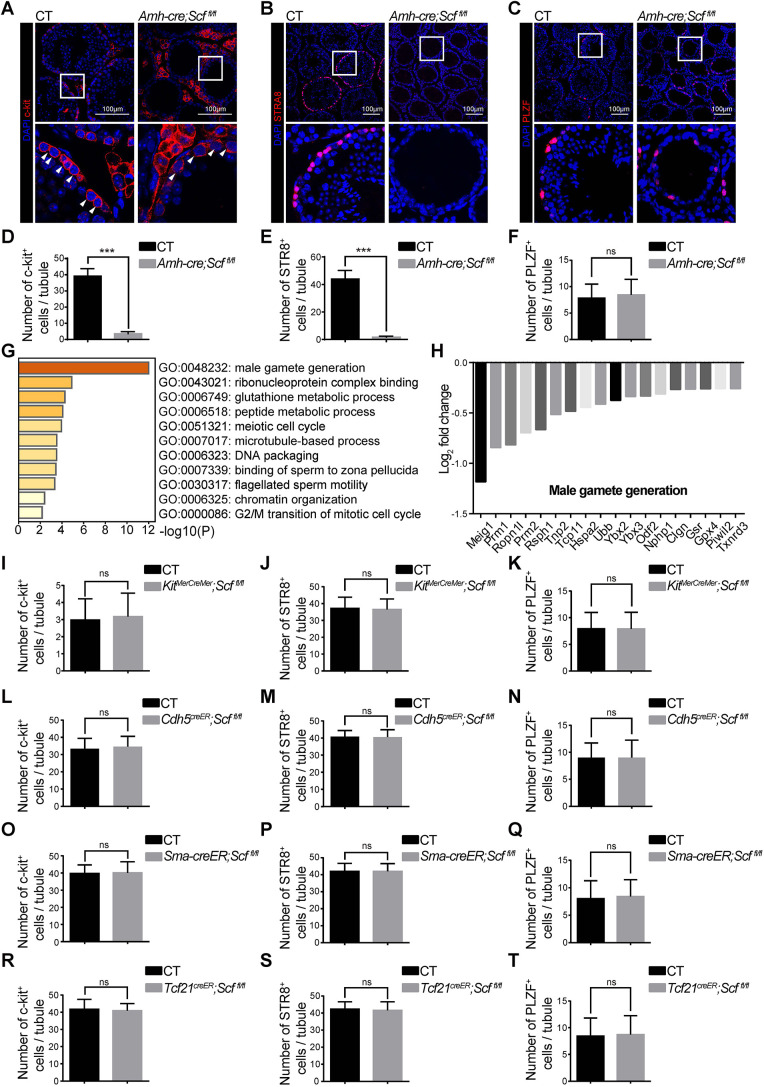
**Deletion of *Scf* from Sertoli cells depletes differentiating spermatogonia and blocks their differentiation.** (A-F) Confocal imaging of testis sections from 6-week-old old *Amh-cre; Scf^fl/fl^* and control mice that were stained with anti-c-Kit (A), anti-STRA8 (B) and anti-PLZF antibody (C), respectively. The numbers of c-Kit^+^ (D), STRA8^+^ (E) and PLZF^+^ spermatogonia (F) per tubule were quantified. Arrowheads indicate c-Kit^+^ spermatogonia. Lower panels show higher magnifications of the boxed areas above. Two-tailed Student's *t*-tests were used to assess statistical significance. ns, not significant; ***P<0.001 (*n*=5 mice from 3 independent experiments). (G,H) GO analysis of downregulated DEGs in *Amh-cre; Scf^fl/fl^* differentiating spermatogonia (G) and relative expression levels of DEGs which are enriched in the male gamete generation (H). (I-K) Quantification of c-Kit^+^ (I), STRA8^+^ (J) and PLZF^+^ spermatogonia (K) per tubule of 6-week-old *Kit^MerCreMer^; Scf^fl/fl^* and control mice at 2 weeks after tamoxifen treatment. (L-N) Quantification of c-Kit^+^ (L), STRA8^+^ (M) and PLZF^+^ spermatogonia (N) per tubule of 6-week-old *Cdh5-creER; Scf^fl/fl^* and control mice at 2 weeks after tamoxifen treatment. (O-Q) Quantification of c-Kit^+^ (O), STRA8^+^ (P) and PLZF^+^ spermatogonia (Q) per tubule of 6-week-old *Sma-creER; Scf^fl/fl^* and control mice at 2 weeks after tamoxifen treatment. (R-T) Quantification of c-Kit^+^ (R), STRA8^+^ (S) and PLZF^+^ spermatogonia (T) per tubule of 6-week-old *Tcf21^creER^; Scf^fl/fl^* and control mice at 2 weeks after tamoxifen treatment. In I-T, two-tailed Student's *t*-tests were used to assess statistical significance. ns, not significant (*n*=5 mice from 3 independent experiments). CT, control.

To validate the scRNA-seq results, we performed immunostaining of testis sections using stage-specific markers. We found that *Amh-cre; Scf^fl/fl^* mice had significantly fewer c-Kit^+^ differentiating spermatogonia (Fig. 6A,D) and STRA8^+^ premeiotic spermatogonia (Fig. 6B,E) in the testis than *Scf^fl/fl^* mice. In contrast, the number of PLZF^+^ or Gfrα1^+^ undifferentiated spermatogonia in the testis was comparable between *Scf^fl/fl^* and *Amh-cre; Scf^fl/fl^* mice (Fig. 6C,F; Fig. S5A,B). Thus, deletion of *Scf* from Sertoli cells depletes differentiating spermatogonia and their downstream lineages without affecting undifferentiated spermatogonia.

### SCF from Sertoli cells regulates spermatogonial differentiation and mitotic cell cycle

To understand better the molecular mechanism by which deleting *Scf* from Sertoli cells blocks spermatogenesis, we analyzed the DEGs in the differentiating spermatogonia population (Fig. 5B,E) in *Scf^fl/fl^* and *Amh-cre; Scf^fl/fl^* mice. Gene ontology (GO) analysis identified 88 downregulated genes and 51 upregulated genes. Notably, genes involved in male gamete generation appeared to be the most abundant downregulated gene cluster in *Amh-cre; Scf^fl/fl^* mice (Fig. 6G,H). The expression of many genes involved in the progression of meiosis, such as *Meig1*, *Rsph1*, *Hspa2*, *Ubb*, *Clgn* and *Piwil2*, was significantly downregulated in the differentiating spermatogonia from *Amh-cre; Scf^fl/fl^* mice (Fig. 6G,H), indicating a defect in meiosis entry. The expression of genes that involved in the regulation of G2/M transition of mitotic cell cycle was also significantly downregulated in differentiating spermatogonia from *Amh-cre; Scf^fl/fl^* mice (Fig. 6G; Fig. S5C), indicating that exhaustion of c-Kit^+^ spermatogonia may result from G2/M cell cycle arrest. As the phenotype of *Amh-cre; Scf^fl/fl^* testes was similar to testes from previously reported vitamin A-deficient mice (van Pelt and de Rooij, 1990), we also investigated whether spermatogenesis disruption in *Amh-cre; Scf^fl/fl^* mice was mediated by retinoic acid (RA) signaling (Fig. S5D). However, the expression patterns of RA regulators were not obviously different in differentiated or differentiating spermatogonia from *Amh-cre; Scf^fl/fl^* and control mice (Fig. S5D).

In contrast, testes from *Kit^MerCreMer^; Scf^fl/fl^* (Fig. 6I-K), *Cdh5-creER; Scf^fl/fl^* (Fig. 6L-N), *Sma-creER; Scf^fl/fl^* (Fig. 6O-Q) and *Tcf21^creER^; Scf^fl/fl^* mice (Fig. 6R-T) all had normal numbers of c-Kit^+^, STRA8^+^ and PLZF^+^ spermatogonia in the testes, supporting the notion that deletion of *Scf* from Leydig cells, endothelial cells, smooth muscle cells or *Tcf21-*creER^+^ stromal cells does not affect spermatogenesis.

To investigate whether these differences resulted from the timing of the *Scf* conditional knockout, we introduced a Sertoli cell-specific inducible cre mouse line, *Sox9^creER^*, to knock out *Scf* in Sertoli cells from 4-week-old mice. *Sox9-creER* could specifically and efficiently target Sox9^+^ Sertoli cells in 6-week-old mice administered tamoxifen at 4 weeks of age (Fig. S6A). Similar to *Amh-cre; Scf^fl/fl^* mice, testes from 6-week-old *Sox9^creER^; Scf^fl/fl^* mice given tamoxifen at 4 weeks of age were smaller and lighter than control and showed disrupted spermatogenesis (Fig. S6B-D). *Sox9^creER^; Scf^fl/fl^* mice had significantly fewer c-Kit^+^ differentiating spermatogonia (Fig. S6E,I), STRA8^+^ premeiotic spermatogonia (Fig. S6F,J) and DDX4^+^ germ cells (Fig. S6G,K), but comparable PLZF^+^ undifferentiated spermatogonia (Fig. S6H,L), in the testis than control mice. We also treated the *Sox9^creER^; Scf^fl/fl^* mice and their littermates with tamoxifen once every other day from 4 to 12 weeks of age. The phenotypes of 12-week-old *Sox9^creER^; Scf^fl/fl^* mice receiving long-term tamoxifen were more severe and spermatogenesis was completely blocked (Fig. S7), which was consistent with observations in *Amh-cre; Scf^fl/fl^* mice (Fig. 4). Thus, SCF from Sertoli cells regulates the differentiation and mitotic cell cycle of spermatogonia during homeostasis.

### Conditional overexpression of *Scf* in Sertoli cells, but not endothelial cells, increases spermatogonial differentiation

**Fig. 7.**
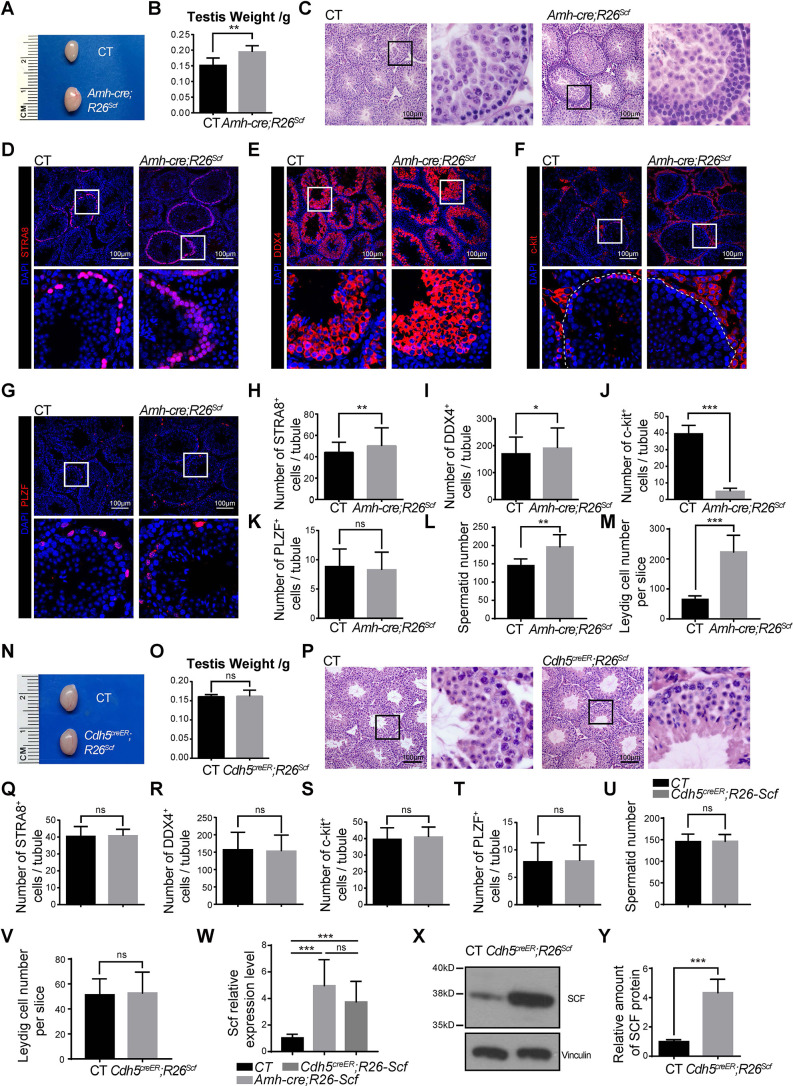
**Overexpression of *Scf* in Sertoli cells, but not endothelial cells, increases spermatogenesis.** (A-C) Size (A), weight (B) and H&E-stained sections (C) of testes from 6-week-old *Amh-cre; R26^Scf^* and control mice. **P<0.01. (D-M) Confocal imaging of testis sections from 6-week-old *Amh-cre; R26^Scf^* and control mice that were stained with anti-STRA8 (D), anti-DDX4 (E), anti-c-Kit (F) and anti-PLZF (G) antibodies, respectively. The numbers of STRA8^+^ (H), DDX4^+^ (I), c-Kit^+^ (J) and PLZF^+^ (K) germ cells and spermatids (L) per tubule and c-Kit^+^ Leydig cells per slice (M) were quantified. Dashed lines indicate an outline of seminiferous tubules. *P<0.05, **P<0.01, ***P<0.001. (N-P) Size (N), weight (O) and H&E-stained sections (P) of testes from 6-week-old *Cdh5-creER; R26^Scf^* and control mice at 2 weeks after tamoxifen treatment. (Q-V) Quantification of the numbers of STRA8^+^ (Q), DDX4^+^ (R), c-Kit^+^ (S), PLZF^+^ (T) germ cells and spermatids (U) per tubule and c-Kit^+^ Leydig cells per slice (V) of 6-week-old *Cdh5-creER; R26^Scf^* and control mice at 2 weeks after tamoxifen treatment. (W) Relative expression of *Scf* transcripts in testes from *Cdh5^creER^; R26^Scf^*, *Amh-cre; R26^Scf^* and control mice. ***P<0.001 (two-tailed Student's *t*-test) (*n*=3 mice from 3 independent experiments). (X) Western blot analysis of SCF expression in purified CD31^+^ endothelial cells from *Cdh5^creER^; R26-Scf* and control mice. (Y) Quantification of the western blots shown in X. Expression levels of SCF protein were normalized to vinculin. ***P<0.001 (two-tailed Student's *t*-test) (*n*=3). In image panels, boxed areas are shown at higher magnification to the right or below. In graph panels (except W and Y), two-tailed Student's *t*-test was used to assess statistical significance (*n*=5 mice from 3 independent experiments). CT, control; ns, not significant.

We next investigated whether elevated SCF expression in specific cell types is sufficient to promote spermatogenesis. An *R26^Scf^* mouse was designed by targeted insertion of a construct containing the ubiquitous CAG promoter, followed by a floxed-Stop cassette-controlled *Scf* gene, into the *Rosa26* locus (Fig. S8A). Cre-mediated recombination is able to overexpress *Scf* in a cell type-specific manner. We first generated *Amh-cre; R26^Scf^* mice to overexpress *Scf* in specifically Sertoli cells. Testes from 6-week-old *Amh-cre; R26^Scf^* mice were significantly larger and heavier than those from control mice (Fig. 7A,B). By H&E staining, we detected a marked increase of germ cells in the testis from *Amh-cre; R26^Scf^* mice (Fig. 7C). Consistent with this, we observed a significant increase in the numbers of STRA8^+^ cells (Fig. 7D,H), DDX4^+^ cells (Fig. 7E,I) and spermatids (Fig. 7L) in the testis of *Amh-cre; R26^Scf^* mice compared with controls, which is likely responsible for the increase of testis weight and size. The increased number of STRA8^+^ differentiated spermatogonia was accompanied by an exhaustion of c-Kit^+^ spermatogonia from *Amh-cre; R26^Scf^* mice (Fig. 7F,J), although the number of PLZF^+^ cells in the testis from these mice did not differ from controls (Fig. 7G,K). This likely resulted from the accelerated differentiation of c-Kit^+^ spermatogonia to STRA8^+^ pre-meiotic cells rather than an effect on survival of c-Kit^+^ spermatogonia, as the downstream STRA8^+^ cells were increased rather than depleted or decreased. We also observed elevated spermatogenesis in 6-week-old testes from *Sox9^creER^; R26^Scf^* mice that had received tamoxifen at 4 weeks of age, albeit to a lesser extent than those from *Amh-cre; R26^Scf^* mice (Fig. S9A-M). Therefore, SCF overexpression in Sertoli cells augments the differentiation of differentiating spermatogonia.

In contrast, testes from tamoxifen treated 6-week-old *Cdh5^creER^; R26^Scf^* mice had normal size and weight (Fig. 7N,O), normal morphology (Fig. 7P) and normal numbers of STRA8^+^, DDX4^+^, c-Kit^+^ and PLZF^+^ cells and spermatids (Fig. 7Q-U; Fig. S8B-E). Notably, the overall expression level of *Scf* in *Cdh5^creER^; R26^Scf^* testes was significantly higher than in control testes but comparable to *Amh-cre; R26^Scf^* testes (Fig. 7W). Western blot analysis showed elevated SCF protein expression level in *Cdh5^creER^; R26^Scf^* endothelial cells compared with control cells (Fig. 7X,Y). Spermatogenesis remained normal in *Cdh5^creER^; R26^Scf^* mice even when we analyzed them at 8 weeks after tamoxifen treatment (Fig. S8F-L). Thus, these results suggest that overexpression of *Scf* in endothelial cells is insufficient to promote spermatogenesis.

We also observed an increase of c-Kit^+^ Leydig cells in *Amh-cre; R26^Scf^* testes, but not in *Cdh5^creER^; R26^Scf^* testes (Fig. 7M,V), raising the possibility of an indirect effect of overexpression of *Scf* on spermatogenesis through Leydig cell-mediated regulation of germ cell development. In view of a previous *in vitro* study of *Scf* function on spermatogonia (Pellegrini et al., 2008), it is more likely that the increased spermatogenesis results from a direct regulation of *Scf* on c-Kit^+^ spermatogonia differentiation.

## DISCUSSION

Recent studies have employed scRNA-seq to map all the germ cell and somatic cell types in mouse testis (Green et al., 2018; Hermann et al., 2018). In this study, we used scRNA-seq to systematically map the expression patterns of growth factors that regulate spermatogenesis in the testis. Although our scRNA-seq study failed to detect *Gdnf* in Sertoli cells, it detected many other known Sertoli cell-secreted factors, such as *Clu*, *Cst9*, *Cst12*, *Ptgds*, *Inha*, *Lrp8*, *Eppin* and *Serpina5*, in Sertoli cells (Fig. S10B). In fact, when we re-analyzed previous published scRNA-seq studies that included Sertoli cells (Green et al., 2018; Grive et al., 2019; Han et al., 2018; Jung et al., 2019; Wang et al., 2019), none of them detected *Gdnf* (Fig. S10A). Therefore, this is likely a general limitation of the current scRNA-seq technique in detecting certain genes. To test this directly, we performed scRNA-seq and quantitative real-time PCR (qRT-PCR) on the same batch of purified Sertoli cells or myoid cells, comparing the two methods with respect to the detection of *Gdnf* expression levels. We found that qRT-PCR, but not scRNA-seq, detected robust expression of *Gdnf* in Sertoli cells and myoid cells (Fig. S10C-G). The false-negative results from scRNA-seq emphasize the importance of further phenotypic and functional validation of certain genes, as we have done here.

The spermatogenic niche is usually defined as a place where SSCs reside and are nursed with growth factors from neighboring cells. Sertoli cells are known to be a niche for SSCs by secreting growth factors, such as GDNF (Meng et al., 2000). We showed that conditional deletion of *Scf* from Sertoli cells led to depletion of differentiating spermatogonia but not SSCs (Fig. 6A-F). Conditional overexpression of Scf from Sertoli cells also depleted differentiating spermatogonia (Fig. 7F,J). Our data suggest that progenitors downstream of SSCs also require a niche, as do SSCs.

SCF/c-Kit signaling has been proposed to promote proliferation of and inhibit apoptosis of differentiating spermatogonia (Feng et al., 2000; Ohta et al., 2000; Yan et al., 2000). Our work confirmed this notion by showing that conditional deletion of *Scf* from Sertoli cells depleted most c-kit^+^ spermatogonia (Figs 5E-H and 6A,D; Fig. S6E,I). Furthermore, we found that *Scf* deletion also led to depletion of most germ cells downstream of c-Kit^+^ spermatogonia (Figs 5E-H and 6B,E; Fig. S6F,J). This is likely caused by the reduced number of c-Kit^+^ progenitors. Notably, many genes involved in meiosis entry were transcriptionally inhibited in these cells (Fig. 6G,H), suggesting that the differentiation defect of *Scf*-deficient spermatogonia might be another mechanism. Consistent with this, overexpression of SCF in Sertoli cells exhausted c-Kit^+^ spermatogonia (Fig. 7F,J), but increased the number of their downstream lineages (Fig. 7D,E,H,I,L). In this case, SCF confers an epistatic effect on the differentiation of c-Kit^+^ spermatogonia. Thus, we conclude that SCF/c-Kit signaling plays dual roles in the maintenance and differentiation of differentiating spermatogonia.

## MATERIALS AND METHODS

### Mice

Mice used in this study were maintained in C57BL/6 background, including *Sma-creER* (Sheikh et al., 2015), *Ddx4-creER* (John et al., 2008), *Amh-cre* (Lécureuil et al., 2002), *Kit^MerCreMer^* (Van Berlo et al., 2014), *R26^tdTomato^* (Madisen et al., 2010), *Scf^fl/fl^* (Ding et al., 2012), *Sox9^creER^* (He et al., 2017) and *Scf^GFP^* (Ding et al., 2012)*. Cdh5^creER^* mice were constructed by knocking *creER* into the ATG site of the *Cdh5* gene. *R26^Scf^* mice were constructed by knocking *CAG-loxP-Stop-loxP-Scf* into the *Rosa26* locus. *Tcf21^creER^* mice was constructed by knocking *creER* into the endogenous *Tcf21* locus. Both *Tcf21^creER^* and *Cdh5^creER^* were generated by a knock-in strategy, whereby the ATG site of *Tcf21* or *Cdh5* gene was replaced by a CreER construct by Beijing Biocytogen Co. For induction of CreER activity, 4-week-old mice were treated with 1 mg tamoxifen (Sigma-Aldrich, T5648-5G) daily for 3 days. For long-term induction of CreER activity, mice were treated with 1 mg tamoxifen once every 2 days from 4 to 12 weeks of age. All procedures were approved by the Institutional Animal Care and Use Committees of SIBCB.

### Immunofluorescence analysis

Testes were fixed in 4% paraformaldehyde overnight at 4°C. After dehydration in 30% sucrose for another day, samples were embedded with OCT (Thermo Fisher Scientific, NEG-50-6502) and then sectioned (10 μm thickness) using a cryostat (Leica CM3050S). Sections were incubated with primary antibodies overnight at 4°C and then washed three times in PBS. After a 2 h incubation with secondary antibodies at room temperature, sections were washed in PBS and mounted on slides with Prolong Gold Antifade Reagent (Invitrogen). Antibodies were diluted in PBS buffer containing 10% donkey serum and 0.1% Triton X-100. Images were obtained by Leica TCS SP8 WLL or Leica TCS SP8 STED confocal microscopy.

Primary antibodies used were: anti-DDX4 (1:400; Abcam, ab13840), anti-SMA (1:250; Abcam, ab5694), anti-CD31 (1:250; R&D Systems, AF3628), anti-c-Kit (1:100; R&D Systems, AF1356), anti-GFP (1:400; Aves Labs, GFP-1020), anti-PLZF (1:200; Santa Cruz Biotechnology, sc-28319), anti-STRA8 (1:200; Abcam, ab49602), anti-SOX9 (1:200; Millipore, AB5535), anti-GFRα-1 (1:100; R&D Systems, AF560).

Secondary antibodies used were: donkey anti-rabbit Alexa Fluor 488 (1:500; Invitrogen, A21206), donkey anti-rabbit Alexa Fluor 488 (1:500; Invitrogen, A31572), donkey anti-rabbit Alexa Fluor 647 (1:500; Invitrogen, A31573), donkey anti-goat Alexa Fluor 488 (1:500; Invitrogen, A11055), donkey anti-goat Alexa Fluor 488 (1:500; Invitrogen, A21432), donkey anti-goat Alexa Fluor 647 (1:500; Invitrogen, A21447), donkey anti-chicken Alexa Fluor 488 (1:800; Jackson ImmunoResearch, 703-545-155).

### Histological analysis

Testes were fixed in Bouin's buffer overnight at room temperature, dehydrated through an ethanol series (70%, 80%, 95%, 100%), embedded in paraffin and then sectioned (10 µm thickness) using a Leica manual rotary microtome (Leica Biosystems). After dewaxing and hydration, the sections were stained with H&E and mounted on slides for imaging by BX51 (Olympus) microscopy.

### Flow cytometry

After removal of the tunica albuginea, testes were placed in 10 ml HBSS containing 100 μl 100 mg/ml Collagenase I (Worthington Biochemical, LS004197) and 100 μl 10 mg/ml DNase I (Sigma-Aldrich, 11284932001). Following incubation for 10 min in a shaking bath at 32°C, separated tubules were placed on ice for 5 min to allow for sedimentation by gravity. The supernatant was collected, and pellets were further digested for another 8 min at 32°C in HBSS containing 0.25% trypsin (Gibco, 25200072) and 0.1 ml DNase I (10 mg/ml); 1 ml of fetal bovine serum was added to inactivate trypsin. Cell suspensions were combined and filtered through a nylon mesh with a 70 µm pore size. After centrifugation at 500 ***g*** for 5 min at 4°C, cells were resuspended in 200 μl HBSS buffer, and incubated with CD45-APC (1:200; BioLegend, 103112) and Ter119-APC (1:200; BioLegend, 116212) on ice for 30 min. DAPI (1:1000; Invitrogen) was added to exclude dead cells. Flow cytometry was performed on a Cytoflex LX (Beckman) or Aria SORP (BD Biosciences) flow cytometer.

1n-depletion experiments were performed as previously described (Gaysinskaya et al., 2014). Briefly, testicular cells from *Amh-cre; Scf^fl/f^*^l^ or control mice were stained with Hoechst 33342 and propidium iodide (Thermo Fisher Scientific), and then sorted by flow cytometry to remove haploid germ cells. Flow cytometry was performed on an Aria SORP flow cytometer (BD Biosciences).

### scRNA-seq library preparation and data analysis

A single-cell library was generated using the Chromium Single Cell 3′ Library Kit v2. Sequencing of the library was performed on the Illumina HiSeq X Ten PE150 platform. Aligned reads and gene-barcode matrices were then generated from FASTQ files including Read 1, Read 2 and i7 index using the Cell Ranger (v.2.1.1) processing pipeline.

Further analyses were performed with R package Seurat (v3.1). Genes expressed in fewer than three cells in a sample were excluded. Threshold of unique counts over 3500 or less than 200 was set to filter cell doublets. Low-quality cells that had >10% mitochondrial counts were filtered. ‘LogNormalize’ method was conducted for normalization for each cell based on the total expression. The ‘FindVariableGenes’ function with default set was performed to detect highly variable genes across the single cells. The ‘FindClusters’ function was used to cluster cells into different groups. The key parameters of the ‘FindClusters’ function were set as described below.

For the data of enriched testicular somatic cells, standard data analysis was performed with R package Seurat (v3.1).The data from sorted testicular cells for the 1n-depletion experiment were also integrated to obtain more somatic cells. After principal component analysis (PCA), the selected top 15 principal components (PCs) were used for dimensionality reduction. Then, the ‘FindClusters’ function (resolution=0.3) was used for clustering of all testicular cells. Based on the expression patterns of known germ cell marker gene *Ddx4* and the elongating spermatid marker gene *Tnp1*, the gene expression matrix of testicular somatic cells was extracted for subsequent analysis. After PCA, the selected top ten PCs were used for dimensionality reduction. The ‘FindClusters’ function (resolution=0.08) was used for clustering of all somatic cells. The ‘RuntSNE’ function with default setting was then applied to visualize all somatic cells in the t-SNE plots. The top 100 markers (or all markers if fewer than 100) of each cluster were identified using the ‘FindMarkers’ function and applied to plot a heatmap for marker genes.

For comparative analyses, we first performed integrated analyses on the scRNA-seq data from control and conditional knockout testes with R package Seurat (v3.1), which promotes the identification of common cell types and enables comparative analyses (Stuart et al., 2019). The highly variably genes for all cells were selected as described above to perform PCA. The selected top 20 PCs were then used for dimensionality reduction (the number of components was determined based on standard deviations of the PCs in a plateau region of an ‘elbow plot’). Subsequently, the ‘FindClusters’ function (resolution=0.5) was used for clustering of all testicular cells. By use of the ‘RunUMAP’ function, 19 initially identified cell clusters were visualized in the UMAP plot.

According to the expression pattern of the known germ cell marker gene *Ddx4* and the elongating spermatids marker gene *Tnp1*, testicular cells were divided into somatic cell and germ cell clusters, then the expression matrix of cells in the germ cell clusters was extracted for subsequent analysis. The highly variable genes for the germ cells were used to perform PCA and then the top 25 PCs were selected for dimensionality reduction. The ‘FindClusters’ function (resolution=0.3) was used for clustering of all germ cells. The ‘RunUMAP’ function with default setting was then applied to visualize all germ cells in the UMAP plots. Eight cell clusters were initially identified, and then merged according to the expression patterns of canonical marker genes (four neighboring clusters were assigned as the spermatocyte group). The top 120 markers (or all markers if fewer than 120) of each cluster were identified using the ‘FindMarkers’ function and applied to plot a heatmap for maker genes. GO analysis was performed using Metascape (Zhou et al., 2019).

### scRNA-seq by Smart-seq2

Single Tomato^+^ cells from testes of *Amh-cre; R26^tdTomato^* and *Sma-creER; R26^tdTomato^* mice were sorted into 96-well plates (Bio-Rad, HSP9601) on a 4^○^C holder and subjected to a full-length scRNA-seq protocol (Smart-seq2). In brief, cells were lysed in single-cell RNA lysis buffer containing 0.2% Triton X-100, then subjected to reverse transcription with SuperScript II reverse transcriptase (Invitrogen) and whole transcription amplification with KAPA HiFi HotStart ReadyMix (2×; KAPA Biosystems). PCR products were purified with AMPure XP beads (Agencourt) and quantified with a Qubit dsDNA HS Assay Kit (Thermo Fisher). cDNA libraries were constructed with a Nextera XT DNA Library Preparation Kit (Illumina) and were sequenced on an Illumina HiSeq X Ten instrument in 150-bp paired-end-read mode by Novogene. The sequencing quality of all raw sequencing data was evaluated by FASTQC. Reads were mapped to the mouse GRCm38 genome assemblies by STAR with default settings. Uniquely aligned reads were counted by RSEM. Transcripts per million (TPM) gene expression values were used for subsequent analysis in Seurat.

### Quantitative real-time PCR

Cells were sorted directly into TRIzol (Life Technologies). RNA was reverse-transcribed using SuperScript III Reverse Transcriptase (Life Technologies). Quantitative real-time PCR was performed using SYBR green on a LightCycler 96 system (Roche). Primers were: β-actin: 5′-CGTCGACAACGGCTCCGGCATG-3′ and 5′-GGGCCTCGTCACCCACATAGGAG-3′; *Scf*: 5′-TTGTTACCTTCGCACAGTGG-3′ and 5′-AATTCAGTGCAGGGTTCACA-3′; *Gdnf*: 5′-GCCACTTGGAGTTAATGTCC-3′ and 5′-CTTCGAGAAGCCTCTTACCG-3′.

### Western blots

For *Amh-cre; Scf^fl/fl^* and control mice, whole testicular cells were prepared by an enzymatic digestion and centrifugation protocol that enriches Sertoli cells (Bernardino et al., 2018). For *Kit^creER^; Scf^fl/fl^* and *Cdh5^creER^; Scf^fl/fl^* mice, biotin anti-c-Kit microbeads (BioLegend, 105803, MACS, 130-090-485) and anti-CD31 microbeads (Miltenyi Biotec, 130-097-418) were used to enrich c-Kit^+^ and Cdh5^+^ cells, respectively. For *Sma^creER^; R26^tdTomato^; Scf^fl/fl^*, *Tcf21^creER^; R26^tdTomato^; Scf^fl/fl^*, and their control mice, *SMA*^+^ and *Tcf21*^+^ stromal cells were purified by fluorescence-activated cell sorting based on Tomato expression (purity >90%). For *Cdh5^creER^; R26^Scf^* and control mice, whole testicular cells were prepared without enriching any specific cell types. Proteins (from no fewer than 200,000 cells) were extracted using the Minute™ Total Protein Extraction Kit (Invent Biotechnologies, SD-001), separated by 10% SDS-PAGE gel, and blotted with anti-SCF antibody (Abcam, ab64677). Images were obtained with a MiniChemi 610 chemiluminescent imager (Sage Creation, Beijing, China). Quantification analyses of the western blots were performed using ImageJ.

### Statistical analysis

Statistical values were obtained from at least three independent samples in each experiment. Unless otherwise specified, data were analyzed using GraphPad Prism7 following standard two-tailed Student's *t*-tests, and data are given as mean±s.d. *P*<0.05 was considered as a significant difference.

## Supplementary Material

10.1242/develop.200706_sup1 Supplementary information
