# Supplementary material for: Sertoli cells are the source of stem cell factor for spermatogenesis
Source: Development. 2023 Mar 20;150(6):dev200706. doi: 10.1242/dev.200706 (PMC10112922; doi:10.1242/dev.200706)
Supplement: Supplementary information [file develop-150-200706-s1.pdf]

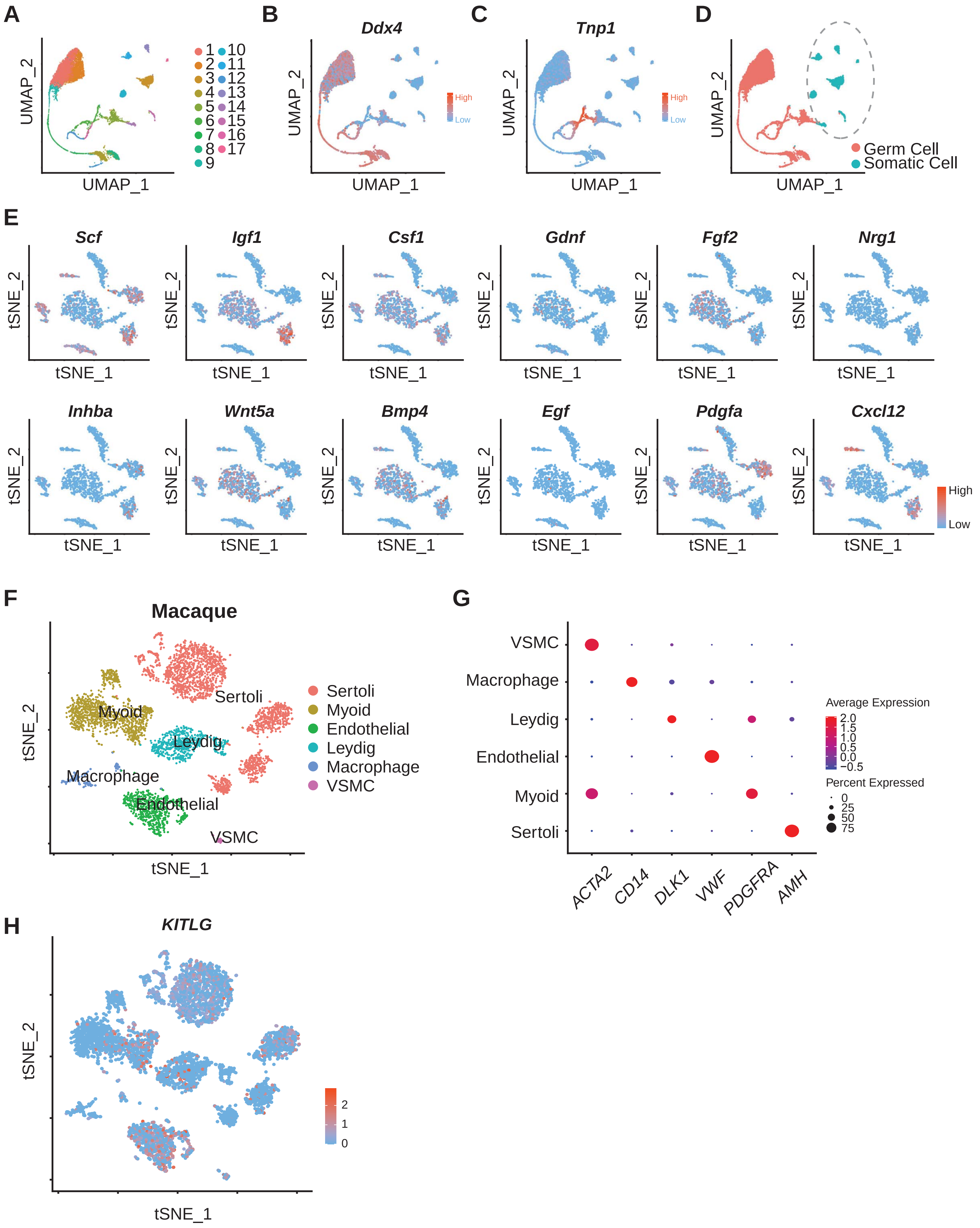
A
B
C
D
E
F
G
H
UMAP_2
UMAP_1
Ddx4
Tnp1
High
Low
Germ Cell
Somatic Cell
Scf
Igf1
Csf1
Gdnf
Fgf2
Nrg1
Inhba
Wnt5a
Bmp4
Egf
Pdgfa
Cxcl12
tSNE_2
tSNE_1
Macaque
Sertoli
Myoid
Endothelial
Leydig
Macrophage
VSMC
Average Expression
Percent Expressed
ACTA2
CD14
DLK1
VWF
PDGFRA
AMH
KITLG

Fig. S1. Clustering analysis of single cell RNA-sequencing data from testicular cells.

(A) UMAP and clustering analysis of single-cell transcriptome data from total 11209 testicular cells.

(B, C) UMAP plots showing expression patterns of germ cell specific marker gene *Ddx4* (B) and elongating spermatid marker gene *Tnp1* (C).

(D) UMAP plot showing the assigned germ cell cluster and somatic cell cluster. Gene expression matrix for the gated cells was extracted for further analyses.

(E) Gene expression patterns of known growth factors in testicular somatic cells visualized on t-SNE spaces.

(F) t-SNE and clustering analysis of single-cell transcriptome data from macaque testicular somatic cells (1).

(G) Dot-plot showing the expression patterns of distinct cell specific marker genes in six somatic cell clusters.

(H) Gene expression patterns of SCF in testicular somatic cells visualized on t-SNE spaces.

Reference:

1. Lau X, Munusamy P, Ng MJ, & Sangrithi M (2020) Single-Cell RNA Sequencing of the Cynomolgus Macaque Testis Reveals Conserved Transcriptional Profiles during Mammalian Spermatogenesis. Dev Cell 54(4):548-566 e547.

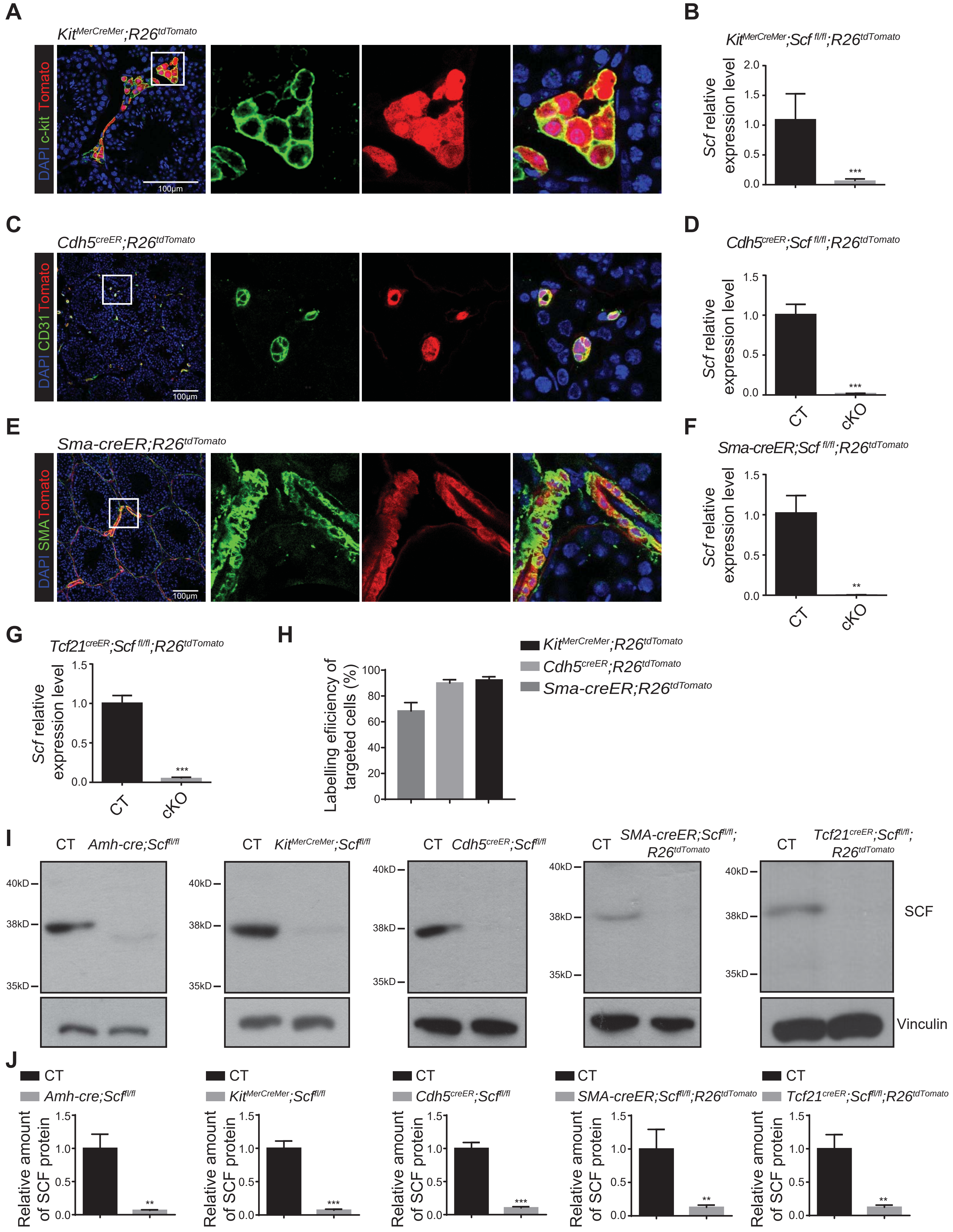

Development • Supplementary information

Fig. S2. Efficient deletion of *Scf* in targeted cell types.

(A) Confocal imaging of testis sections from 6-week-old *Kit*^{MerCreMer}; *R26*^{tdTomato} mice that were stained with anti-c-kit antibody. Mice were treated with tamoxifen at 4 weeks of age. (n=3 mice from 3 independent experiments)

(B) Relative expression of *Scf* transcripts in sorted Tomato^{+} cells from *Kit*^{MerCreMer}; *Scf*$^{fl/fl}$; *R26*^{tdTomato} (*cKO*) and *Kit*^{MerCreMer}; *R26*^{tdTomato} mice (CT) treated with tamoxifen at 4 weeks of age. ***p < 0.001 (n=3 mice from 3 independent experiments)

(C) Confocal imaging of testis sections from 6-week-old *Cdh5*^{creER}; *R26*^{tdTomato} mice that were stained with anti-CD31 antibody. Mice were treated with tamoxifen at 4 weeks of age. (n=3 mice from 3 independent experiments)

(D) Relative expression of *Scf* transcripts in sorted Tomato^{+} cells from *Cdh5*^{creER}; *Scf*$^{fl/fl}$; *R26*^{tdTomato} (*cKO*) and *Cdh5*^{creER}; *R26*^{tdTomato} mice (CT) treated with tamoxifen at 4-weeks of age. ***p < 0.001 (n=3 mice from 3 independent experiments)

(E) Confocal imaging of testis sections from 6-week-old *Sma-creER; R26*^{tdTomato} mice that were stained with anti-SMA antibody. Mice were treated with tamoxifen at 4 weeks of age. (n=3 mice from 3 independent experiments)

(F) Relative expression of *Scf* transcripts in sorted Tomato^{+} cells from *Sma-creER; Scf*$^{fl/fl}$; *R26*^{tdTomato} (*cKO*) and *Sma-creER; R26*^{tdTomato} mice (CT) treated with tamoxifen at 4-weeks of age. **p < 0.01 (n=3 mice from 3 independent experiments)

(G) Relative expression of *Scf* transcripts in sorted Tomato^{+} cells from *Tcf21*^{creER}; *Scf*$^{fl/fl}$; *R26*^{tdTomato} (*cKO*) and *Tcf21*^{creER}; *R26*^{tdTomato} mice (CT) treated with tamoxifen at 4-weeks of age. ***p < 0.001 (n=3 mice from 3 independent experiments)

Development • Supplementary information

(H) Quantification results of (A-E) showing the labelling efficiencies of Tomato in Leydig cells, endothelial cells and vascular smooth muscle cells in testes from *Kit*^{MerCreMer}*; R26*^{tdTomato}, *Cdh5*^{creER}*; R26*^{tdTomato} and *Sma-creER; R26*^{tdTomato} mice, respectively.

(I) Western-blot analysis revealed efficient depletion of SCF protein from *Amh-cre; Cdh5*^{creER}*; Scf*$^{fl/fl}$, *Kit*^{creER}*; Scf*$^{fl/fl}$, *Sma-creER; Scf*$^{fl/fl}$*; R26*^{tdTomato} and *Tcf21*^{creER} *; Scf*$^{fl/fl}$*; R26*^{tdTomato} mice. Procedure of cell preparation was described in the method section. (n=3 mice per genotype from 3 independent experiments)

(J) Quantification of the western blots in (I). Expression levels of SCF protein were normalized to Vinculin. $^{**}p < 0.01$, $^{***}p < 0.001$.

Development • Supplementary information

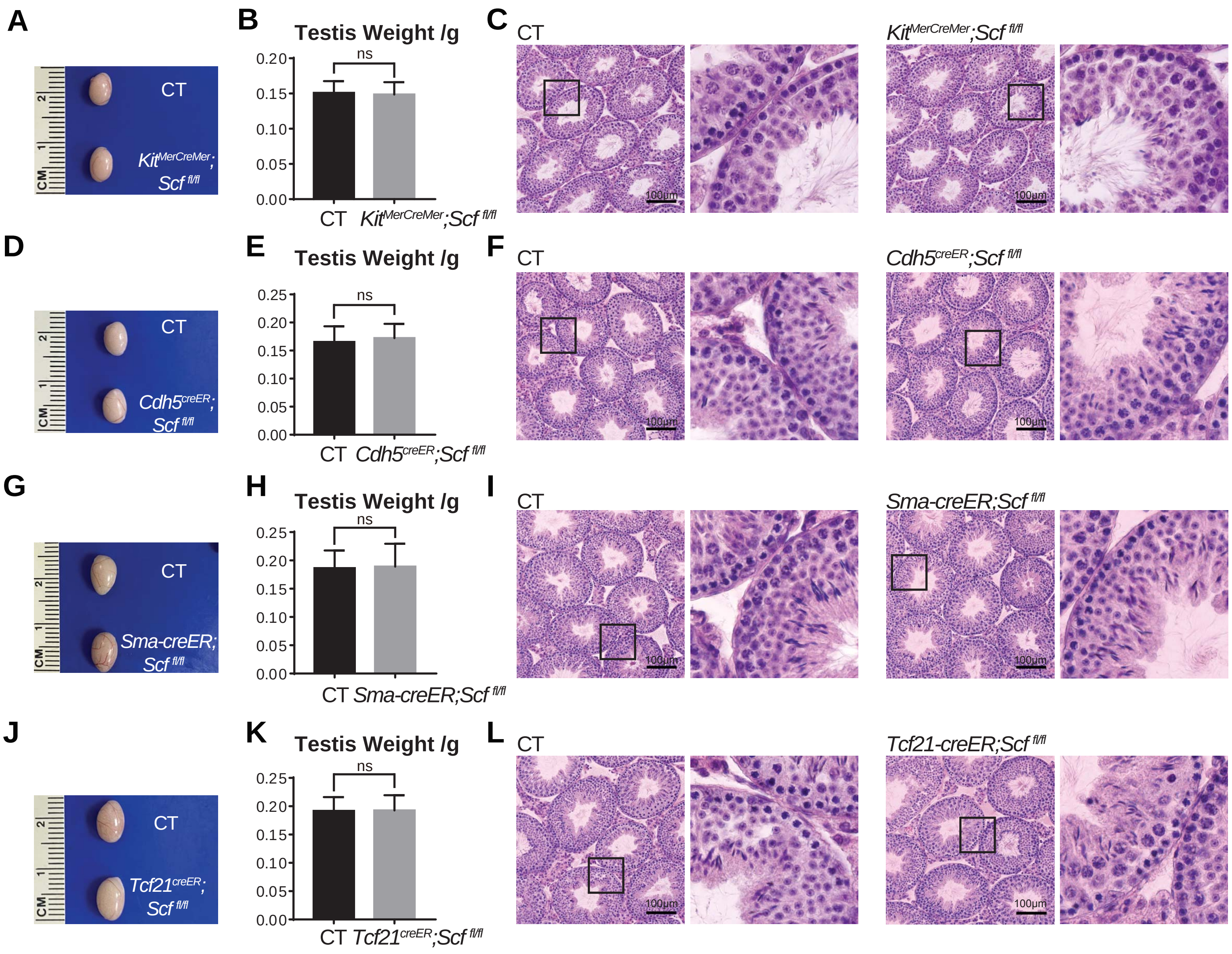

Fig.S3. Deletion of *Scf* from Sertoli cells, but not from Leydig, endothelial, smooth muscle cells or *Tcf21*$^{+}$ peritubular mesenchymal cells, blocks spermatogenesis.

(A-C) The size (A), weight (B) and H&E-stained sections (C) of testes from 12-week-old *Kit*^{MerCreMer}; *Scf*$^{fl/fl}$ and control mice treated with tamoxifen once every two days from 4 to 12 weeks of age. Two-tailed Student's t test was used to assess statistical significance. (n=5 mice from 3 independent experiments)

(D-F) The size (D), weight (E) and H&E-stained sections (F) of testes from 12-week-old *Cdh5-creER; Scf*$^{fl/fl}$ and control mice treated with tamoxifen once every two days from 4 to 12 weeks of age. Two-tailed Student's t test was used to assess statistical significance. (n=5 mice from 3 independent experiments)

Development • Supplementary information

(G-I) The size (G), weight (H) and H&E-stained sections (I) of testes from 12-week-old *Sma-creER; Scf*$^{fl/fl}$ and control mice treated with tamoxifen once every two days from 4 to 12 weeks of age. Two-tailed Student's t test was used to assess statistical significance. (n=5 mice from 3 independent experiments)

(J-L) The size (J), weight (K) and H&E-stained sections (L) of testes from 12-week-old *Tcf21*^{creER}*; Scf*$^{fl/fl}$ and control mice treated with tamoxifen once every two days from 4 to 12 weeks of age. Two-tailed Student's t test was used to assess statistical significance. (n=5 mice from 3 independent experiments)

Development • Supplementary information

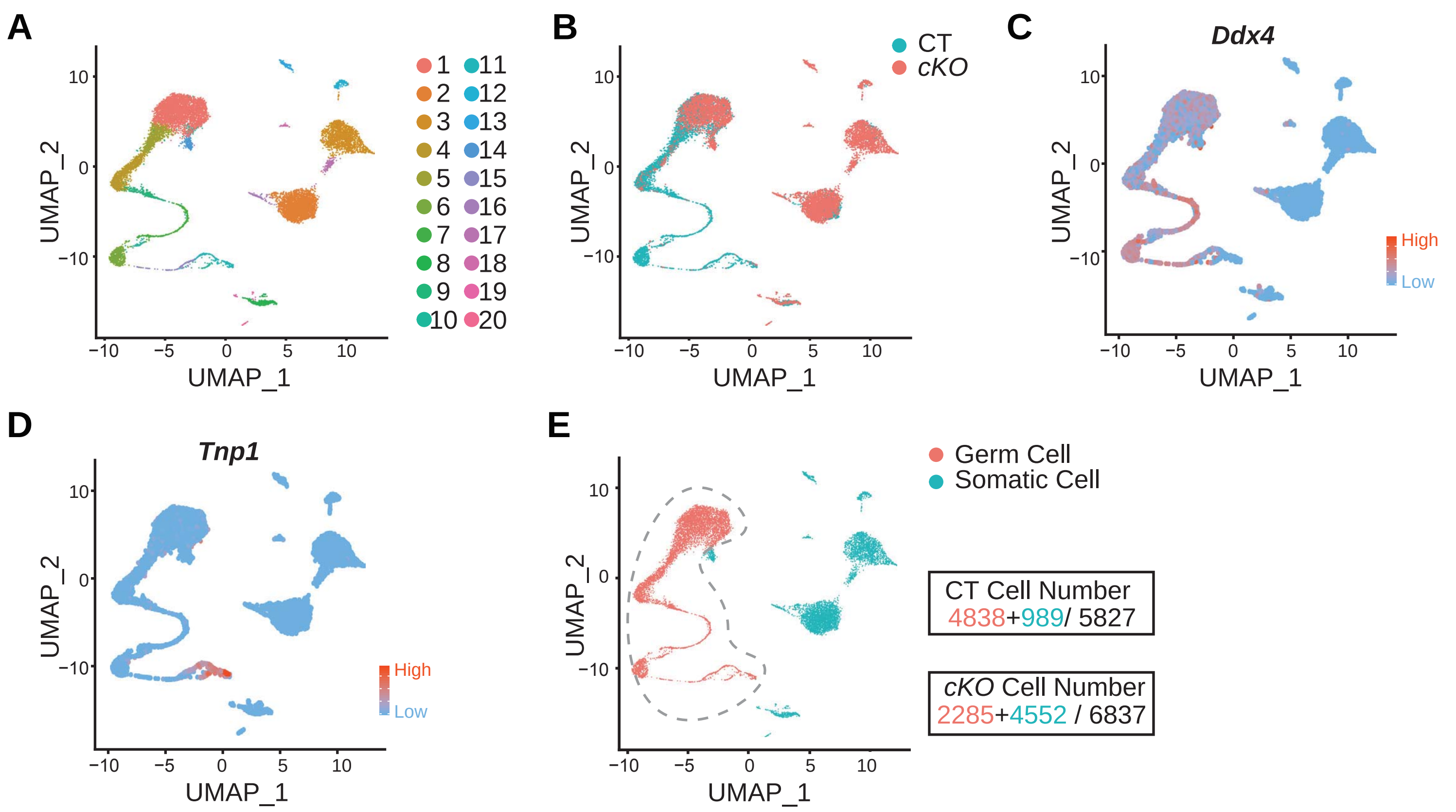

Fig. S4. Clustering analysis of the integrated scRNA-seq data from control and *Scf* deficient testicular cells.

(A) UMAP and clustering analysis of integrated single-cell transcriptome data from control and *Scf* conditional knockout testicular cells.

(B) Visualization of the source of each cell in the UMAP space.

(C, D) UMAP plots showing expression pattern of germ cell specific marker gene *Ddx4* (C) or elongating spermatid marker gene *Tnp1* (D).

(E) UMAP plot showing the assigned germ cell cluster and somatic cell cluster. The numbers of germ cells and somatic cells of testes from 6-week-old *Scf*$^{fl/fl}$ and *Amh-cre; Scf*$^{fl/fl}$ mice are shown. Gene expression matrix for the gated cells was extracted for further analyses.

Development • Supplementary information

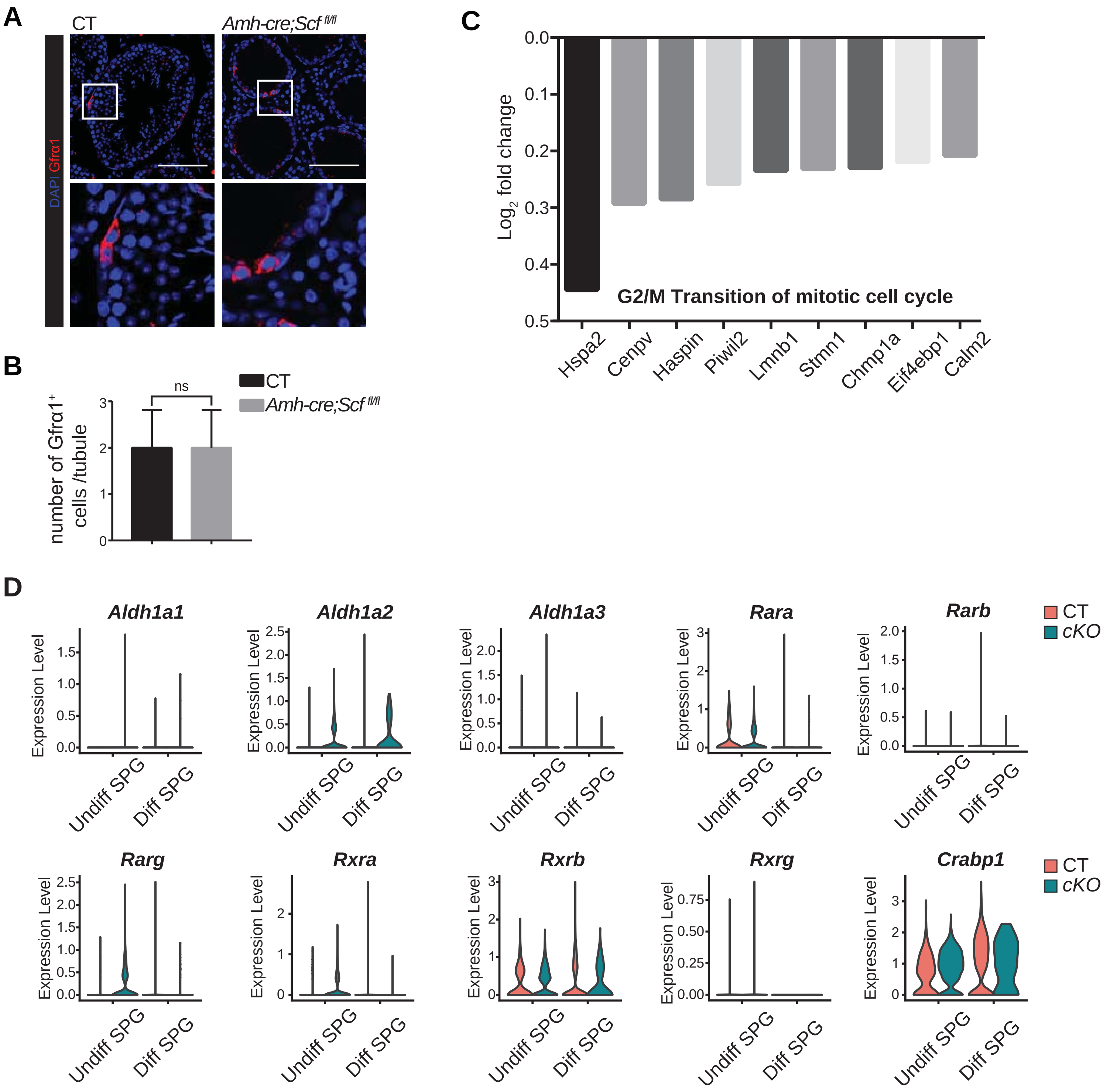

Fig. S5. Comparison analysis of scRNA-seq profiles of *Amh-cre; Scf*$^{fl/fl}$ and control spermatogonia

(A-B) Confocal imaging of testis sections from 6-week-old old *Amh-cre; Scf*$^{fl/fl}$ and control mice that were stained with anti-Gfrα1 antibody (A). The number of Gfrα1^{+} spermatogonia (B) per tubule were quantified according to the imaging results.

(C) Relative expression levels of DEGs which are enriched in G2/M transition of mitotic cell cycle.

(D) Violin plots showing the expression patterns of key regulators involved in RA signaling in undifferentiated or differentiating spermatogonia from *Amh-cre; Scf*$^{fl/fl}$ and control mice.

Development • Supplementary information

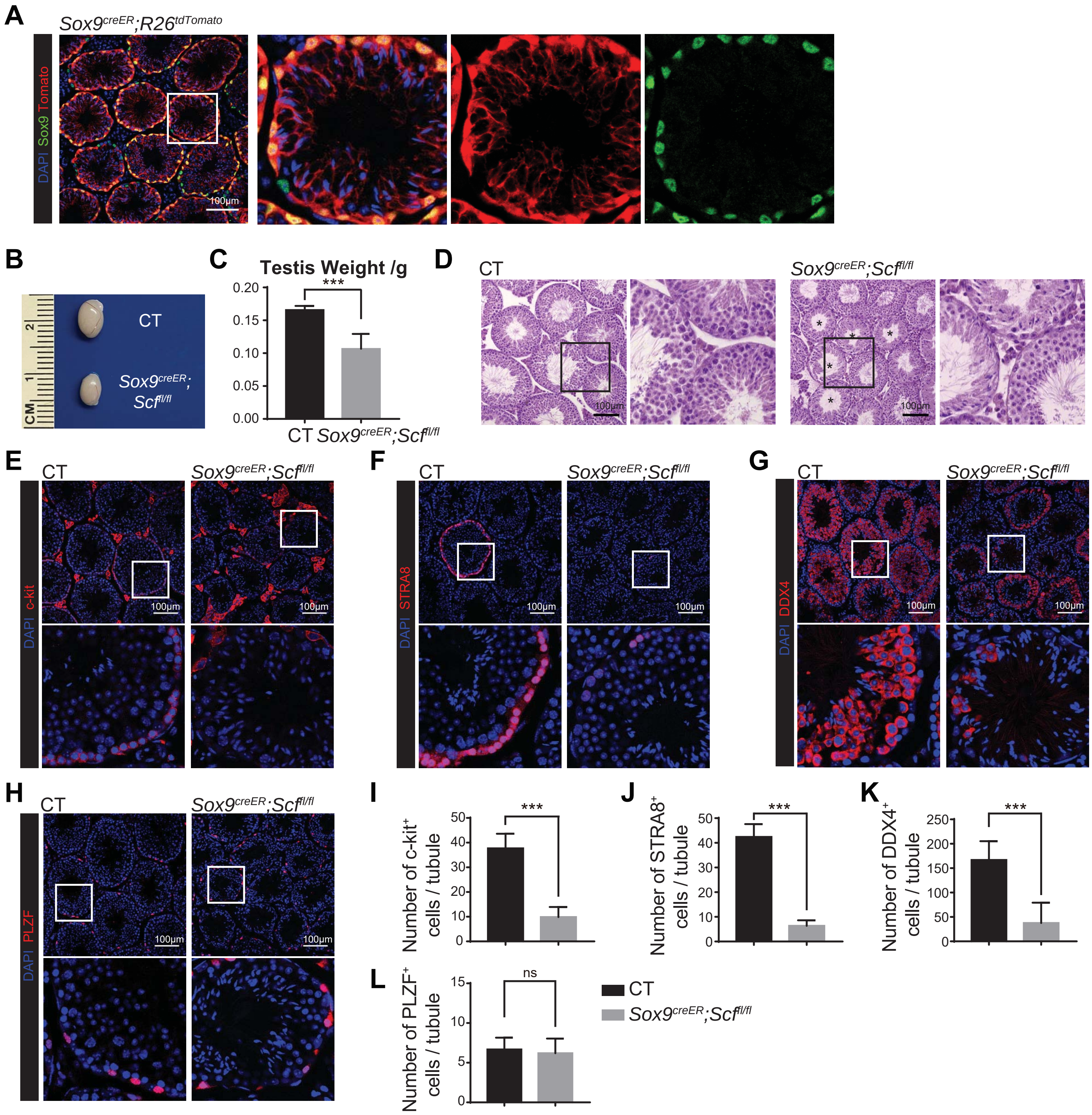

Fig. S6. Comparison analysis of testes from 6-week-old *Sox9*^{creER}*; Scf*$^{fl/fl}$ and control mice

(A) Confocal imaging of testis sections from 6-week-old *Sox9*^{creER}*; R26*^{tdTomato} mice that were stained with anti-SOX9 antibody. Mice were treated with tamoxifen at 4 weeks of age. (n=3 mice from 3 independent experiments)

(B-D) The size (B), weight (C) and H&E-stained sections (D) of testes from 6-week-old *Sox9*^{creER}*; Scf*$^{fl/fl}$ and control mice. Mice were treated with tamoxifen at 4 weeks of age. Two-tailed Student's t test was used to assess statistical significance. **p < 0.01 (n=5 mice from 3 independent experiments)

Development • Supplementary information

(E-L) Confocal imaging of testis sections from 6-week-old old *Sox9*^{creER}; *Scf*$^{fl/fl}$ and control mice that were stained with anti-c-kit (E), anti-STRA8 (F), anti-DDX4 (G) and anti-PLZF antibody (H), respectively. The numbers of c-kit^{+} (I), STRA8^{+} (J), DDX4^{+} (K), PLZF^{+} (L) germ cells per tubule were quantified according to the imaging results. Two-tailed Student's t tests were used to assess statistical significance. ns, not significant; ***$p < 0.001$ (n=5 mice from 3 independent experiments)

Development • Supplementary information

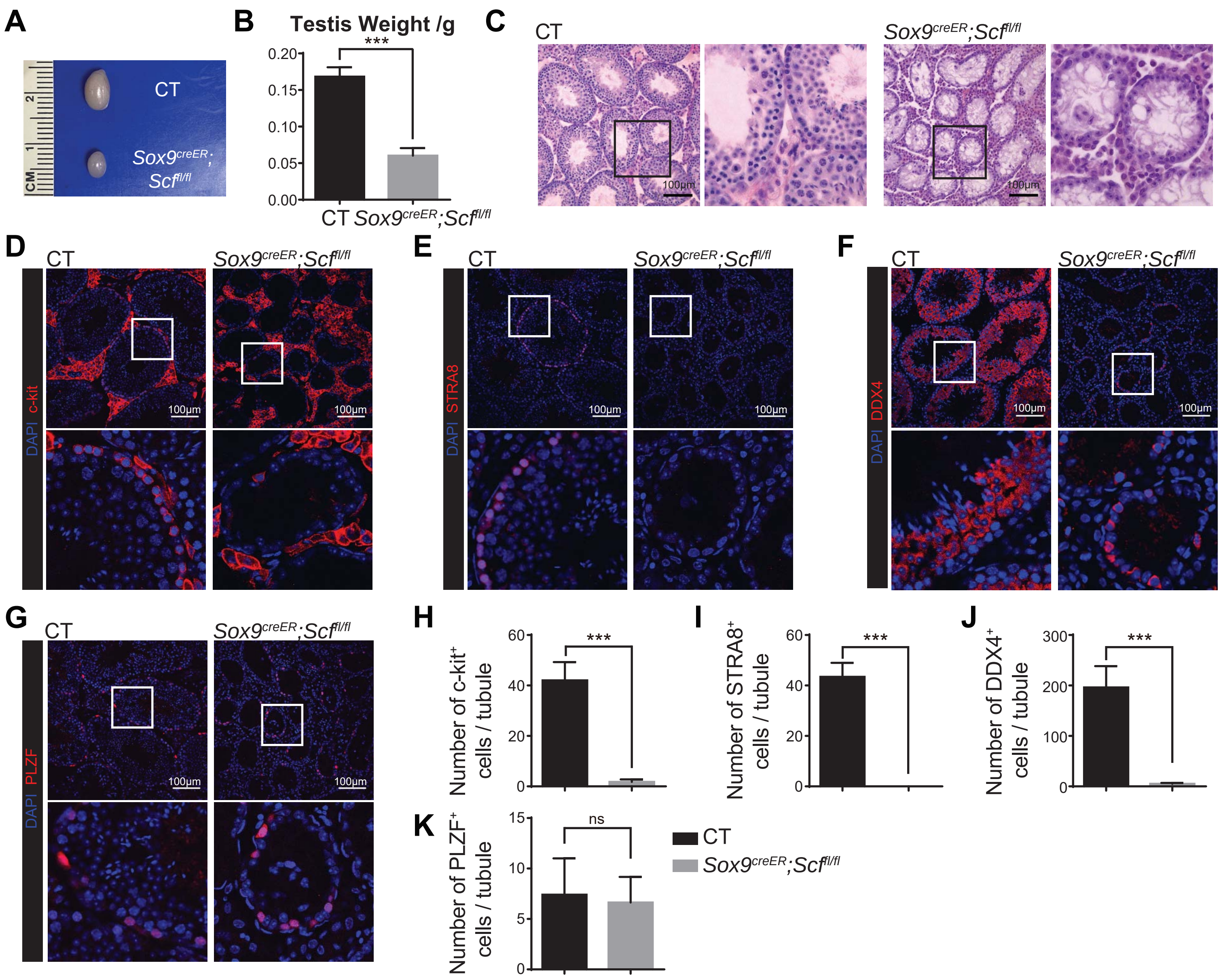

Fig. S7. Comparison analysis of testes from 12-week-old *Sox9^creER^; Scf^fl/fl^* and control mice

(A-C) The size (A), weight (B) and H&E-stained sections (C) of testes from 12-week-old *Sox9^creER^; Scf^fl/fl^* and control mice. Mice were treated with tamoxifen once every two days from 4 to 12 weeks of age. Two-tailed Student's t test was used to assess statistical significance. ***p < 0.001 (n=5 mice from 3 independent experiments)

(D-G) Confocal imaging of testis sections from 12-week-old old *Sox9^creER^; Scf^fl/fl^* and control mice that were stained with anti-c-kit (D), anti-STRA8 (E), anti-DDX4 (F) and anti-PLZF antibody (G), respectively. The numbers of c-kit$^+$ (H), STRA8$^+$ (I), DDX4$^+$ (J), PLZF$^+$ (K) germ cells per tubule were quantified according to the imaging results. Two-tailed Student's t tests were used to assess statistical significance. ns, not significant; ***p < 0.001 (n=5 mice from 3 independent experiments)

Development • Supplementary information

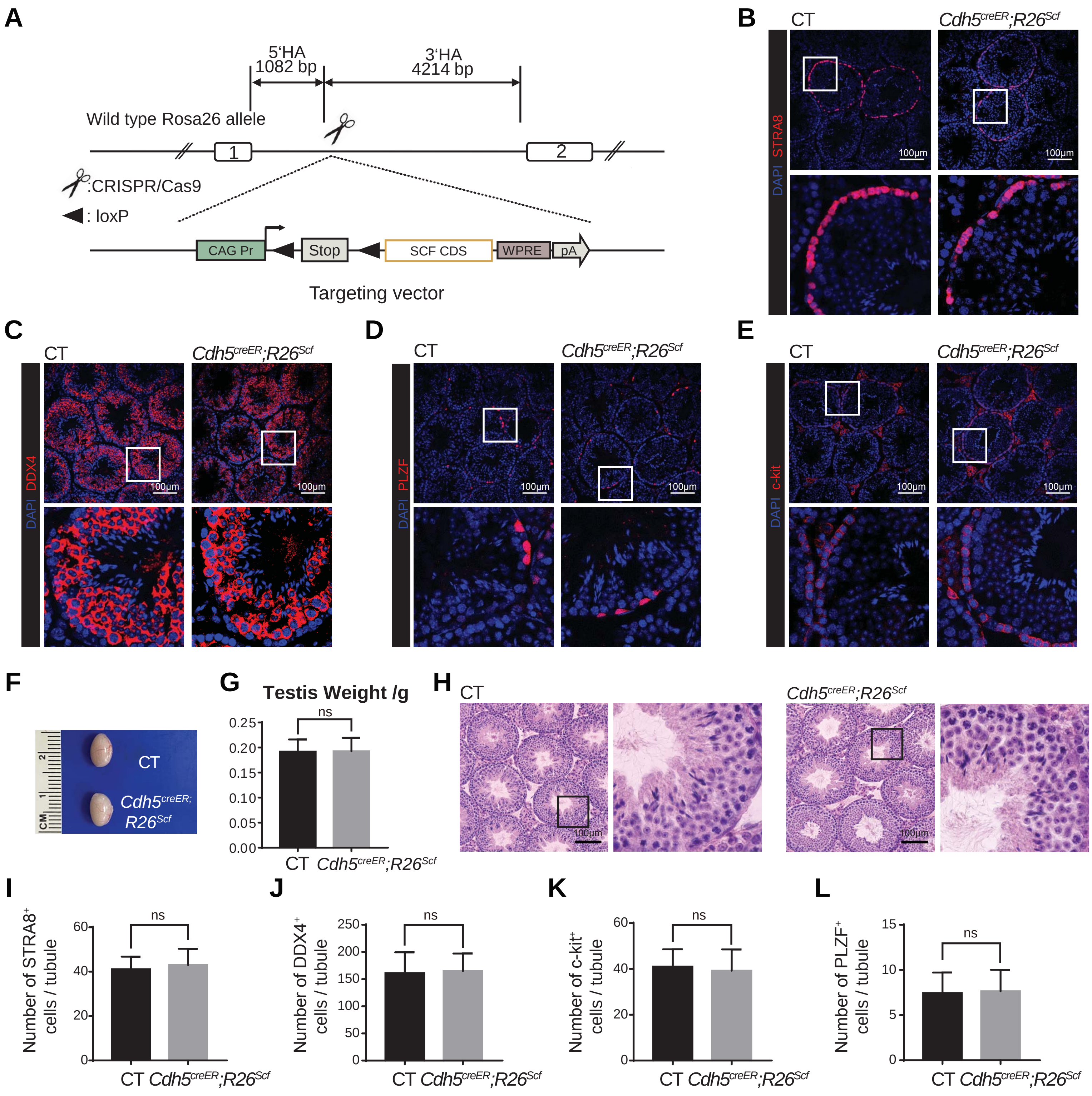

Fig. S8. Overexpression of *Scf* from endothelial cells does not affect spermatogenesis.

(A) Schematic showing the strategy for generating *R26*^{Scf} knockin mice.

(B-E) Confocal imaging of testis sections from 6-week-old *Cdh5*^{creER}*; R26*^{Scf} and control mice that were stained with anti-STRA8 (B), anti-DDX4 (C), anti-c-kit (D), and anti-PLZF (E), respectively. Mice were treated with tamoxifen at 4 weeks of age.

(F-H) The size (F), weight (G) and H&E-stained sections (H) of testes from 12-week-old *Cdh5*^{creER}*; R26*^{Scf} and control mice. Mice were treated with tamoxifen every two days at 4 weeks of age. Two-tailed Student's t test was used to assess statistical significance. ns, not significant (n=5 mice from 3 independent experiments)

Development • Supplementary information

(I-L) Quantification of the STRA8$^+$ (I), DDX4$^+$ (J), c-kit$^+$ (K), and PLZF$^+$ (L) germ cells of 12-week-old *Cdh5^{creER}; R26^{Scf}* and control mice after long-term tamoxifen treatment. 4-week-old mice were treated with tamoxifen every two days before analysis at 12 weeks of age. Two-tailed Student's t tests were used to assess statistical significance. ns, not significant (n=5 mice from 3 independent experiments)

Development • Supplementary information

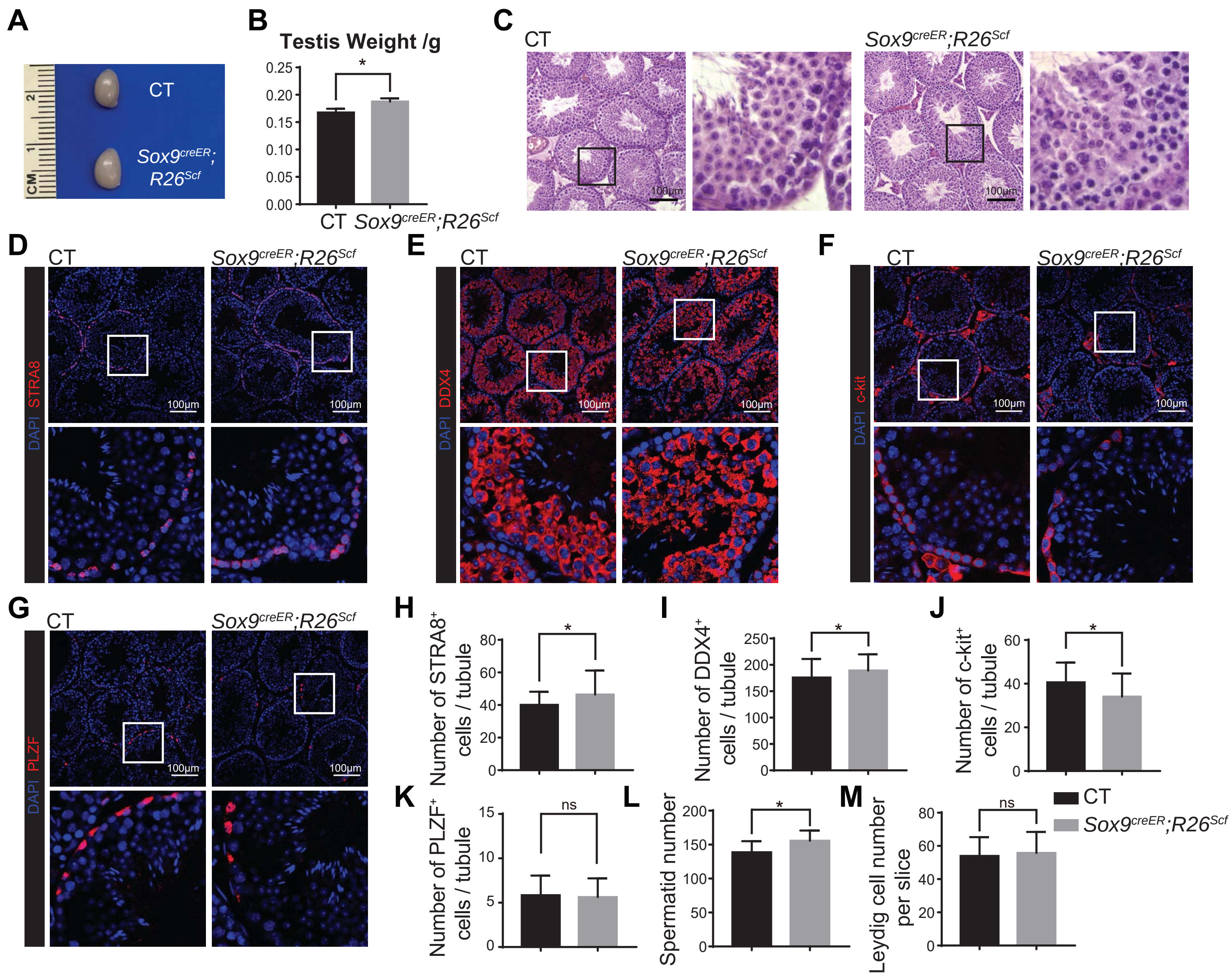

Fig. S9. Overexpression of *Scf* from Sertoli cells by *Sox9*-creER increased spermatogenesis. (A-C) The size (A), weight (B) and H&E-stained sections (C) of testes from 6-week-old *Sox9^creER^; R26^Scf^* and control mice. Mice were treated with tamoxifen at 4 weeks of age. Two-tailed Student's t test was used to assess statistical significance. *p < 0.05 (n=5 mice from 3 independent experiments)

(D-M) Confocal imaging of testis sections from 6-week-old *Sox9^creER^; R26^Scf^* and control mice that were stained with anti-STRA8 (D), anti-DDX4 (E), anti-PLZF (F) and anti-c-kit antibody (G), respectively. The numbers of STRA8⁺ (H), DDX4⁺ (I), c-kit⁺ (J), PLZF⁺ (K) germ cells and spermatids (L) per tubule and c-kit⁺ Leydig cells (M) per slice were quantified according to the imaging results. Mice were treated with tamoxifen at 4 weeks of age. Counting of spermatids was based on morphology and DAPI staining. Two-tailed Student's t tests were used to assess statistical significance. ns, not significant; *p < 0.05 (n=5 mice from 3 independent experiments)

Development • Supplementary information

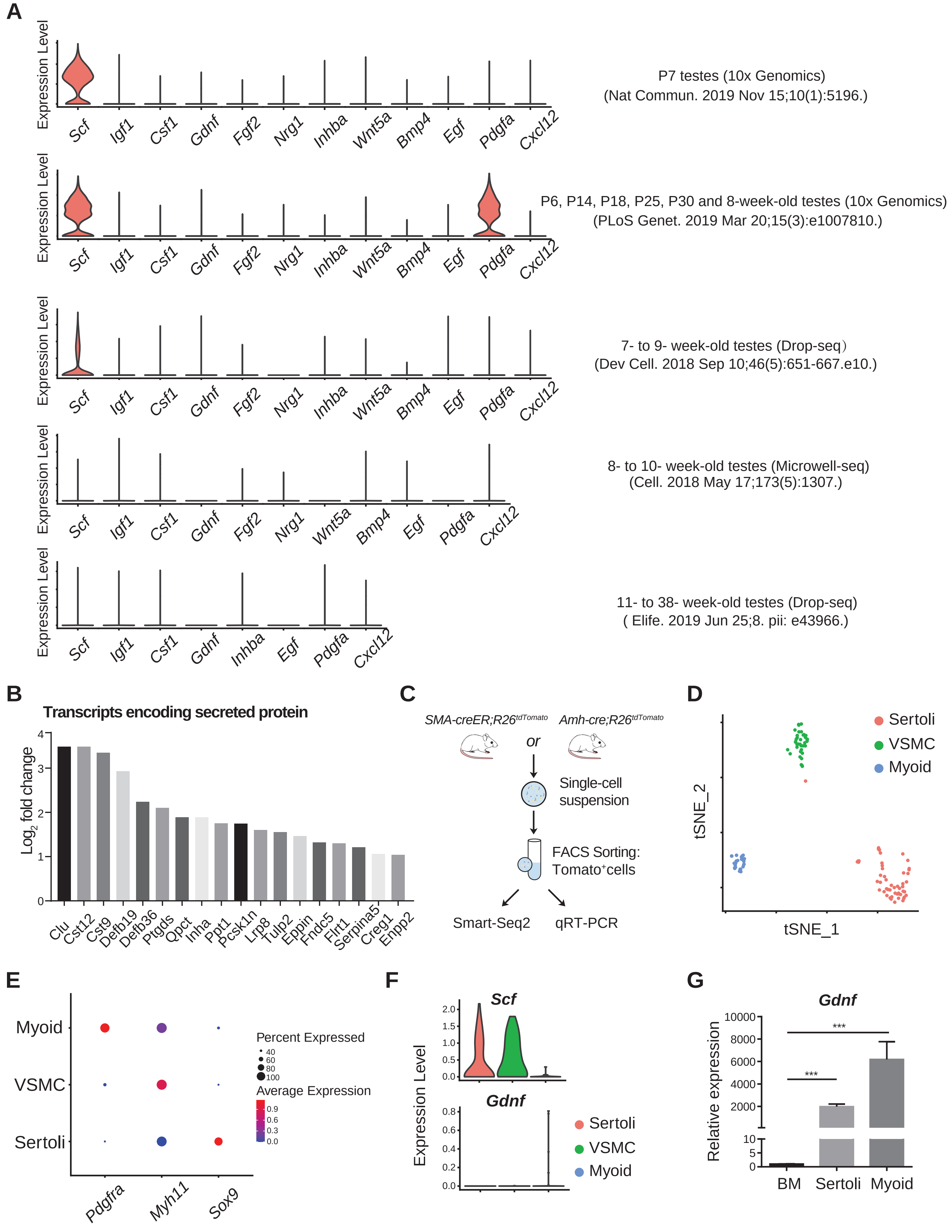

Development • Supplementary information

Fig. S10. qRT-PCR, but not scRNA-seq, detected robust expression of Gdnf in Sertoli cells

(A) ScRNA-seq in published literatures did not detect *Gdnf* in Sertoli cells. Violin plots showed the expression levels of many known growth factors in Sertoli cells.

(B) Relative expression levels of differentially expressed gene (DEGs) encoding secreted protein in Sertoli cells.

(C) Schematic overview of the workflow for sample preparation for scRNA-seq analysis by Smart-seq2 and transcriptional expression analysis by qRT-PCR.

(D) Clustering analysis of single-cell transcriptome data from combined Tomato^{+} testicular cells sorted from *Amh-cre;R26*^{tdTomato} and *Sma-creER;R26*^{tdTomato} mice.

(E) Dot-plot showing the expression patterns of distinct cell specific marker genes in three cell clusters.

(F) Violin plots showing the expression levels of *Scf* (top panel) and *Gdnf* (bottom panel) in Sertoli cells, myoid cells and vascular smooth muscle cells (VSMC).

(G) qRT-PCR detected robust Gdnf expression in Sertoli cells and myoid cells. Whole bone marrow cells (BM) were set as negative control. **$p < 0.01$, ***$p < 0.001$ (n=3 mice from 3 independent experiments)

Development • Supplementary information
