# Supplementary material for: Sertoli cells are the source of stem cell factor for spermatogenesis
Source: Development. 2023 Mar 20;150(6):dev200706. doi: 10.1242/dev.200706 (PMC10112922; doi:10.1242/dev.200706)
Supplement: Supplementary file 1 [file develop-150-200706_review_history.pdf]

Sertoli cells are the source of stem cell factor for spermatogenesis

Yi Jacky Peng, Xinyu Thomas Tang, Hui Sophie Shu, Wenjie Dong, Hongfang Shao and Bo O. Zhou
DOI: 10.1242/dev.200706

Editor: Haruhiko Koseki

Review timeline

Original Submission:	25 October 2021
Editorial Decision:	25 November 2021
Resubmission:	28 February 2022
Editorial decision:	23 March 2022
First revision received:	11 April 2022
Editorial decision:	31 May 2022
Second revision received:	16 June 2022
Editorial decision:	13 July 2022
Third revision received:	26 October 2022
Editorial decision:	25 November 2022
Fourth revision received:	21 January 2023
Editorial decision:	9 February 2023
Fifth revision received:	12 February 2023
Accepted:	17 February 2023

Original submission

First decision letter

MS ID#: DEVELOP/2021/200281

MS TITLE: Sertoli cells are the unique source of stem cell factor for spermatogenesis

AUTHORS: Yi Jacky Peng, Xinyu Thomas Tang, Hui Sophie Shu, Wenjie Dong, Hongfang Shao, and Bo O Zhou
ARTICLE TYPE: Research Article

Dear Dr. Zhou,

I have now received all the referees reports on the above manuscript, and have reached a decision. The referees' comments are appended below, or you can access them online: please go to BenchPress and click on the 'Manuscripts with Decisions' queue in the Author Area.

As you will see from their reports, the referees recognise the potential of your work, but they also raise significant concerns about it. Given the nature of these concerns, I am afraid I have little choice other than to reject the paper at this stage.

However, having evaluated the paper, I do recognise the potential importance of this work. I would therefore be prepared to consider as a new submission an extension of this study that contains new experiments, data and discussions and that address fully the major concerns of the referees. The work required goes beyond a standard revision of the paper. Please bear in mind that the referees (who may be different from the present reviewers) will assess the novelty of your work in the

© 2023. Published by The Company of Biologists under the terms of the Creative Commons Attribution License (https://creativecommons.org/licenses/by/4.0/).

context of all previous publications, including those published between now and the time of resubmission.

Reviewer 1 Advance Summary and Potential Significance to Field:

In this work, the authors assess the expression of Scf in the testis and systematically address the requirement of Scf function in different testicular cell types for spermatogenesis. The defining of the expression and function of a critical niche factor would help the field understand basic mechanisms of cellular differentiation within an anatomical niche.

Reviewer 1 Comments for the Author:

In this study, Peng and colleagues examine the expression of SCF (stem cell factor/kit ligand) in the testis and its role as a niche factor for spermatogenesis. While multiple testicular cell types express SCF, it is unclear which cell type provides the key necessary source of SCF. The authors use single-cell RNA-Seq to verify that multiple adult testicular cell types express Scf (Sertoli, endothelial, smooth muscle, Leydig, and unknown interstitial mesenchyme), and they use several mouse lineage-tracing models and conditional deletion models to examine the expression pattern of SCF and its requirement in those cell types in a systematic fashion. They found that deletion of Scf in Sertoli cells led to significant defects in spermatogenesis, while deletion in any other cell type did not have any impact. Using scRNA-Seq and tissue histology, they found that differentiating spermatogonia and more differentiated cell types were reduced in Sertoli conditional knockouts, consistent with previous findings in the field, while undifferentiated spermatogonia were unaffected. To assess which cellular source of SCF was sufficient to induce spermatogenesis, they generated a Cre-responsive Rosa-Scf model, and found that Sertoli-derived SCF, but not endothelial-derived SCF, induced increased spermatogenesis. Overall, the authors propose that Sertoli-cell-derived SCF is necessary and sufficient within the testicular niche for spermatogenesis.

Defining the anatomical source of niche factors within organs is essential to understanding stem cell niches; therefore, this work is of interest to the field and is potentially significant. The authors have attempted to systematically address different cell types of the testis, and the experimental rationale is sound. While the data are generally consistent with the author's conclusions, there are some technical issues with experimental design that lead to some concerns about how strongly the data supports the authors' claims. Furthermore, given our previous knowledge about Scf's general role in maintaining differentiating spermatogonia, more granular and mechanistic details are needed to support the authors' proposed model. In particular, the following points could be addressed:

1.One major experimental design concern deals with the fact that the Sertoli model uses Amh-Cre, which is a constitutive Cre line that is active in the fetal testis starting as early as E13.5, whereas all the other models are CreER-based, in which the analyses were performed less than 2 weeks after the end of tamoxifen induction. Therefore, this is not a direct comparison among the different models. It is possible that this difference in timing is a critical confounding factor in the data, but it is not addressed. This point is applicable both to the knockout experiments as well as the Rosa-Scf overexpression experiments.

2.In addition to the timing issue brought up in comment #1, the efficiency of constitutive Cre lines versus tamoxifen-induced CreER lines is also another potential confounding variable. The use of multiple tamoxifen injections likely addresses this issue, but it still should be addressed. The authors have done a good job in Figure S2 of showing that Scf mRNA is virtually absent in Tomato+ cKO cells, but this analysis is biased toward cells in which CreER has been activated. As currently presented, the authors have not systematically assessed the efficiency of the CreER lines (for example, flow sorting all CD31+cells versus Cdh-CreER;Rosa-Tomato+ cells and assessing Scf mRNA levels or, alternatively, rigorously quantifying Tomato expression in cell types of interest via immunofluorescence for cell-type-specific markers).

3.There is a technical issue with the smooth muscle cell analysis in Figure 3A-C. The Scf-GFP images clearly show that GFP is only expressed in perivascular smooth muscle cells and not in peritubular myoid cells. This finding somewhat confounds the flow cytometry analyses in Figure 3C, i.e., is the

© 2023. Published by The Company of Biologists under the terms of the Creative Commons Attribution License (https://creativecommons.org/licenses/by/4.0/).

low number of GFP-expressing Tomato positive cells due to heterogeneous expression within perivascular cells, or merely representative of the fact that there are far fewer perivascular smooth muscle cells versus peritubular myoid cells? Is it possible via RNA-Seq, imaging, or other methods to address this point by distinguishing between perivascular and peritubular smooth muscle in these analyses? In any case, the authors should be more specific in the text and state that "perivascular smooth muscle cells are a source of SCF in the testis" (page 8, lines 11-12).

4.It is formally difficult for the authors to claim that Sertoli-cell-derived SCF is "sufficient for spermatogenesis" (line 11 in the Abstract) in a normal context, since the authors did not ablate all other (non-Sertoli) sources of SCF at the same time. The Amh-Cre;Rosa-Scf overexpression experiments in Figure 7 potentially indicate that Sertoli-cell-derived SCF is itself potent enough to induce extra spermatogenesis (albeit this data is somewhat problematic in itself…see comment #5), but this may be due merely to the amount of SCF produced by Sertoli cells, rather than their anatomical location (also related to comment #10).

5.There are several key experiments missing from the Rosa-Scf experiments in Figure 7. One is measuring the degree to which SCF overall is increased in the testis in the Sertoli cell model versus the endothelial cell model. Another is assessing if other cell types are affected. Given the rather large increase in testis size (about 33% increase in testis weight), there are rather minor changes in STRA8+ and DDX4+ cells (Figure 7). Therefore, there should be a more rigorous analysis of this phenotype. Does this translate into a higher sperm count, thicker germinal epithelium, more spermatids, less cell death earlier in spermatogenesis, etc.?

6.There is a technical concern regarding the GFP gating in the flow cytometry experiments of Figures 2 and 3. It is understandable why the Tomato gates are different sizes and brightness in every experiment, since the Rosa locus may express at different levels in different cell types. However, it is very unclear why the GFP gates are all different sizes and brightnesses in each experiment; in theory, these plots (bottom left flow plot of each panel) should be virtually identical in every panel. It is unclear why the flow profile of "all GFP+" cells from KitCreER;Scf-GFP mice would be different from Tcf21-CreER;Scf-GFP mice. This technical issue leads to some concerns about the results and their interpretation.

7.Also, in Figure 7 it is unclear how "exhaustion of c-kit+ spermatogonia" (page 15, lines 1-2), which directly give rise to STRA8+ cells, leads to an increased number of STRA8+ cells. Does this suggest that C-KIT+ cells transit into meiosis more quickly than normal and, therefore, do not remain C-KIT+ for long? The mechanism for this phenotype is unclear and should be addressed or discussed.

8.Furthermore, the immunofluorescence image in Figure 7F seems to show a significant increase in KIT+ Leydig cells in the interstitium of Amh-Cre;Rosa-Scf testes. Could such a change cause the increase in testis size? Are there any effects on steroid production, which could also impact germ cells? Since Leydig cells also express KIT receptor, they could potentially be impacted by Amh-Cre-mediated SCF overexpression; therefore, this possibility should be examined.

9.Related to the previous comment, why did the authors only perform Scf overexpression in Sertoli cells and endothelial cells? The rationale for this choice is unclear. They did a very thorough and systematic job of addressing all the cell types in the knockout experiments, but in comparison the overexpression experiments seem incomplete.

10.Is it possible that Sertoli cells are not particularly important due to their localization, but rather due to their overwhelming dominance in terms of numbers (Sertoli cells are >80% of Scf-GFP+ cells in the testis; Figure 2) and expression levels of SCF (i.e., Scf-GFP expression seems much brighter in Sertoli cells versus interstitial cells)? Is it possible that merely injecting SCF into the mice or into the interstitium of the testis would cause a similar effect as the Amh-Cre;Rosa-Scf overexpression? This would argue against claims made by the authors that Sertoli cells are unique in the niche due to their localization and proximity to the germ cells, and that "direct interaction between the niche and its target cells is pivotal for the action of niche factors" (page 16, lines 16-17).

11.Given that the authors generated the Cdh5-CreER and Tcf21-CreER lines, more information in the Methods should be provided regarding their construction. Especially for the Tcf21-CreER line, its expression pattern should be defined more rigorously, since it is unclear exactly where the

© 2023. Published by The Company of Biologists under the terms of the Creative Commons Attribution License (https://creativecommons.org/licenses/by/4.0/).

CreER is active. In the Figure Legend for Figure 3J-K, the authors claims that the arrowheads point to Tomato expression in "Tomato+SMA- peritubular mesenchymal cells" (page 37, lines 6-7). However, these cells clearly look like endothelial or perivascular cells (for example, see the Tomato+ ring of cells around the large arteriole in the low-magnification panel of Figure 3K). So these lines should be re-visited and verified more carefully. In light of this issue, the authors should re-consider stating that "Tcf21+ peritubular mesenchymal cells are a source of SCF" (page 10, lines 1-2).

12.On page 11 (lines 3,9,15, and 21), how was a "normal number of germ cells in the seminiferous tubules" assessed?

13.The authors claim on page 13, lines 10-12, that "deletion of Scf from Sertoli cells depletes differentiating spermatogonia…without affecting undifferentiated spermatogonia." However, this analyses is simply based on cell counts for PLZF, which marks a broad population of cells. Analysis of other markers, such as GFRA1, would give more insight. Furthermore, delving into their sc-RNA-Seq data of the undifferentiated spermatogonial population, similar to what was done for the differentiating spermatogonial population in Figure 5, would also provide granular insight into that population of cells.

14.Can the authors clarify how "…deletion of Scf from Sertoli cells blocks spermatogenesis by depleting differentiating spermatogonia" (page 13, lines 3 and 11)? Given that the general role of Scf in spermatogonia is already known, more specific, mechanistic insight would help advance the field further. For example, is the Sertoli cKO "depletion" phenotype due to increased cell death of differentiating spermatogonia, changes in spermatogonial cell cycle, etc.?

15.The data in Figure 5 are consistent with a "defect in meiosis entry" (page 14, line 2); however, due to a near-complete lack of C-KIT+ cells (Figure 6D), it is likely that the main defects in spermatogonia are in processes of differentiation prior to meiotic entry.

Minor points:

1.It would help to define (in the Figure Legends) the gray population in the histograms for the flow cytometry experiments in Figures 2 and 3 (i.e., what type of negative control population it is).

2.It looks like the y-axis scale in Figure 6I may be incorrect. It is an order of magnitude smaller than the y-axis labels of the corresponding similar graphs in Figures 6L, 6O, and 6R.

3.Some type of unit label is needed for the axes in flow cytometry plots.

Reviewer 2 Advance Summary and Potential Significance to Field:

The current study took an approach to study the expression of niche factors in the testes and eventually focused on the expression pattern and functions of the Stem cell factor.
A series of experiments using a variety of transgenic mice to follow the pattern of SCF expression as well as to analyze the results of its deletion or overexpression in different types of testicular somatic cells have been undertaken. Although the mouse models provided elegant new ways to analyze the results and to add some additional data (for example, that overexpression of SCF increases spermatogonia differentiation), the major findings have just confirmed what was well-known about the C-kit/ SCF system during spermatogenesis: SCF secretion by Sertoli cell is required for spermatogenesis, and C-kit/ SCF is required for spermatogonia differentiation. Based on these and previous findings, it is also unclear why the authors consider SCF to be a niche factor.

Reviewer 2 Comments for the Author:

SCF is not considered to be a niche factor. Niche and niche factors provide microenvironment that specifically supports spermatogonia stem cells while Stem cell factor regulates differentiated spermatogonia in postnatal testes. It is not required for the maintenance of the spermatogonia stem cells. Only differentiated spermatogonia express c-kit receptor and being depleted upon inactivation of the SCF/c-kit system.

© 2023. Published by The Company of Biologists under the terms of the Creative Commons Attribution License (https://creativecommons.org/licenses/by/4.0/).

The data in the manuscript provide evidence that other somatic cells secrete SCF beyond Sertoli cells, but also show that this secretion is not required for spermatogenesis. Therefore, the Sertoli cells are the major site of the SCF synthesis, and this fact has been shown before by other means.

It was well-expected that the authors will detect defects in spermatogonia differentiation and depletion of the meiotic and post-meiotic cells since it was demonstrated that various mutations leading to a general inactivation of SCF or c-kit, as well as an injection of anti-c-kit antibodies, produce the same phenotype. This study looked at Sertoli- (and other somatic cell-) inactivation which confirms the importance of Sertoli cells in SCF secretion and the phenotype.

The finding that Sertoli-specific overexpression of SCF increases the rate of spermatogonia differentiation is important since it may explain some of medical conditions or be potentially used in the future as a therapeutic approach.

Reviewer 3 Advance Summary and Potential Significance to Field:

In the present paper Peng-YJ and co-authors analyzed the SCF expression profile in the adult murine testis by scRNAseq and by using ScfGFP knock-in mice showing that SCF is widely expressed in different somatic cell types in the testis. Among the several factors produced by Sertoli cells that influence the activity of spermatogonia, SCF is the best studied growth factor and the signaling cascade involving its receptor c-kit has been shown to be indispensable for spermatogenesis and spermatogonial proliferation, differentiation, and survival (PMID: 11061423, PMID: 10655061). The authors next addressed the impact of cell type-specific ablation of SCF in Sertoli cell, Leydig cells and peritubular mesenchymal cells. They conclude that the conditional deletion of Scf in Sertoli cells but not in other somatic cells blocked the differentiation of spermatogonia leading to male infertility. Until recently, it was a generally assumed that Sertoli cells are the major cell type involved in the control of SSCs. The observation that, different somatic cell types regulate SSC self-renewal and differentiation, shift this paradigm (PMID: 28115580). Therefore, the objective of the present study, deals with a better understanding of the physiological source of the niche factors for SSC. The objective of the manuscript is clear and of great interest for the field.

Reviewer 3 Comments for the Author:

Major comments.
The manuscript has two major problems/limitations:
1) Definition of a niche-derived factor. According to the prevalent view in the field, SSCs are maintained within a specialized microenvironment, called a niche, that supports their self-renewal. However, a large body of evidence shows that SCF is not a niche-factor but rather promotes spermatogonial differentiation (PMID: 12890724). Therefore, the paper doesn't add any significant and novel contribution to the understanding of the anatomical or molecular features of the SSCs niche.
2) Novelty. The observation that Scf deletion blocks spermatogonial differentiation, downregulates spermatogonial mitotic entry and induce a depletion of germ cells downstream of c-kit+ spermatogonia is not novel and has been already described in the last two decades (PMID: 11061423, PMID: 10655061, PMID: 12890724).

© 2023. Published by The Company of Biologists under the terms of the Creative Commons Attribution License (https://creativecommons.org/licenses/by/4.0/).

Resubmission

First decision letter

MS ID#: DEVELOP/2022/200706

MS TITLE: Sertoli cells are the unique source of stem cell factor for spermatogenesis

AUTHORS: Yi Jacky Peng, Xinyu Thomas Tang, Hui Sophie Shu, Wenjie Dong, Hongfang Shao, and Bo O Zhou

I have now received all the referees' reports on the above manuscript, and have reached a decision. The referees' comments are appended below, or you can access them online: please go to BenchPress and click on the 'Manuscripts with Decisions' queue in the Author Area.

As you will see, both referees are happy for your experimental efforts. However, both seem to be unhappy for your paper editing. Please address all suggestion/comments raised by both reviewers appropriately. If you are able to revise the manuscript along the lines suggested, I will be happy receive a revised version of the manuscript. Your revised paper will be re-reviewed by one or more of the original referees, and acceptance of your manuscript will depend on your addressing satisfactorily the reviewers' major concerns. Please also note that Development will normally permit only one round of major revision. If it would be helpful, you are welcome to contact us to discuss your revision in greater detail. Please send us a point-by-point response indicating your plans for addressing the referee's comments, and we will look over this and provide further guidance.

Please attend to all of the reviewers' comments and ensure that you clearly highlight all changes made in the revised manuscript. Please avoid using 'Tracked changes' in Word files as these are lost in PDF conversion. I should be grateful if you would also provide a point-by-point response detailing how you have dealt with the points raised by the reviewers in the 'Response to Reviewers' box. If you do not agree with any of their criticisms or suggestions please explain clearly why this is so.

Reviewer 1

Advance summary and potential significance to field

This part remains as in my previous review:
In the present paper Peng-YJ and co-authors analyzed the SCF expression profile in the adult murine testis by scRNAseq and by using ScfGFP knock-in mice showing that SCF is widely expressed in different somatic cell types in the testis.
Among the several factors produced by Sertoli cells that influence the activity of spermatogonia, SCF is the best studied growth factor and the signaling cascade involving its receptor c-kit has been shown to be indispensable for spermatogenesis and spermatogonial proliferation, differentiation, and survival (PMID: 11061423, PMID: 10655061). The authors next addressed the impact of cell type-specific ablation of SCF in Sertoli cell, Leydig cells and peritubular mesenchymal cells. They conclude that the conditional deletion of Scf in Sertoli cells but not in other somatic cells blocked the differentiation of spermatogonia leading to male infertility. Until recently, it was a generally assumed that Sertoli cells are the major cell type involved in the control of SSCs. The observation that, different somatic cell types regulate SSC self-renewal and differentiation, shift this paradigm (PMID: 28115580). Therefore, the objective of the present study, deals with a better understanding of the physiological source of the niche factors for SSC. The objective of the manuscript is clear and of great interest for the field.

Comments for the author

The manuscript has been minimally revised in response to the three reviewers. Some of the issues raised by review #1 have been addressed; however, authors included the new data in supplementary figures without mentioning them in the revised manuscript. The major issues raised

© 2023. Published by The Company of Biologists under the terms of the Creative Commons Attribution License (https://creativecommons.org/licenses/by/4.0/).

by reviewers #2 and #3 were addressed in the response letter but the authors did not make any attempt to reshape the manuscript accordingly.

Reviewer 2

Advance summary and potential significance to field

The paper advances our understanding of regulation of spermatogonia differentiation. Using elegant genetic tools, the authors have shown that expression of SCF by Sertoli cells (but not other somatic cells) is required for spermatogonia differentiation. Using RNA seq analysis, the authors provide a possible mechanism of action of the SCF on differentiated spermatogonia. Also, the novel finding is that over-expresion of SCF production by Sertoli cells can stimulate spermatogenesis.

Comments for the author

The authors have done a good job addressing all the technical concerns and added some data regarding the possible mechanism of spermatogenic block in the mutant mice.

However, I still don't agree with the concept of the niche presented by the authors (which is different from the one accepted in the field of spermatogenesis). The author provided couple of reference for other organs where stem-like and very restricted progenitors were associated with the niche. Undifferentiated spermatogonia, however, go through several rounds of proliferation and differentiation of stem-like progenitors (A single, A paired, A aligned) before they become differentiated SCF-dependent spermatogonia. Authors states that SCF is a niche factor as a given, however, it was not defined as a niche factor in testes. The niche has a special micro-environment which is characterized by the anatomical location of being close to the basal membrane. As cells differentiate, they move away from the basement membrane. For some reasons, the authors think that Sertoli cells are anatomically located at the base of the tubule close to spermatogonia only. "This means that a spermatogonial niche is defined not only by their expression of niche factors but also by their anatomical localization. Among all the SCF-expressing cell types in the testis, Sertoli cells are the only cell type that directly interacts with undifferentiated and differentiating spermatogonia", but in fact, Sertoli cell cytoplasm extend all way to the lumen, and all types of germ cells are embedded in their cytoplasm. Depends on their confocal plane, the authors could also see the SCF-GFP signal all way to the lumen (Figure 3H, 3K etc). Furthermore, at those figures, spermatocytes and spermatids are also surrounded by the SCF-GFP signal. The signal is a bit diminishing as one goes deeper in the tubule, but again, it depends on the laser ability to reach the deeper layers and the choice of the focal plane. Sertoli cells also produce factors required for meiosis and spermiation; does that mean spermatocytes and spermatids also localize to the niche? Can authors address these concerns?

First revision

Author response to reviewers' comments

Reviewer 1:
The manuscript has been minimally revised in response to the three reviewers. Some of the issues raised by review #1 have been addressed; however, authors included the new data in supplementary figures without mentioning them in the revised manuscript. The major issues raised by reviewers #2 and #3 were addressed in the response letter but the authors did not make any attempt to reshape the manuscript accordingly.

Response: Thank the reviewer for pointing out our mistakes. We double-checked all the new data generated in the last round of revision and found that figure callouts for Fig. S2H, S4D and S6F-R were not included in the manuscript, although we did describe the results. We have included them in the revised manuscript. We also double-checked the last round response to reviewer #1 and

© 2023. Published by The Company of Biologists under the terms of the Creative Commons Attribution License (https://creativecommons.org/licenses/by/4.0/).

added more information into the revised manuscript accordingly. The changes are highlighted in red.

As suggested by the reviewer, we have also expanded our discussion section to address the concerns raised by reviewer #2 and #3.

#Reviewer:

The authors have done a good job addressing all the technical concerns and added some data regarding the possible mechanism of spermatogenic block in the mutant mice.

However, I still don't agree with the concept of the niche presented by the authors (which is different from the one accepted in the field of spermatogenesis). The author provided couple of reference for other organs where stem-like and very restricted progenitors were associated with the niche. Undifferentiated spermatogonia, however, go through several rounds of proliferation and differentiation of stem-like progenitors (A single, A paired, A aligned) before they become differentiated SCF-dependent spermatogonia. Authors states that SCF is a niche factor as a given, however, it was not defined as a niche factor in testes. The niche has a special micro-environment which is characterized by the anatomical location of being close to the basal membrane. As cells differentiate, they move away from the basement membrane. For some reasons, the authors think that Sertoli cells are anatomically located at the base of the tubule close to spermatogonia only."This means that a spermatogonial niche is defined not only by their expression of niche factors but also by their anatomical localization. Among all the SCF-expressing cell types in the testis, Sertoli cells are the only cell type that directly interacts with undifferentiated and differentiating spermatogonia", but in fact, Sertoli cell cytoplasm extend all way to the lumen, and all types of germ cells are embedded in their cytoplasm. Depends on their confocal plane, the authors could also see the SCF-GFP signal all way to the lumen (Figure 3H, 3K etc). Furthermore, at those figures, spermatocytes and spermatids are also surrounded by the SCF-GFP signal. The signal is a bit diminishing as one goes deeper in the tubule, but again, it depends on the laser ability to reach the deeper layers and the choice of the focal plane. Sertoli cells also produce factors required for meiosis and spermiation; does that mean spermatocytes and spermatids also localize to the niche?

Can authors address these concerns?

Response: We thank the reviewer for the positive feedback of our revision. Both we and the reviewer agree that Sertoli cells are the key source of niche factors and that SCF are "NOT" a niche factor for spermatogonial stem cells (SSCs). The major discrepancy is whether SCF is a niche factor for spermatogonia, in other word, whether the niche concept still stands if there is no stem cell. Our opinion is "yes". Here are our reasons:

The niche model in adult tissues is best exampled by the hematopoietic system. It is now widely accepted that, besides hematopoietic stem cells, restricted hematopoietic progenitors also reside in a specific niche and are fueled by factors from the niche (e.g., Nature, PMID: 23434755; Nature, PMID: 33627868; Cell Stem Cell, PMID: 30661958; Stem Cell Rev Rep, PMID: 28669077). More mature cells, such as erythroblasts, have also been found to reside in a specific niche, called erythroblastic island (see reviews, Blood, PMID: 18650462; Exp Hematol, PMID: 32910996). Thus, although the niche concept was initially proposed to describe the microenvironment for stem cells (Blood Cells, PMID: 747780), it is now applied to broader cell types, including progenitor cells and more mature cells.

While "testis niche" usually refers to the niche for SSCs, it may refer to the male germ cell niche in a generalized way, in which GDNF and SCF (KITL) balance the self-renewal and differentiation of spermatogonia (see review, Nat Rev Cancer, PMID:21412254). Following this logic, spermatocytes may also localize to a Sertoli cell niche if secreted factors from Sertoli cells do contribute to their maintenance and maturation. Of course, this conclusion must base on stringent in vivo evidence, as did in our study, not just on gene expression profiles.

© 2023. Published by The Company of Biologists under the terms of the Creative Commons Attribution License (https://creativecommons.org/licenses/by/4.0/).

We believe that a generalized niche concept will contribute to a better understanding of the extrinsic regulation of different cell types in vivo. We have included the description above in the discussion section of our revised manuscript. Of note, whether SCF is qualified for the term “niche factor” does not change our major conclusion that Sertoli cells are the only physiologically important source of SCF that contributes to spermatogenesis.

Second decision letter

MS ID#: DEVELOP/2022/200706

MS TITLE: Sertoli cells are the unique source of stem cell factor for spermatogenesis

AUTHORS: Yi Jacky Peng, Xinyu Thomas Tang, Hui Sophie Shu, Wenjie Dong, Hongfang Shao, and Bo O Zhou

I have now received all the referees' reports on the above manuscript, and have reached a decision. The referees' comments are appended below, or you can access them online: please go to BenchPress and click on the 'Manuscripts with Decisions' queue in the Author Area.

As you will see, all referees expressed concerns about the novelty of the study. Nonetheless, reviewers 2 and 3 also express considerable interest in your work and recommend a substantial revision of your manuscript before we can consider publication. If you are able to revise the manuscript along the lines suggested by reviewers 2 and 3, which obviously involve further experiments, I will be happy receive a revised version of the manuscript. Your revised paper will be re-reviewed by one or more of the original referees, and acceptance of your manuscript will depend on your addressing satisfactorily the reviewers' major concerns. Please also note that Development will normally permit only one round of major revision. If it would be helpful, you are welcome to contact us to discuss your revision in greater detail. Please send us a point-by-point response indicating your plans for addressing the referee’s comments, and we will look over this and provide further guidance.

Please attend to all of the reviewers' comments and ensure that you clearly highlight all changes made in the revised manuscript. Please avoid using 'Tracked changes' in Word files as these are lost in PDF conversion. I should be grateful if you would also provide a point-by-point response detailing how you have dealt with the points raised by the reviewers in the 'Response to Reviewers' box. If you do not agree with any of their criticisms or suggestions please explain clearly why this is so.

Reviewer 1

Advance summary and potential significance to field

This is technically sound paper that could be of interest for reproductive biologists working on the field of spermatogonial compartment regulation. Data described further highlights the involvement of SCF/Kit system in the paracrine control of spermatogenesis.

Comments for the author

The authors show that lack of SCF production specifically in Sertoli cells blocks differentiation of spermatogonia. Overall data support the conclusions and the role of SCF/Kit in the paracrine control of spermatogenesis. Nevertheless, the described phenotypes were expected based on previous work and they don't add significantly to our understanding of the spermatogonial stem cell niche.

© 2023. Published by The Company of Biologists under the terms of the Creative Commons Attribution License (https://creativecommons.org/licenses/by/4.0/).

Reviewer 2

Advance summary and potential significance to field

The data presented support at several levels the important conclusion of the study that Sertoli cells are the source of SCF. This factor is essential for spemratogenesis and fertility, and the source of the factor is important to identify.

Comments for the author

I am sorry that I go back and forth with the questions, but the authors want to shift the paradigm in the field changing the niche definition, so I cannot approve the current version until further details are clarified.

1) What is the new definition of the spermatogenic niche according to the authors? In all the references provided (and in general definition of the niche in all tissues), the closure to the vasculature is one of the conditions. Indeed, the current definition of the spermatogenic niche also includes the specific localization at the basement membrane and closure to the vasculature. If differentiated spermatogonia and spermatocytes can also be found at the niche according to the authors (and those cells move away from the basement membrane and blood vessels), what is the new definition of the testicular niche? It seems to me that the authors want to rename Sertoli cells that are known to be the nursing units, where all developing cells are embedded, and which produce important growth factors for the developing germ cells, as the niche. What is the importance of that and why to do so?

2) Please remove from abstract and the introduction the following statement:
"Stem cell factor (Scf), one of the essential niche factors". This information is introduced as a given one, and for the testes, SCF has not been considered as a niche factor until this study where authors aim to change this definition.

Reviewer 3

Advance summary and potential significance to field

Peng et al. systematically studied the primary sources of cell surface molecules and secretory factors from possible niche cells by scRNA-seq experiments. They continuously analyzed possible niche factors with the studies of lineage-specific conditional knockout mice and decided to focus on Stem cell factor (SCF). They identified multiple cell lineages that express Scf mRNA and went on to evaluate SCF functions as a niche factor by conditional knockout approaches. In the end, they concluded only the SCF from Sertoli cells plays a critical role in spermatogenesis.

Comments for the author

Comments

1. Quality of the data

The quality of the histological analyses is very good. The validation experiment of the lineage-specific knockout strategy was carefully done by using the reporter mice. The analyses for the next-generation sequencing, such as single-cell analyses, were performed intensively.

2. Completeness

Firstly, it is essential to confirm that the target protein was lost in the case of a conditional knockout study. However, this work lacks the evaluation of SCF protein reduction in the tissues analyzed. Furthermore, this work notes that the authors analyzed the phenotype of the conditional knockout mice after only two weeks of tamoxifen administration. Given that Sertoli cells terminally differentiate by around 17 days after birth and then stop proliferation (Vergouwen et al, 1991, J. Repord. Fertil), proteins are usually expected to be retained for a long time in the adult stages, even if the gene is deleted. Thus, it is highly possible that SCF proteins still exist in the knockout cells in the tissues tested.

Secondly, on the other hand, considering the phenotype of the Sox9-CreER mice in the light of above and the length of the spermatogenic epithelial cycle, the result that the mice have less than

© 2023. Published by The Company of Biologists under the terms of the Creative Commons Attribution License (https://creativecommons.org/licenses/by/4.0/).

one-fifth of the number of cells within two weeks of the first tamoxifen treatment is surprisingly fast.

Finally, the experiment on “niches” and the discussion that comes from it is ambiguous.
Tight junctions reportedly segment the plasma membrane of Sertoli cells (Franca et al. Andrology, 2016). The Sertoli cell polarization, such as basal and apical compartmentalization, is essential to maintain and interact with specific germ cell types in the adult. Since Sertoli cells display numerous attachments to other cells, this study lacks a precise localization analysis of SCF proteins in the polarized Sertoli cells. -Also, the authors did not pay any attention to functional differences between membrane- and soluble-type SCFs in this study.

3. Novelty and impact in the developmental field As I mentioned above, it is not possible to judge from this study whether or not SCF from cells other than Sertoli cells takes effect on germ cells. Furthermore, given that there are many studies of the Kit-SCF signal network between spermatogonia and Sertoli cells including mutant analyses for W and Steel locus (Brannan et al. 1992, Genes &Dev; Mithraprabhu and Loveland, 2009, Reproduction), this study cannot be viewed as new and informative in the field of development except for the scRNA-seq data as a resource.

4. Conclusion To improve this study, I would suggest the authors perform additional experiments as follows.
#1 The authors should analyze the phenotype of mice at 3 to 6 months after the first tamoxifen administration followed by repeated tamoxifen treatments.
#2 Please confirm the reduction of SCF protein at least in Sertoli cells (Sox9-CreER) and Leydig cells (KitMerCreMer) in the conditional KO mice.
#3 It is necessary to visualize SCF protein localization in Sertoli cells to discuss the niche. If IHC analysis for SCF does not work, mutant mice expressing tagged SCF would allow visualization of the precise localization of SCF proteins on the segmented Sertoli cell membrane, which would greatly help their evaluation for the “niche”, and subsequently increase the value of this work. Thus, it is worth generating the mice carrying the tagged SCF.

Second revision

Author response to reviewers' comments

Reviewer 1 Advance Summary and Potential Significance to Field:
This is technically sound paper that could be of interest for reproductive biologists working on the field of spermatogonial compartment regulation. Data described further highlights the involvement of SCF/Kit system in the paracrine control of spermatogenesis.

Reviewer 1 Comments for the Author:
The authors show that lack of SCF production specifically in Sertoli cells blocks differentiation of spermatogonia. Overall data support the conclusions and the role of SCF/Kit in the paracrine control of spermatogenesis. Nevertheless, the described phenotypes were expected based on previous work and they don't add significantly to our understanding of the spermatogonial stem cell niche.
Response: We think that there are several merits of our work that potentially contribute to the field of spermatogenesis:
1) We provided an scRNA-seq resource revealing the expression patterns of growth factors that regulate spermatogenesis in the testis.
2) We identified five cellular sources of SCF in the testis and provided functional evidence that Sertoli cells are the only physiological important source of SCF for spermatogenesis.
3) We provided an scRNA-seq-based mechanism for the spermatogenic defect in *Scf* mutant.
4) Our new data with $R26^{Scf220}$ mice provided a mechanism for the importance of anatomical location of Sertoli cells in regulating spermatogenesis.

© 2023. Published by The Company of Biologists under the terms of the Creative Commons Attribution License (https://creativecommons.org/licenses/by/4.0/).

Reviewer 2 Comments for the Author:

I am sorry that I go back and forth with the questions, but the authors want to shift the paradigm in the field changing the niche definition, so I cannot approve the current version until further details are clarified.
1) What is the new definition of the spermatogenic niche according to the authors? In all the references provided (and in general definition of the niche in all tissues), the closure to the vasculature is one of the conditions. Indeed, the current definition of the spermatogenic niche also includes the specific localization at the basement membrane and closure to the vasculature. If differentiated spermatogonia and spermatocytes can also be found at the niche according to the authors (and those cells move away from the basement membrane and blood vessels), what is the new definition of the testicular niche? It seems to me that the authors want to rename Sertoli cells that are known to be the nursing units, where all developing cells are embedded, and which produce important growth factors for the developing germ cells, as the niche. What is the importance of that and why to do so?
2) Please remove from abstract and the introduction the following statement:
"Stem cell factor (Scf), one of the essential niche factors". This information is introduced as a given one, and for the testes, SCF has not been considered as a niche factor until this study where authors aim to change this definition.
Response: The spermatogenic niche is usually defined as a place where spermatogonial stem cells (SSCs) reside and are fueled with growth factors from neighboring cells. Sertoli cells are known to be a niche for SSCs by secreting growth factors, such as GDNF (Science, PMID: 10688798). We showed that conditional deletion of *Scf* from Sertoli cells depleted differentiating spermatogonia but not SSCs (Fig. 6A-F). Conditional overexpressing of *Scf* from Sertoli cells increased spermatogenesis (Fig. 7A-L). Our new data revealed that overexpression of the membrane-bound Scf is sufficient to rescue the defects in *Amh-cre; Scf*$^{fl/fl}$ mice (Fig. S8B-J). Our data suggested that progenitors downstream SSCs also require a niche- like microenvironment. It will be interesting to investigate whether more mature germ cells, such as spermatocytes, also require a niche-like microenvironment for maintenance and/or maturation.
To avoid conflicting with the paradigm in the field, we removed the sentence"Stem cell factor (Scf), one of the essential niche factors" from the abstract as suggested, and revised other places in the text accordingly. The description above is included in the discussion section of our revised manuscript.

Reviewer 3 Comments

1. Quality of the data
The quality of the histological analyses is very good. The validation experiment of the lineage-specific knockout strategy was carefully done by using the reporter mice. The analyses for the next-generation sequencing, such as single-cell analyses, were performed intensively.
2. Completeness
Firstly, it is essential to confirm that the target protein was lost in the case of a conditional knockout study. However, this work lacks the evaluation of SCF protein reduction in the tissues analyzed. Furthermore, this work notes that the authors analyzed the phenotype of the conditional knockout mice after only two weeks of tamoxifen administration. Given that Sertoli cells terminally differentiate by around 17 days after birth and then stop proliferation (Vergouwen et al, 1991, J. Repord. Fertil), proteins are usually expected to be retained for a long time in the adult stages, even if the gene is deleted. Thus, it is highly possible that SCF proteins still exist in the knockout cells in the tissues tested.
Response: The reviewer raised a good point. Although we do not have a valid anti- SCF antibody to detect the half-life of SCF protein upon tamoxifen treatment, we showed that conditional deletion of *Scf* from Sertoli cells by Sox9-creER caused a severe defect in spermatogenesis at 2 weeks after tamoxifen treatment (Fig. S6B-L). These data provide functional evidence that most SCF protein is eliminated in Sertoli cells by 2 weeks after *Scf* gene knockout.
According to literatures (e.g., Chem Sci, PMC5975921), most proteins have a half-life of 4-14 hours in a cell. We have provided data that conditional deletion of *Scf* by Kit- creER, Sma-creER, Cdh5-creER and Tcf21-creER efficiently eliminated the *Scf* mRNA from Leydig cells, smooth muscle cells, endothelial cells and Tcf21^{+} cells, respectively (Fig. S2). In our previous manuscript, we showed

© 2023. Published by The Company of Biologists under the terms of the Creative Commons Attribution License (https://creativecommons.org/licenses/by/4.0/).

that conditional deletion of *Scf* by Kit-creER, Sma-creER, Cdh5-creER or Tcf21-creER did not significantly affect spermatogenesis at 2 weeks after tamoxifen treatment (Fig. 4C-Q). In our revised manuscript, we extended the time period after tamoxifen treatment to 4 weeks. As a result, we still did not observe any defects in spermatogenesis in these mutant mice (Fig. S3). These data largely excluded the possibility that the normal phenotypes in these mice are attributed to the residual SCF protein.

Secondly, on the other hand, considering the phenotype of the Sox9-CreER mice in the light of above and the length of the spermatogenic epithelial cycle, the result that the mice have less than one-fifth of the number of cells within two weeks of the first tamoxifen treatment is surprisingly fast.

Response: The dramatic defects in spermatogenesis in 6-week-old *Sox9-creER; Scf*$^{fl/fl}$ mice at 2 weeks after tamoxifen treatment surprised us as well. On one hand, these data suggested that SCF protein is effectively eliminated by 2 weeks after *Scf* gene knockout. On the other hand, these data suggested that *Scf* from Sertoli cells is pivotal for not only the development but also the rapid turnover of germ cells during young adulthood. Spermatogenesis is completely blocked without Sertoli cell-derived SCF. The reduction of the overall germ cells in these mice was proportional to the reduction of STRA8^{+} differentiating spermatogonia, which are the direct target of SCF-Kit signaling (Fig. S6F, J).

Finally, the experiment on "niches" and the discussion that comes from it is ambiguous. Tight junctions reportedly segment the plasma membrane of Sertoli cells (Franca et al. Andrology, 2016). The Sertoli cell polarization, such as basal and apical compartmentalization, is essential to maintain and interact with specific germ cell types in the adult. Since Sertoli cells display numerous attachments to other cells, this study lacks a precise localization analysis of SCF proteins in the polarized Sertoli cells. Also, the authors did not pay any attention to functional differences between membrane- and soluble-type SCFs in this study.

Response: As suggested, we have carefully revised our statements to soften the "niche" concept used in our manuscript. To our knowledge, nobody has ever visualized SCF localization in a cell. We had tried many commercially available anti-SCF antibodies. We had also generated anti-SCF antibodies by ourselves. However, none of them worked in immunostaining, or even in western-blot (data not shown). In fact, most of our knowledge about membrane- and soluble-type SCF is indirectly from genetic studies of *Scf* mutations (e.g., Cell, PMID: 1705866). While our paper was in submission, we generated a *R26*^{Scf220} mouse line, in which the membrane-bound form of *Scf* was conditionally overexpressed under the control of CAG promoter at the *R26* cassette (Fig. S8A). We showed that overexpression of the membrane-bound SCF is sufficient to rescue the severe spermatogenic defects of *Amh-cre; Scf*$^{fl/fl}$ mice (Fig. S8B-J), suggesting that membrane-bound SCF is the functional form. Considering the direct physical contact between Sertoli cells and spermatogonia, these data provided a mechanism why SCF from Sertoli cells, but not from other somatic cells in the testis, is physiologically important for spermatogenesis.

3 .Novelty and impact in the developmental field

As I mentioned above, it is not possible to judge from this study whether or not SCF from cells other than Sertoli cells takes effect on germ cells. Furthermore, given that there are many studies of the Kit-SCF signal network between spermatogonia and Sertoli cells, including mutant analyses for W and Steel locus (Brannan et al. 1992, Genes &Dev; Mithraprabhu and Loveland, 2009, Reproduction), this study cannot be viewed as new and informative in the field of development except for the scRNA-seq data as a resource.

Response: We think that there are several merits of our work that potentially contribute to the field of spermatogenesis:

1) We provided an scRNA-seq resource revealing the expression patterns of growth factors that regulate spermatogenesis in the testis.

2) We identified five cellular sources of SCF in the testis and provided functional evidence that Sertoli cells are the only physiological important source of SCF for spermatogenesis.

3) We provided an scRNA-seq-based mechanism for the spermatogenic defect in *Scf* mutant.

4) Our new data with *R26*^{Scf220} mice provided a mechanism for the importance of anatomical location of Sertoli cells in regulating spermatogenesis.

© 2023. Published by The Company of Biologists under the terms of the Creative Commons Attribution License (https://creativecommons.org/licenses/by/4.0/).

4.Conclusion
To improve this study, I would suggest the authors perform additional experiments as follows.
#1 The authors should analyze the phenotype of mice at 3 to 6 months after the first tamoxifen administration followed by repeated tamoxifen treatments.
Response: We agree with the reviewer that repeated tamoxifen treatment of the CreER mice for 3-6 months will yield most efficient elimination of SCF. However, according to literatures, long-term tamoxifen administration has a strong side effect on spermatogenesis (e.g., Sci Rep, PMID: 28827578). Empirically, mice become infertile after repeated tamoxifen treatment for a month. In our previous manuscript, we provided new data showing that conditional deletion of *Scf* by Kit-creER, Sma-creER, Cdh5-creER or Tcf21-creER did not significantly affect spermatogenesis at 4 weeks after tamoxifen treatment (Fig. S3). These data further supported our conclusion that SCF from non-Sertoli cells is dispensable for spermatogenesis.

#2 Please confirm the reduction of SCF protein at least in Sertoli cells (Sox9-CreER) and Leydig cells (KitMerCreMer) in the conditional KO mice.
Response: We agree with the reviewer that a valid anti-SCF antibody will be helpful for us, and probably for the whole field, to study the expression and function of SCF. In fact, we had tried many commercially available anti-SCF antibodies. We had also generated anti-SCF antibodies by ourselves. However, none of them turned out to be valid in immunostaining, or even in western-blot (data not shown).

The spermatogenic defects observed in 6-week-old *Sox9-creER; $Scf^{fl/fl}$* mice at 2 weeks after tamoxifen treatment (Fig. S6B-L) are functional evidence that SCF protein is largely eliminated in these mice.

We had provided data that Kit-creER eliminated most *Scf* mRNA in *Kit-creER; $Scf^{fl/fl}$* mice (Fig. S2A-B). We showed that these mice did not display significant spermatogenic defects at 2 and 4 weeks after tamoxifen treatment (Fig. 4F-H and S3A- C). It is unlikely that the residual SCF protein retains for a month in Kit^+ Leydig cells.

#3 It is necessary to visualize SCF protein localization in Sertoli cells to discuss the niche. If IHC analysis for SCF does not work, mutant mice expressing tagged SCF would allow visualization of the precise localization of SCF proteins on the segmented Sertoli cell membrane, which would greatly help their evaluation for the "niche", and subsequently increase the value of this work. Thus, it is worth generating the mice carrying the tagged SCF.
Response: This is a very good suggestion. In fact, we had attempted to generate anti- SCF antibodies, and to generate a C-terminal GFP-tagged *Scf* mice. However, due to technical difficulties, none of them succeeded. To our knowledge, nobody has ever visualized SCF localization in a cell. Most of our knowledge about membrane- and soluble-type SCF is indirectly from genetic studies of *Scf* mutations (e.g., Cell, PMID: 1705866). While our paper was in submission, we generated *$R26^{Scf220}$* mouse line, in which the membrane-bound isoform of *Scf* was conditionally overexpressed under the control of CAG promoter at the R26 cassette (Fig. S8A). We showed that overexpression of the membrane-bound SCF is sufficient to rescue the severe spermatogenic defects of *Amh-cre; $Scf^{fl/fl}$* mice (Fig. S8B-J), suggesting that membrane-bound SCF is the functional form. Considering the direct physical contact between Sertoli cells and spermatogonia, these data provided a mechanism why SCF from Seroli cells, but not from other somatic cells in the testis, is physiologically important for spermatogenesis.

Third decision letter

MS ID#: DEVELOP/2022/200706

MS TITLE: Sertoli cells are the unique source of stem cell factor for spermatogenesis

AUTHORS: Yi Jacky Peng, Xinyu Thomas Tang, Hui Sophie Shu, Wenjie Dong, Hongfang Shao, and Bo O Zhou

© 2023. Published by The Company of Biologists under the terms of the Creative Commons Attribution License (https://creativecommons.org/licenses/by/4.0/).

I have now received the referees' reports on the above manuscript, and have reached a decision. The referees' comments are appended below, or you can access them online: please go to BenchPress and click on the 'Manuscripts with Decisions' queue in the Author Area.

As you will see, reviewer 3's major concerns are not solved in the revised version. Beyond countless number of manuscripts describing the role for SCF during spermatogenesis, novelty of this study comes only from exclusion of other cell types than Sertoli cells as a major source of SCF. It is therefore critically important to demonstrate "SCF gene products" are depleted in respective cell types in a legitimate manner. I think that suggestions made by the reviewer 3 are reasonable and understandable. These concerns should be properly addressed before we can consider publication. If you are able to revise the manuscript along the lines suggested, which involve further experiments, I will be happy receive a revised version of the manuscript. Your revised paper will be re-reviewed by the reviewer 3, and acceptance of your manuscript will depend on your addressing satisfactorily the reviewer 3's concerns. Please also note that Development will normally permit only one round of major revision. If it would be helpful, you are welcome to contact us to discuss your revision in greater detail. Please send us a point-by-point response indicating your plans for addressing the referee’s comments, and we will look over this and provide further guidance.

Please attend to all of the reviewers' comments and ensure that you clearly highlight all changes made in the revised manuscript. Please avoid using 'Tracked changes' in Word files as these are lost in PDF conversion. I should be grateful if you would also provide a point-by-point response detailing how you have dealt with the points raised by the reviewers in the 'Response to Reviewers' box. If you do not agree with any of their criticisms or suggestions please explain clearly why this is so.

Reviewer 2

Advance summary and potential significance to field

I believe the study adds significant new information to what was already known about SCF during spermatogenesis. The authors demonstrate the requirement of the SCF produced specifically by Sertoli cells, perform analysis of the gene expression to better understand the mechanism underlying the spermatogenesis arrest. Their preliminary data suggest that membrane-bound isoform of the SCF may have the major role in spermatogenesis. Although this finding requires additional evaluation, the results may lead to new studies and advancement in the filed.
Comments for the author

Authors addressed my concerns by changing the statements regarding SCF being a niche factor. I think the authors did a satisfactory job addressing comments of other reviewers.

Reviewer 3

Advance summary and potential significance to field

I understand the authors made a lot of effort to revise their work. However, the authors should firstly confirm the underlying results carefully. In this manuscript, the evidence that the SCF derived from cells other than Sertoli cells upon spermatogonia differentiation is not clear and conclusive.
The importance and biological function of membrane SCF in Sertoli cells upon differentiating spermatogonia has been already known in detail – from the analyses of the W and Sl mutant mice, genetically engineered mice, and ingenious experiments with the well-known neutralizing antibody, ACK2, against c-Kit, as well as various types of in vitro assays.

Comments for the author

The reviewer disagrees, with all due respect, with the responses about the tamoxifen treatment and the necessity of the evaluation of gene deletion. The paper on the side effects of tamoxifen cited by the authors (Scientific reports, PMID:28827578) is unsuitable because tamoxifen is administered at juvenile stages, such as d16 and d21, in that paper. The authors' study requires the

© 2023. Published by The Company of Biologists under the terms of the Creative Commons Attribution License (https://creativecommons.org/licenses/by/4.0/).

administration of tamoxifen to adults. Since several reports about avoiding side effects have also been published, the authors should follow these procedures or set up the condition on their own by which they can prevent such side effects. Even though the mice may show a few side effect-like phenotypes comparison with controls provides evidence that the phenotypes are indeed derived from the cells in which the Scf gene is deleted.
Furthermore, many anti-SCF antibodies that work well for western blotting analyses have been published. Considering the proportion of Sertoli cells and Leydig cells in the testes, it is reasonably possible to measure the SCF protein levels for evaluating the KO effects.
Without confirmation of the SCF loss, the authors cannot make any solid conclusions.

In addition to the above comments, there are many concerns about their work.
The authors do not seem to present the data equitably. For example, in Figure S6D, most of the tubules in Sox9CreERT;Scffl/fl testes are intact and contain sperm inside. However the authors chose the one showing fewer germ cells for enlargement.

The results of Figure 7 are hard to understand. If the differentiation of c-Kit-positive spermatogonia kept accelerating from neonate and juvenile stages, why do more differentiated cells exist even at the 6-week-old adult stage?

I am not sure about the significance of the added FigS8 experiment. Firstly, although the authors used Amh-Cre, they treated the mice with tamoxifen at four weeks of age, which does not make sense at all. Secondary, the importance of membrane-SCF is already known by studies of Sld mice. The R26-Scf220 overexpression system in this study is rather problematic than the studies of Sld mice because it lacks a stage-specific expression regulation of SCF.

Overexpression experiments of Rosa-SCF by CreERT at the Sox9 or Cdh5 loci also do not make sense because the observation time point is too early for the abnormality to become visible. Spermatogenesis takes at least 8.6 days per cycle of stage progression and each seminiferous tubule is in a different stage. Therefore, even if the differentiation process is impaired in adult stages, the testicular phenotype is not usually expected to be obvious in two weeks after the tamoxifen treatment.

Third revision

Author response to reviewers' comments

Reviewer 3 Comments for the Author:
1. The reviewer disagrees, with all due respect, with the responses about the tamoxifen treatment and the necessity of the evaluation of gene deletion. The paper on the side effects of tamoxifen cited by the authors (Scientific reports, PMID:28827578) is unsuitable because tamoxifen is administered at juvenile stages, such as d16 and d21, in that paper. The authors' study requires the administration of tamoxifen to adults. Since several reports about avoiding side effects have also been published, the authors should follow these procedures or set up the condition on their own by which they can prevent such side effects. Even though the mice may show a few side effect-like phenotypes, comparison with controls provides evidence that the phenotypes are indeed derived from the cells in which the Scf gene is deleted.

Response: As suggested by the reviewer, we treated the conditional knockout mice and their littermates with tamoxifen once every other day from 4 to 12 weeks of age. The tamoxifen-treated testes appeared slightly smaller than untreated, but were generally normal. We did not detect obvious spermatogenic defects in Kit^{creER};$Scf^{fl/fl}$, $Cdh5^{creER}$;$Scf^{fl/fl}$, *Sma-creER*;$Scf^{fl/fl}$ or $Tcf21^{creER}$;$Scf^{fl/fl}$ mice, as compared with their littermate controls (Fig. S3). These new data were consistent with our conclusion that conditional knockout of *Scf* from Leydig, endothelial, vascular smooth muscle, or $Tcf21^{+}$ stromal cells does not impair spermatogenesis.

2. Furthermore, many anti-SCF antibodies that work well for western blotting analyses have been published. Considering the proportion of Sertoli cells and Leydig cells in the testes, it is

© 2023. Published by The Company of Biologists under the terms of the Creative Commons Attribution License (https://creativecommons.org/licenses/by/4.0/).

reasonably possible to measure the SCF protein levels for evaluating the KO effects. Without confirmation of the SCF loss, the authors cannot make any solid conclusions.

Response: As suggested by the reviewer, we tested several commercially available anti-SCF antibodies for western-blot. Although most of them did not work in our hand, we did get one valid anti-SCF antibody for western-blot. With this antibody, we showed that SCF protein was hardly detected in 6-week-old *Amh-cre;*$Scf^{fl/fl}$ testes (Fig. S2I). Similarly, SCF protein level in Leydig cells from Kit^{creER};$Scf^{fl/fl}$ testes was markedly reduced, compared with controls (Fig. S2J). The marked reduction of SCF protein levels in *Scf* conditional knockout mice was consistent with the marked reduction of *Scf* mRNA levels in these mice (Fig. S2B).

3. In addition to the above comments, there are many concerns about their work. The authors do not seem to present the data equitably. For example, in Figure S6D, most of the tubules in Sox9CreERT;Scffl/fl testes are intact and contain sperm inside. However, the authors chose the one showing fewer germ cells for enlargement.

Response: The reviewer is right that in the H&E staining data (Figure S6D), some tubules contained fewer germ cells than others in the same testis section from $Sox9^{creER}$;$Scf^{fl/fl}$ mice. This is likely because each seminiferous tubule is in a different stage and therefore is impaired to different extents by *Scf* conditional deletion. Nevertheless, germ cells in $Sox9^{creER}$;$Scf^{fl/fl}$ tubules were generally less frequent than control tubules (see figures below). Moreover, the tubular diameter of $Sox9^{creER}$;$Scf^{fl/fl}$ testes was obviously smaller than that of control mice (see low-magnification images). These data both suggested that the overall number of germ cells per tubule in $Sox9^{creER}$;$Scf^{fl/fl}$ testes is much less than controls. The H&E staining data were consistent with the anti-DDX4 staining data, in which different tubules from $Sox9^{creER}$;$Scf^{fl/fl}$ testes contained different numbers of $DDX4^+$ germ cells; nevertheless, the overall number of $DDX4^+$ cells per tubule was generally less than that in control testes (Figure S6G).

In our revised manuscript, we have replaced the enlarged image with new ones that including tubules containing different numbers of germ cells (Figure S6D).

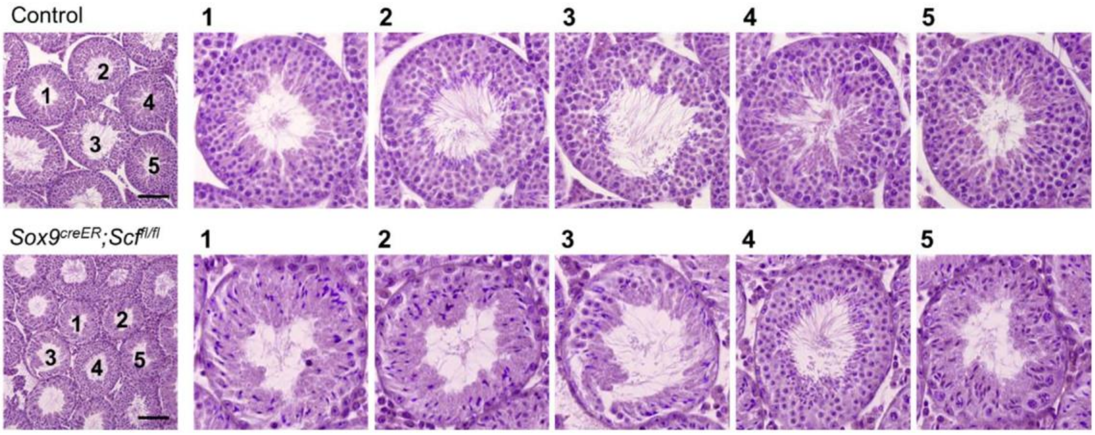

Figure. Enlarged images of individual tubules from $Sox9^{creER}$;$Scf^{fl/fl}$ and control testes.

4. The results of Figure 7 are hard to understand. If the differentiation of c-Kit- positive spermatogonia kept accelerating from neonate and juvenile stages, why do more differentiated cells exist even at the 6-week-old adult stage?

Response: We are not sure whether we understand the reviewer's question correctly. Is the reviewer asking why there were more differentiated cells exist in 6-week-old *Amh-cre;* $R26^{Scf}$ testes while there were few c-kit^+ spermatogonia? This is because spermatogonial stem cells are not affected by *Scf* overexpression, and therefore continuously supplement c-kit^+ spermatogonia.

© 2023. Published by The Company of Biologists under the terms of the Creative Commons Attribution License (https://creativecommons.org/licenses/by/4.0/).

These newly formed c-kit$^+$ spermatogonia are immediately driven by *Scf* overexpression to differentiate, resulting in more and more differentiated cells in the testis.

5. I am not sure about the significance of the added FigS8 experiment. Firstly, although the authors used Amh-Cre, they treated the mice with tamoxifen at four weeks of age, which does not make sense at all. Secondary, the importance of membrane-SCF is already known by studies of Sld mice. The R26-Scf220 overexpression system in this study is rather problematic than the studies of Sld mice because it lacks a stage- specific expression regulation of SCF.

Response: Firstly, we want to clarify that we did not treat these mice with tamoxifen, as *Amh*-Cre is a constitutive Cre. Secondly, we agree with the reviewer that because of a lack of stage-specific expression of *Scf*, some results are not fully conclusive. We have deleted these new data in our revised manuscript.

6. Overexpression experiments of Rosa-SCF by CreERT at the Sox9 or Cdh5 loci also do not make sense because the observation time point is too early for the abnormality to become visible. Spermatogenesis takes at least 8.6 days per cycle of stage progression and each seminiferous tubule is in a different stage. Therefore, even if the differentiation process is impaired in adult stages, the testicular phenotype is not usually expected to be obvious in two weeks after the tamoxifen treatment.

Response: The reviewer makes a good point. Spermatogenesis takes at least 8.6 days per cycle of stage progression and each seminiferous tubule is in a different stage. This is the reason that we have to examine the testis phenotypes in *Scf* conditional knockout mice at months after tamoxifen induction. However, in our opinion, it may be not necessarily applied to *Scf*-overexpressing mice because *Scf* overexpression exerts an active, rather than passive, effect on spermatogenesis, and thereby may accelerate spermatogenesis. Indeed, we observed elevated spermatogonial differentiation in 6- week-old $Sox9^{creER}$;$R26^{Scf}$ mice at 2 weeks after tamoxifen treatment (Fig. S8), although to a less extent than in *Amh-cre*;$R26^{Scf}$ mice. We did not observe any significant difference in spermatogenesis between 6-week-old $Cdh5^{creER}$;$R26^{Scf}$ and control mice at 2 weeks after tamoxifen treatment (Fig. 7N-V). In our revised manuscript, we have provided data on 12-week-old $Cdh5^{creER}$;$R26^{Scf}$ and control mice at 8 weeks after tamoxifen treatment. We still did not observe significant difference in spermatogenesis between these mice (Fig. S7F-L). These data suggested that overexpression of *Scf* in Sertoli cells, but not endothelial cells, affects spermatogenesis.

Fourth decision letter

MS ID#: DEVELOP/2022/200706

MS TITLE: Sertoli cells are the unique source of stem cell factor for spermatogenesis

AUTHORS: Yi Jacky Peng, Xinyu Thomas Tang, Hui Sophie Shu, Wenjie Dong, Hongfang Shao, and Bo O Zhou

I have now received all the referees' reports on the above manuscript, and have reached a decision. The referees' comments are appended below, or you can access them online: please go to BenchPress and click on the 'Manuscripts with Decisions' queue in the Author Area.

As you will see, reviewer 3 still expressed some significant criticisms and recommend a substantial revision of your manuscript before we can consider publication. She/he has a concern about "uniqueness" of Sertoli cells, which is a major conclusion of this study and seems to be not fully warranted in the revised version. If you are able to revise the manuscript along the lines suggested, which may involve further experiments, I will be happy receive a revised version of the manuscript. Your revised paper will be re-reviewed by one or more of the original referees, and acceptance of your manuscript will depend on your addressing satisfactorily the reviewers' major concerns. Please also note that Development will normally permit only one round of major revision. If it would be

© 2023. Published by The Company of Biologists under the terms of the Creative Commons Attribution License (https://creativecommons.org/licenses/by/4.0/).

helpful, you are welcome to contact us to discuss your revision in greater detail. Please send us a point-by-point response indicating your plans for addressing the referee's comments, and we will look over this and provide further guidance.

Please attend to all of the reviewers' comments and ensure that you clearly highlight all changes made in the revised manuscript. Please avoid using 'Tracked changes' in Word files as these are lost in PDF conversion. I should be grateful if you would also provide a point-by-point response detailing how you have dealt with the points raised by the reviewers in the 'Response to Reviewers' box. If you do not agree with any of their criticisms or suggestions please explain clearly why this is so.

Reviewer 2

Advance summary and potential significance to field

I believe the study adds significant new information to what is already known about SCF during spermatogenesis. Using a variety of genetic approaches , the authors demonstrate the requirement of SCF produced specifically by Sertoli cells to support spermatogenesis. They also and analyze the gene expression changes upon inactivation of such secretion to better understand the mechanism underlying the spermatogenesis arrest. it looks like SCF regulates the cell cycle and entrance into meiosis. Furthermore, over-expression of Sertoli-produced SCF seems to increase the number of spermatocytes and spermatids while depleting c-KIt-positivie spermatogonia that apparently being pushed into meiosis very fast.

Comments for the author

I think the authors did a satisfactory job addressing comments of other reviewers.
Couple minor things:

please add an explananation if exist to the following:

1) Fig. 1D-DDX4 is cytoplasmic; 1E-DDX4 looks nuclear
2) Fig. 3 G- c-kit looks cytoplasmic at Leydig cells; 3H-C-kit looks nuclear.

Reviewer 3

Advance summary and potential significance to field

It is already known that SCF produced by Sertoli cells is essential for male germ cell differentiation. Therefore, the point that the authors should have proven is to show SCF from cells other than Sertoli cells is not involved. To show this point clearly, it is essential to reveal efficient scf gene deletion experimentally and/or the loss of the SCF protein.
However, the most critical point has yet to be achieved.

Comments for the author

1. The reviewer disagrees, with all due respect, with the responses about the tamoxifen treatment and the necessity of the evaluation of gene deletion. The paper on the side effects of tamoxifen cited by the authors (Scientific reports, PMID:28827578) is unsuitable because tamoxifen is administered at juvenile stages, such as d16 and d21, in that paper. The authors' study requires the administration of tamoxifen to adults. Since several reports about avoiding side effects have also been published, the authors should follow these procedures or set up the condition on their own by which they can prevent such side effects. Even though the mice may show a few side effect-like phenotypes, comparison with controls provides evidence that the phenotypes are indeed derived from the cells in which the Scf gene is deleted.
Response: As suggested by the reviewer, we treated the conditional knockout mice and their littermates with tamoxifen once every other day from 4 to 12 weeks of age. The tamoxifen-treated testes appeared slightly smaller than untreated, but were generally normal. We did not detect obvious spermatogenic defects in KitcreER;Scffl/fl, Cdh5creER;Scffl/fl, Sma-

© 2023. Published by The Company of Biologists under the terms of the Creative Commons Attribution License (https://creativecommons.org/licenses/by/4.0/).

creER;Scffl/fl or Tcf21creER;Scffl/fl mice, as compared with their littermate controls (Fig. S3). These new data were consistent with our conclusion that conditional knockout of Scf from Leydig, endothelial, vascular smooth muscle, or Tcf21+ stromal cells does not impair spermatogenesis.

I understand the authors' efforts. However, in the case of conditional KO mice, gene deletion does not occur consistently, even among littermates, so it is essential to consider an experimental design that can evaluate the gene deletion efficiency and the loss of proteins in the target cells. These efforts are essential as basic/fundamental data to interpret the phenotypes. Without this, the authors cannot be sure that the SCF secreted by the cells of interest is not functioning. However, there is no assessment of the efficiency of gene deletion or the loss of SCF proiten in the target cells except for Sertoli cells and Leydig cells in the revised version of the manuscript. Without this, it is impossible to judge whether SCF protein from individual cells has not participated in germ cell development/differentiation. Since tdTomato is a very bright fluorescent protein, the data from Figure S2 do not help evaluate gene deletion efficiency.

2. Furthermore, many anti-SCF antibodies that work well for western blotting analyses have been published. Considering the proportion of Sertoli cells and Leydig cells in the testes, it is reasonably possible to measure the SCF protein levels for evaluating the KO effects. Without confirmation of the SCF loss, the authors cannot make any solid conclusions.

Response: As suggested by the reviewer, we tested several commercially available anti-SCF antibodies for western-blot. Although most of them did not work in our hand, we did get one valid anti-SCF antibody for western-blot. With this antibody, we showed that SCF protein was hardly detected in 6-week-old Amh-cre;Scf fl/fl testes (Fig. S2I). Similarly, SCF protein level in Leydig cells from KitcreER;Scffl/fl testes was markedly reduced, compared with controls (Fig. S2J). The marked reduction of SCF protein levels in Scf conditional knockout mice was consistent with the marked reduction of Scf mRNA levels in these mice (Fig. S2B).
I agree that SCF is efficiently lost in Sertoli cells and Leydig cells. Same comments as for Q1.

3. In addition to the above comments, there are many concerns about their work. The authors do not seem to present the data equitably. For example, in Figure S6D, most of the tubules in Sox9CreERT;Scffl/fl testes are intact and contain sperm inside. However, the authors chose the one showing fewer germ cells for enlargement.
Response: The reviewer is right that in the H&E staining data (Figure S6D), some tubules contained fewer germ cells than others in the same testis section from Sox9creER;Scf fl/fl mice. This is likely because each seminiferous tubule is in a different stage and therefore is impaired to different extents by Scf conditional deletion. Nevertheless, germ cells in Sox9creER;Scf fl/fl tubules were generally less frequent than control tubules (see figure in the pdf file of "response to the reviewer"). Moreover, the tubular diameter of Sox9creER;Scf fl/fl testes was obviously smaller than that of control mice (see low-magnification images). These data both suggested that the overall number of germ cells per tubule in Sox9creER;Scf fl/fl testes is much less than controls. The H&E staining data were consistent with the anti-DDX4 staining data, in which different tubules from Sox9creER;Scf fl/fl testes contained different numbers of DDX4+ germ cells; nevertheless, the overall number of DDX4+ cells per tubule was generally less than that in control testes (Figure S6G).
In our revised manuscript, we have replaced the enlarged image with new ones that including tubules containing different numbers of germ cells (Figure S6D).
Thank you. Nevertheless, why did the authors not perform 12 weeks-treatment of tamoxifen in Sox9creER;Scf fl/fl mice? The interpretation of the results is different from that of Amh-cre ;Scf fl/fl mice, which lose SCF gene from embryonic stages.

4. The results of Figure 7 are hard to understand. If the differentiation of c-Kit-positive spermatogonia kept accelerating from neonate and juvenile stages, why do more differentiated cells exist even at the 6-week-old adult stage?
Response: We are not sure whether we understand the reviewer's question correctly. Is the reviewer asking why there were more differentiated cells exist in 6-week-old Amh-cre; R26Scf testes while there were few c-kit+ spermatogonia? This is because spermatogonial stem cells are

© 2023. Published by The Company of Biologists under the terms of the Creative Commons Attribution License (https://creativecommons.org/licenses/by/4.0/).

not affected by Scf overexpression, and therefore continuously supplement c-kit+ spermatogonia. These newly formed c-kit+ spermatogonia are immediately driven by Scf overexpression to differentiate, resulting in more and more differentiated cells in the testis.
Thank you for the explanation.

5. I am not sure about the significance of the added FigS8 experiment. Firstly, although the authors used Amh-Cre, they treated the mice with tamoxifen at four weeks of age, which does not make sense at all. Secondary, the importance of membrane-SCF is already known by studies of Sld mice. The R26-Scf220 overexpression system in this study is rather problematic than the studies of Sld mice because it lacks a stage-specific expression regulation of SCF.
Response: Firstly, we want to clarify that we did not treat these mice with tamoxifen, as Amh-Cre is a constitutive Cre. Secondly, we agree with the reviewer that because of a lack of stage-specific expression of Scf, some results are not fully conclusive. We have deleted these new data in our revised manuscript.
In the paragraph the authors deleted in this version (in the former version of the manuscript, page 17, line17-20), the authors clearly stated that they treated 6-week-old Amh-cre; Scffl/fl; R26Scf220 mice with tamoxifen at 4 weeks of age.

6. Overexpression experiments of Rosa-SCF by CreERT at the Sox9 or Cdh5 loci also do not make sense because the observation time point is too early for the abnormality to become visible. Spermatogenesis takes at least 8.6 days per cycle of stage progression and each seminiferous tubule is in a different stage. Therefore, even if the differentiation process is impaired in adult stages, the testicular phenotype is not usually expected to be obvious in two weeks after the tamoxifen treatment.
Response: The reviewer makes a good point. Spermatogenesis takes at least 8.6 days per cycle of stage progression and each seminiferous tubule is in a different stage. This is the reason that we have to examine the testis phenotypes in Scf conditional knockout mice at months after tamoxifen induction. However, in our opinion, it may be not necessarily applied to Scf-overexpressing mice because Scf overexpression exerts an active, rather than passive, effect on spermatogenesis, and thereby may accelerate spermatogenesis. Indeed, we observed elevated spermatogonial differentiation in 6-week-old Sox9creER;R26Scf mice at 2 weeks after tamoxifen treatment (Fig. S8), although to a less extent than in Amh-cre;R26Scf mice. We did not observe any significant difference in spermatogenesis between 6-week-old Cdh5creER;R26Scf and control mice at 2 weeks after tamoxifen treatment (Fig. 7N-V). In our revised manuscript, we have provided data on 12-week-old Cdh5creER;R26Scf and control mice at 8 weeks after tamoxifen treatment. We still did not observe significant difference in spermatogenesis between these mice (Fig. S7F-L). These data suggested that overexpression of Scf in Sertoli cells, but not endothelial cells, affects spermatogenesis.
Same comments as for Q1.

Fourth revision

<u>Author response to reviewers' comments</u>

Q1: I understand the authors' efforts. However, in the case of conditional KO mice, gene deletion does not occur consistently, even among littermates, so it is essential to consider an experimental design that can evaluate the gene deletion efficiency and the loss of proteins in the target cells. These efforts are essential as basic/fundamental data to interpret the phenotypes. Without this, the authors cannot be sure that the SCF secreted by the cells of interest is not functioning. However, there is no assessment of the efficiency of gene deletion or the loss of SCF proiten in the target cells except for Sertoli cells and Leydig cells in the revised version of the manuscript. Without this, it is impossible to judge whether SCF protein from individual cells has not participated in germ cell development/differentiation. Since tdTomato is a very bright fluorescent protein, the data from Figure S2 do not help evaluate gene deletion efficiency.

Q2: I agree that SCF is efficiently lost in Sertoli cells and Leydig cells. Same comments as for Q1.

© 2023. Published by The Company of Biologists under the terms of the Creative Commons Attribution License (https://creativecommons.org/licenses/by/4.0/).

Response: In our revised manuscript, we have performed additional western blot experiments to evaluate the deletion efficiency of SCF protein in other targeted cell types. The data suggested SCF protein was hardly detected in endothelial cells from *Cdh5^creER^;Scf^fl/fl^* mice, SMA$^+$ vascular smooth muscle cells from *SMA-creER;R26^tdTomato^;Scf^fl/fl^* mice or Tcf21$^+$ stromal cells from *Tcf21^creER^; R26^tdTomato^ Scf^fl/fl^* mice (Fig. S2I), which were consistent with the marked reduction of *Scf* mRNA levels in these targeted cell types.

Q3: *Thank you. Nevertheless, why did the authors not perform 12 weeks-treatment of tamoxifen in Sox9creER;Scf fl/fl mice? The interpretation of the results is different from that of Amh-cre ;Scf fl/fl mice, which lose SCF gene from embryonic stages.*

Response: We introduced *Sox-creER* mice in order to investigate whether the differences in *Amh-cre; Scf^fl/fl^* mice and other *Scf* conditional konckout mice resulted from the timing of *Scf* conditional knockout. Therefore, we performed tamoxifen treatment on *Sox9-creER; Scf^fl/f^* mice at 4 weeks of age rather than at the embryonic stage, which was the same time points as other *Scf* conditional knockout mice. Comparing to *Amh-cre;Scf^fl/fl^* mice, we observed a similar phenotype of disrupted spermatogenesis in 6-week-old *Sox9^creER^; Scf^fl/fl^* mice (Fig. S5B-L). This result suggested that spermatogenic defects resulted from *Scf* conditional knockout could be observed as early as two weeks after tamoxifen treatment. However, we did observe a slighter phenotype of *Sox9^creER^;Scf^fl/fl^* mice (Fig. S5B-L) than *Amh-cre; Scf^fl/fl^* mice (Fig. 4E), which was consistent with the reviewer's opinion that two weeks may be too short to observe all potential effects of *Scf* conditional konckout. In our revised manuscript, we treated the *Sox9^creER^; Scf^fl/fl^* mice and their littermates with tamoxifen once every other day starting from 4 to 12 weeks of age. We found that all tubules in *Sox9^creER^; Scf^fl/fl^* testes were devoid of germ cells except for undifferentiated spermatogonia (Fig. S7C-K), which was similar to the phenotype that we observed in *Amh-cre; Scf^fl/fl^* testes. Together with our data in 12-weeks-old *Scf* conditional konckout mice (Fig. S3), these data suggested deletion of *Scf* from Sertoli cells, but not other *Scf*-expressing cells, block spermatogenesis.

Q4: Thank you for the explanation.

Q5: In the paragraph the authors deleted in this version (in the former version of the manuscript, page 17, line17-20), the authors clearly stated that they treated 6-week-old Amh-cre; Scffl/fl;R26Scf220 mice with tamoxifen at 4 weeks of age.

Response: The reviewer is right. We mentioned in the text (by mistake), although we did not perform the treatment. We thank the reviewer for pointing out this mistake.

Q6: Same comments as for Q1.

Response: In our revised manuscript, we have performed further western-blot experiments and confirmed that SCF protein was significantly overexpressed in endothelial cells from *Cdh5^creER^; R26^Scf^* testes than control testes (Fig. 7X). Thus, these results suggested overexpression of *Scf* in endothelial cells did not increase spermatogenesis.

Fifth decision letter

MS ID#: DEVELOP/2022/200706

MS TITLE: Sertoli cells are the unique source of stem cell factor for spermatogenesis

AUTHORS: Yi Jacky Peng, Xinyu Thomas Tang, Hui Sophie Shu, Wenjie Dong, Hongfang Shao, and Bo O Zhou

I have now received all the referees reports on the above manuscript, and have reached a decision. The referees' comments are appended below, or you can access them online: please go to BenchPress and click on the 'Manuscripts with Decisions' queue in the Author Area.

© 2023. Published by The Company of Biologists under the terms of the Creative Commons Attribution License (https://creativecommons.org/licenses/by/4.0/).

The overall evaluation is positive and we would like to publish a revised manuscript in Development, provided that the referees' comments can be satisfactorily addressed. Please attend to all of the reviewers' comments in your revised manuscript and detail them in your point-by-point response. If you do not agree with any of their criticisms or suggestions explain clearly why this is so. If it would be helpful, you are welcome to contact us to discuss your revision in greater detail. Please send us a point-by-point response indicating your plans for addressing the referee's comments, and we will look over this and provide further guidance.

Reviewer 2

Advance summary and potential significance to field

This study provides a novel information about the sources and effect of the SCF on spermatogenesis. While it was known that SCF is required for spermatogonia differentiation, this work supports the importance of Sertoli cells as a required source of SCF, and provides a better analysis of the pathways in spermatogonia affected by the absence of SCF.

Comments for the author

During the revisions, the authors have added different models, different lengths of tamoxifen treatment etc. However, material and method section doesn't describe these changes. The only statement about the treatment is "For induction of CreER activity, mice were treated with 1 mg tamoxifen (Sigma, T5648-5G) daily for 3 days". Although different timing/dosage was mentioned in the result section, the authors have to make sure they indicate the exact dose, timing and the age of the animals by the beginning of the treatment at the material and method section.

During the previous revision, I had two minor questions, but they have not been answered:

please add an explanation if exists to the following:
1) Fig. 1D-DDX4 is cytoplasmic; 1E-DDX4 looks nuclear 2) Fig. 3 G- c-kit looks cytoplasmic at Leydig cells; 3H-C-kit looks nuclear.

Reviewer 3

Advance summary and potential significance to field

The authors added new data, such as the loss of proteins due to SCF gene deletion in each cell type. The results confirmed that much of the SCF protein was lost in each tissue.
However, no methodological and stastical information was provided about the newly added data.

Comments for the author

Response to Q1 and Q2 about Figure S2I
1. Do Amh-cre;Scffl/fl mice and Cdh5creER;Scffl/fl mice have R26tdTomato alleles respectively? (Legend to Figure S2I)
2. Insufficient disclosure of the methodological and statistical data. How many cells or proteins did they apply for the western blot experiments? How many times did they perform the FACS sorting and the western blot experiments? If multiple FACS were performed, information such as the average positivity rate of the positive cells, if there was a difference between control and knockout, etc., is not shown and mentioned. These are the basic issues.
Response to Q3
SoxCreER in the title of Figure S6 should be Sox9CreER.

Response to Q6
No statistical information about the western blot for Figure 7X. Same issue as Q1 and Q2.

© 2023. Published by The Company of Biologists under the terms of the Creative Commons Attribution License (https://creativecommons.org/licenses/by/4.0/).

Fifth revision

Author response to reviewers' comments

Reviewer 2 Comments for the Author:

During the revisions, the authors have added different models, different lengths of tamoxifen treatment etc. However, material and method section doesn't describe these changes. The only statement about the treatment is "For induction of CreER activity, mice were treated with 1 mg tamoxifen (Sigma, T5648-5G) daily for 3 days". Although different timing/dosage was mentioned in the result section, the authors have to make sure they indicate the exact dose, timing and the age of the animals by the beginning of the treatment at the material and method section.
Response: For long-term induction of CreER activity, mice were treated with 1 mg tamoxifen once every two days from 4 to 12 weeks of age. We have added the information of tamoxifen treatment at the material and method section of our revised manuscript.

During the previous revision, I had two minor questions, but they have not been answered: please add an explanation if exists to the following:
1) Fig. 1D-DDX4 is cytoplasmic; 1E-DDX4 looks nuclear
2) Fig. 3 G- c-kit looks cytoplasmic at Leydig cells; 3H-C-kit looks nuclear.

Response: We are sorry for missing these questions in our last round of revision. The different localization of fluorescent signals in Fig. 2D, 2E, 3G and 3H is due to different labeling strategy. Fig 2D and 3G used anti-DDX4 and anti-c-kit immunostaining to indicate the cells that express DDX4 and c-kit, respectively, in the testis. Antibody immunostaining reflects the physiological localization of the protein of interest in the cell. DDX4 is a cytoplasmic protein. c-kit is a membrane-bound protein. Therefore, anti-DDX4 and anti-c-kit immunostaining detected fluorescent signals in the cytoplasm and membrane, respectively, but not in the nucleus (Fig. 2D and 3G). Of note, because anti-c-kit signal was so bright at membrane, it leaked into the cytoplasm (Fig. 3G).
In contrast, Fig. 2E and 3H used a Tomato reporter to indicates the cells that express DDX4 and c-kit, respectively, in the testis. Tomato protein does not indicate the localization of the gene of interest, instead, it spreads throughout the cell, including cytoplasm and nucleus (Fig. 2E and 3H).

Reviewer 3 Comments for the Author:
Response to Q1 and Q2 about Figure S2I
1.Do Amh-cre;Scffl/fl mice and Cdh5creER;Scffl/fl mice have R26tdTomato alleles, respectively? (Legend to Figure S2I)
Response: No, they do not. For *Amh-cre; $Scf^{fl/fl}$* and their control mice, whole testicular cells were prepared by an enzymatic digestion and centrifugation protocol that enriched Sertoli cells. For *kit^{creER}; $Scf^{fl/fl}$* and *$Cdh5^{creER}$; $Scf^{fl/fl}$* mice, biotin anti-c-kit microbeads and anti-CD31 microbeads were used to enrich c-kit$^+$ and Cdh5$^+$ cells, respectively. Currently there is no method available to directly enrich *SMA*$^+$ and *Tcf21*$^+$ stromal cells. Therefore, for *Sma^{creER}; $R26^{tdTomato}$; $Scf^{fl/fl}$*, *$Tcf21^{creER}$; $R26^{tdTomato}$; $Scf^{fl/fl}$*, and their control mice, *SMA*$^+$ and *Tcf21*$^+$ stromal cells were purified by FACS based on Tomato expression.

2.Insufficient disclosure of the methodological and statistical data. How many cells or proteins did they apply for the western blot experiments? How many times did they perform the FACS sorting and the western blot experiments? If multiple FACS were performed, information such as the average positivity rate of the positive cells, if there was a difference between control and knockout, etc., is not shown and mentioned. These are the basic issues.
Response: Each western-blot image was a representative from three independent experiments. No less than 200, 000 cells of interest were used for each western-blot experiment. For FACS sorting experiments, the purity (average positivity rate of the positive cells) was above 90%. We have added these information at the material and method section of our revised manuscript. We have also performed statistical analyses of the differences between control and knockout mice (see data in Fig. 7X,Y and S2I,J in our revised manuscript)

Response to Q3
SoxCreER in the title of Figure S6 should be Sox9CreER.

© 2023. Published by The Company of Biologists under the terms of the Creative Commons Attribution License (https://creativecommons.org/licenses/by/4.0/).

Response: Thank the reviewer for pointing out the mistake. We have corrected this mistake in our revised manuscript.

Response to Q6
No statistical information about the western blot for Figure 7X. Same issue as Q1 and Q2.
Response: We have added statistical information in our revised manuscript (Fig. 7Y).

<u>Sixth decision letter</u>

MS ID#: DEVELOP/2022/200706

MS TITLE: Sertoli cells are the unique source of stem cell factor for spermatogenesis

AUTHORS: Yi Jacky Peng, Xinyu Thomas Tang, Hui Sophie Shu, Wenjie Dong, Hongfang Shao, and Bo O Zhou
ARTICLE TYPE: Research Article

I am happy to tell you that your manuscript has been accepted for publication in Development, pending our standard ethics checks.

© 2023. Published by The Company of Biologists under the terms of the Creative Commons Attribution License (https://creativecommons.org/licenses/by/4.0/).
